# Measles Infection Dose Responses: Insights from Mathematical Modeling

**DOI:** 10.1007/s11538-024-01305-0

**Published:** 2024-06-09

**Authors:** Anet J. N. Anelone, Hannah E. Clapham

**Affiliations:** Saw Swee Hock School of Public Health, National University of Singapore, 12 Science Drive 2, Singapore, 117549 Singapore

**Keywords:** Measles, Infection dose, Mathematical modeling, Dose-response, Cellular immunity, Virus-host interactions

## Abstract

**Abstract:**

How viral infections develop can change based on the number of viruses initially entering the body. The understanding of the impacts of infection doses remains incomplete, in part due to challenging constraints, and a lack of research. Gaining more insights is crucial regarding the measles virus (MV). The higher the MV infection dose, the earlier the peak of acute viremia, but the magnitude of the peak viremia remains almost constant. Measles is highly contagious, causes immunosuppression such as lymphopenia, and contributes substantially to childhood morbidity and mortality. This work investigated mechanisms underlying the observed wild-type measles infection dose responses in cynomolgus monkeys. We fitted longitudinal data on viremia using maximum likelihood estimation, and used the Akaike Information Criterion (AIC) to evaluate relevant biological hypotheses and their respective model parameterizations. The lowest AIC indicates a linear relationship between the infection dose, the initial viral load, and the initial number of activated MV-specific T cells. Early peak viremia is associated with high initial number of activated MV-specific T cells. Thus, when MV infection dose increases, the initial viremia and associated immune cell stimulation increase, and reduce the time it takes for T cell killing to be sufficient, thereby allowing dose-independent peaks for viremia, MV-specific T cells, and lymphocyte depletion. Together, these results suggest that the development of measles depends on virus-host interactions at the start and the efficiency of viral control by cellular immunity. These relationships are additional motivations for prevention, vaccination, and early treatment for measles.

**Graphical abstract:**

Measles infection dose responses: insights from mathematical modeling. Top: Model-data fits for acute viremia in response to changes in measles infection doses. $10^4$, $10^3$, $10^2$, 10 and 1 TCID_50_ correspond to red diamonds, blue stars, orange triangles, magenta dots, and green squares respectively. The solid lines represent the trajectories generated by the proposed model parameterization. The shapes represent data. The dark grey dotted dashed line represents the limit of detection < 0.3. Bottom: Cartoon illustrating that when the measles infection dose increases, the stimulation of the measles-specific cellular immune responses increases early on post-infection. This enhanced immune response reduces the time required to clear the infectious viral load and helps maintain similar levels of viral loads and lymphocyte depletion irrespective of the initial dose.

**Supplementary Information:**

The online version contains supplementary material available at 10.1007/s11538-024-01305-0.

## Introduction

Pathogens such as viruses enter the body in varying quantities, influencing the development of diseases (Yezli and Otter 2011; Ward et al. 1984). The infection dose defines the quantity of pathogens at the start. This initial dose is an important quantity related to the magnitude and time course of viral loads and immune responses, which may have bearings on onset, transmission, vaccination, severity, morbidity, and mortality (Yezli and Otter 2011; Li and Handel 2014; Handel et al. 2018a). Though the importance of the infection dose is well-recognized in the literature, there is a lack of experimental and mathematical studies (Yezli and Otter 2011; Li and Handel 2014). The infection dose is still challenging to measure and estimate in vitro, in vivo, and in sillico (Yezli and Otter 2011; Holder and Beauchemin 2011). The infection dose is often quantified using cell culture infectivity assays, where the number of infectious virions are estimated using the dilution of virus sufficient to cause cytopathic effect i.e. changes in cell morphology, physiology or death in $50\%$ of the inoculated culture ($TCID_{50}$) (van Binnendijk et al. 1994; Yezli and Otter 2011; Ward et al. 1984). However, it remains unclear how many infectious or non infectious virions are exactly present in one $TCID_{50}$ (Yezli and Otter 2011). Some studies suggest that a single infectious virion is sufficient to establish infection, other studies suggest *Allee effects*, where the number of infections virions must exceed a certain threshold, and others suggest random effects (Yezli and Otter 2011; Perelson and Nelson 1998). Additionally, infection dose responses can differ depending on the pathogen, the host, the infection route, age, and other factors (Li and Handel 2014; Yezli and Otter 2011). Thus, gaining more insights into virus-host mechanisms underlying infection dose responses has a strong potential to inform prevention, treatments, clinical management and public health (Aaby and Coovadia 1985). This holds significant relevance in the context of the measles virus (MV), since changes in MV infection doses influence the establishment and progression of measles (van Binnendijk et al. 1994; Aaby 1995, 1992; Aaby and Coovadia 1985).

MV is an enveloped, non-segmented, negative sense RNA infective microorganism, that belongs to the genus Morbillivirus in the family Paramyxoviridae (WHO 2023; Griffin 2020). MV is highly transmissible in the air and on contaminated surfaces (Norrby 1985; WHO 2023; Griffin 2020, 2021). Wild type MV infects about $90\%$ of exposed susceptible unvaccinated individuals (WHO 2023; McLean et al. 2013). Measles starts with a clinically asymptomatic incubation period, on average 14 days (range: 7–21 days) from exposure to the appearance of the characteristic measles rash all over the body (WHO 2023; McLean et al. 2013). Individuals infected by MV are contagious 4 days before up to 4 days after the rash appears (WHO 2023; McLean et al. 2013).

Wild type MV also leads to immunosuppression and lymphopenia i.e. an abnormally low number of lymphocytes in the blood, typically from 7 days post infection, and lymphocyte counts recover over few weeks (WHO 2023; Lin et al. 2012; Griffin 2021). MV-induced immunosuppression renders the host vulnerable to opportunistic infection such as pneumonia (WHO 2023; Griffin 2021). MV can cause severe disease, complications, and death, mostly among unvaccinated children under 5 years old and pregnant women (WHO 2023; McLean et al. 2013; Aaby 1991). Since 1963, measles can be prevented using two doses of safe and cost-effective MV-containing vaccines such as the measles, mumps, and rubella vaccine MMR II, containing $\ge 10^3$ TCID_50_ live attenuated measles virus (Enders’ attenuated Edmonston strain) (WHO 2023; McLean et al. 2013; Commonwealth of Australia and Care 2023). MV-containing vaccines are also recommended by the World Health Organization (WHO) as post-exposure prophylaxis within 72*h* to prevent and mitigate measles (WHO 2023; McLean et al. 2013; Arciuolo et al. 2017a). However, measles cannot yet be prevented, nor treated using any licensed antiviral drugs such as remdesivir (WHO 2023; Peart Akindele et al. 2023; McLean et al. 2013; Arciuolo et al. 2017b). In 2021, measles related death were about 128000 globally (WHO 2023). Various issues impaired measles surveillance, vaccination, and clinical management due to the COVID-19 pandemic (WHO 2023). These key facts motivate efforts to improve understanding of the pathogenesis of measles.

Measles pathogenesis is still the subject of active research, and some controversies (Griffin 2020, 2021; Lin et al. 2021). Since MV yields pathogenesis in cynomolgus and rhesus macaques similar to those observed in humans, cynomolgus and rhesus macaques are preferentially used as animal models in several experimental studies on measles (van Binnendijk et al. 1994; Auwaerter et al. 1999; El Mubarak et al. 2007). It is currently unclear how wild type MV establishes infection in the respiratory track, progresses in the lymphoid tissues, and spreads subsequently throughout the body (Griffin 2020, 2021; Lin et al. 2021). Current findings suggest that Wild type MV infects and replicates initially in respiratory differentiated epithelial cells through the apical surface; apical MV infection produces viable MV-infected multinucleated giant cells (MGCs), and MGCs leave rapidly the epithelial surface intact while migrating quickly to the lumen of the respiratory tract (Lin et al. 2021). Subsequently, MV-infected MGCs are likely to be phagocytized by alveolar macrophages, resulting in the infection of immune cells expressing the CD150+ (SLAMF1) receptor in the lung and local lymph nodes (Lin et al. 2021; Suwanmanee et al. 2023; Griffin 2021). On the one hand, MV blocks innate immune responses with the inflammatory cytokines such as types I and III interferons (IFNs). On the other hand, MV elicits innate immune responses with $NF-K \beta$ signalling and inflammasome activation by pro- and anti-inflammatory macrophages (Suwanmanee et al. 2023). However, MV-induced innate immune responses fail to affect intracellular viral replication, and to kill MV-infected cells such as macrophages (Suwanmanee et al. 2023; Shivakoti et al. 2015; Fontana et al. 2008). MV produces cell-associated viremia with viral budding from the plasma membrane, and no virus-induced cell death (Lin et al. 2021; Suwanmanee et al. 2023; Griffin 2021; Mcchesney and Oldstone 1989; Haspel et al. 1977). Though MV-induced innate immune responses fail to prevent the systemic spread of MV, these responses are likely to contribute to the MV-specific T cell responses associated with clearance of infectious viral load, and the appearance of the rash (Permar et al. 2003, 2004; Suwanmanee et al. 2023; Griffin 2021). Infectious viral particles are no more detected from peripheral blood mononuclear cells (PBMCs) after about 15 days post infection, but MV RNA remains detectable in PBMCs, lymph nodes, urine and nasopharyngeal secretions (Lin et al. 2012; Griffin 2021). Although MV targets B cells and antibodies, MV also induces specific antibody responses which become detectable after the peak of infectious viremia, increase up to a plateau, suppress MV-RNA in blood and tissues, and slowly decrease over the years, thereby contributing to long-term immunity against MV (Lin et al. 2012; El Mubarak et al. 2007; Griffin 2021). Such complex feedbacks between MV and immune responses motivate mathematical modeling of within-host measles dynamics (Lin et al. 2012; Anelone et al. 2021; Morris et al. 2018).

Mathematical modeling has delivered substantial qualitative and quantitative insights into the pathogenesis of virus such as the Human Immunodeficiency Virus (HIV), Lymphocytic choriomeningitis virus (LCMV), dengue, and SARS-CoV-2 (Perelson and Ribeiro 2013; Sariol and Perlman 2020; Mayer et al. 2019; Clapham et al. 2014; Anelone and Spurgeon 2016). The mathematical modeling studies in Handel et al. (2018b); Li and Handel (2014) revealed that infection dose response patterns could be explained by mathematical formulations which provide insights for vaccine design, and specific mechanisms underlying virus control by immune responses. Yet, mathematical modeling on within-host MV is only delivered by the following four publications according to our literature survey, namely (Heffernan and Keeling 2008; Lin et al. 2012; Morris et al. 2018; Anelone et al. 2021).

The work in HeffernanandKeeling (2008) excludes model-data fitting, and assumes that MV-specific immune cells remained uninfected, thus neglecting the paradoxical positive feedback of immune activation on measles viral growth (De Swart et al. 2007; Morris et al. 2018). The work in Lin et al. (2012)

calibrates a simple model to study the long-term control of persistent MV RNA by the T cell and antibody immune responses in rhesus macaques. The work in Morris et al. (2018) provides a mechanistic Ordinary Differential Equations (ODEs) model which fits reasonably well longitudinal virological and immunological data on acute measles infection in rhesus macaques. This ODE model incorporates MV infection of lymphocytes, including activated MV-specific T cells, and death of MV infected cells due to MV-specific T cell killing, natural effects and virus-induced effects (Morris et al. 2018). The work in Anelone et al. (2021) applied control theory to analyze on the model in (Morris et al. 2018), and delivers analytical conditions under which T-cell killing becomes the primary mechanism for immunosuppression and viral clearance. The results in Morris et al. (2012; 2018; 2021) are in line with experimental findings supporting that T-cell-mediated immunity controls infectious viral load during acute MV infection (Permar et al. 2003, 2004). However, previous mathematical studies did not focus on MV infection dose responses, thus motivating the present work.

Here, we investigate mechanisms underlying wild type measles infection dose responses. We built this work on previous animal and mathematical studies on measles. We used seminal longitudinal data on experimental acute infection of cynomolgus monkeys with different doses of wild type MV from (van Binnendijk et al. 1994), data that had never been previously incorporated into mathematical modeling studies. We evaluated different model parameterizations supporting relevant biological hypotheses to describe the data. This work helps evaluating hypotheses on virus-host interactions underlying the development and control of acute measles infection.

## Methods

###  Data on Measles Infection Dose Responses

In seminal studies (van Binnendijk et al. 1994), 14 cynomolgus monkeys were infected intratracheally with different MV doses, and the subsequent acute measles infection evaluated. The infection doses were $10^4$, $10^3$, $10^2$, $10^1$, $10^0$, and $10^{-1}$ Tissue Culture $50\%$ Infectious Dose (TCID_50_) of the cell-free wild-type Bilthoven measles virus strain (MV-BIL). The cynomolgus monkeys were 20–30 months old, and on average three monkeys were infected with each infectious dose. We used the mean of the measurements at each day for each infection doses. Successful MV-infection was confirmed by performing virus isolation through cocultivation of PBMC, lung lavage cells, and pharyngeal epithelial cells with human B-lymphoblastoid cell line (B-LCL) JP. Cocultures were visually monitored for cytopathic changes over 10 days, and the presence of MV-infected cells in cultures was demonstrated by immunofluorescence staining, and a T cell proliferation assay with MV-specific human T cell clones. The studies in van Binnendijk (1994) recorded the average number of MV-infected cells per $10^6$ peripheral blood mononuclear cells (PBMC) at different days within 18 days post-infection. As successful MV-infection resulted in an extensive PBMC-associated viremia, the recorded number of MV-infected cells per $10^6$ PBMC is a proxy used to monitor infectious viral load (Morris et al. 2018; Lin et al. 2012).

### Mathematical Modeling of Measles Infection Dose Responses

We described interactions between MV and lymphocytes using ODEs, to study how acute measles infection develops following different infection doses. In particular, we aim to explain and fit data on the impacts of MV infection doses on cell-associated acute viremia, reported in van Binnendijk et al. (1994). We formulated different model parameterizations to test hypotheses on biological mechanisms which keep the magnitude of the viral load peak relatively constant, but delay the peak of the viral load, when the MV inoculum dose decreases (van Binnendijk et al. 1994).

We restricted our simulations and analyzes to the first 30 days post infection, since acute infectious virions remain undetectable afterwards in similar studies (Lin et al. 2012).

Since the ODE model with T cell killing formulated in Morris et al. (2018) fits well data on acute MV infection in rhesus macaques from Lin et al. (2012), this is a suitable model parameterization, as cynomolgus monkeys and rhesus macaque share similar acute MV infection dynamics (El Mubarak et al. 2007). The ODEs from Morris et al. (2018) describe the infection of measles specific activated T cells (*A*), and other susceptible lymphocytes (*S*), by MV infectious particles (*V*), at a rate $\beta$. Susceptible lymphocytes (*S*), proliferate at a rate $q_s$, when the proliferation function $\omega (t)=1$ during the first $t_d$ days of infection. To match the experimental settings in van Binnendijk et al. (1994), the model has the initial conditions $V(0) \ne 0$, for the infection dose, $I(0)=0$, to account for primary measles infection, $S(0) \ne 0; A(0) \ne 0$, to account for the presence of susceptible lymphocytes and precursor MV-specific T cells at the start. Measles infected lymphocytes, *I*, produce new virions at a rate *p* and die at a rate $\delta$ due to natural and MV infection-induced cell death. New virions are removed from the blood stream at a rate *c*. Measles virus triggers the proliferation of the measles-specific activated T cells. The measles specific T cell response is governed by the saturation function *f*(*V*) where *s* is the saturation constant. When $f(V)=1$, measles-specific activated T cells proliferate at a rate *q*. Measles specific activated T cells kill infected lymphocytes via a cytolytic killing process, *K*(*I*, *A*), described by mass-action kinetics with *k* as the killing rate. When $f(V)=0$, measles specific activated T cells die at a rate *d* and generate measles-specific memory cells at a rate *r* during this contraction phase. The dynamical equations are:

1 $$\begin{aligned} \frac{d A}{dt}&= - \beta A V + q f(V) A - (1-f(V) )( d+r) A , \end{aligned}$$

2 $$\begin{aligned} \frac{d S}{dt}&= -\beta S V + q_s \omega (t) S + r(1- f(V) ) A, \end{aligned}$$

3 $$\begin{aligned} \frac{d I}{dt}&= \beta (A+S) V - \delta I - k(I,A), \end{aligned}$$

4 $$\begin{aligned} \frac{d V}{dt}&= p I - c V , \end{aligned}$$

where

5 $$\begin{aligned} \omega (t)&= \left\{ \begin{array}{lcl} 1 &{} \text {if} &{} t < t_d , \\ 0 &{} \text {if} &{} t \ge t_d, \end{array} \right. \end{aligned}$$

6 $$\begin{aligned} f(V)&= \frac{V}{s + V}, \end{aligned}$$

and

7 $$\begin{aligned} K(I,A)&= K I A. \end{aligned}$$

Since measles exhibits cell-associated viremia with viral budding and no virus-induced cell lysis (Lin et al. 2021; Suwanmanee et al. 2023; Griffin 2021; Mcchesney and Oldstone 1989; Haspel et al. 1977), and since experimental and mathematical studies suggest that T-cell killing is the predominant mechanism controlling lymphocyte depletion and viral clearance during uncomplicated acute MV infection (Permar et al. 2003, 2004; Lin et al. 2012; Morris et al. 2018; Anelone et al. 2021), we formulated the alternative model parameterization (B) by setting $\delta =0$, to cancel the lysis of lymphocytes by MV, and to test T cell killing as the only mechanism for lymphocyte depletion and viral clearance.

To assess the impacts of MV infection doses, we evaluated additional model parameterizations (A1) and (M1) in the simple scenario, where MV infection doses influence only the initial viral load, *V*(0). Furthermore, we evaluated the model parameterizations (A2 and B2), in the scenario where MV infection doses influence both the initial viral load, *V*(0), and the number of activated T cells *A*(0). We summarized the model parameterizations in Table 1.

**Table 1:** Summary of the different model parameterizations with their respective assumptions on virus-host interactions to describe measles infection dose responses

Model parameterizations	Characteristics and biological assumptions
A1	Model (1)–(7) (Morris et al. 2018)
A2	Model A1 where A(0) changes for each dose
B1	Model A1 with no MV-induced infected cell death $\delta = 0$
B2	Model B1 where A(0) changes for each dose

###  Model-Data Fitting

To estimate the best-fitting parameters, we fit the proposed model parameterizations in Table 1 to the infectious viral load data from van Binnendijk et al. (1994). We used the maximum likelihood estimation approach as in (Morris et al. 2018), and assumed log-normally distributed residuals. Since some viral load measurements were recorded below the limit of detection, we handled these censored data by applying a well-known approach used in previous mathematical modeling work on virus dynamics (Handel et al. 2018b, 2010; Pawelek et al. 2016; Schröter et al. 2020). When any model predicts that the viral load *V* is less than the limit of detection on given measurement days, we set the corresponding residual error to zero, to consider no penalty for this difference. We calculated the likelihood using the logged values of the model output of state variable *V* and infectious viral load data D:

8 $$\begin{aligned} RSS_V&= \sum _{i=1}^{n_V}( ln V_{i}^M - ln V_{i}^D )^2, \end{aligned}$$

where $n_V$ is the number of measurements for the infectious viral load. The corresponding log-likelihood expression for *V* is

9 $$\begin{aligned} Ln \hat{L}_X&= - n_X ln( \sqrt{ 2 \pi } \sigma _X ) -\frac{ RSS_X}{2 \sigma _X } \end{aligned}$$

Next, we solved the equation

10 $$\begin{aligned} \delta _{\sigma _{X}^2} Ln \hat{L}_X&= -\frac{ n_X }{ \sigma _X } + \frac{ RSS_X}{\sigma _X^3 } = 0, \end{aligned}$$

to obtain the maximum likelihood estimate for $\sigma _{X}^2$ so that

11 $$\begin{aligned} \sigma _{X}^2&= \frac{ RSS_X }{ n_X }. \end{aligned}$$

We reformulated the log-likelihood expression by substituting (9) into (9): after simplification:

12 $$\begin{aligned} Ln \hat{L}_X&= - \frac{ n_X}{2} ln( \frac{ 2 \pi }{n_X } RSS_X ) - \frac{n_X}{2}. \end{aligned}$$

When we fit the different model parameterizations to the infectious viral load data for all infection doses,

the full log-likelihood is

13 $$\begin{aligned} Ln \hat{L}_{1}&=\sum _{i=1}^{5} Ln \hat{L}_{v_I}, \end{aligned}$$

the sum of the log-likelihood (12) for the infectious viral load measured for the inoculum $10^4$, $10^3$, $10^2$, $10^1$, and $10^0$, and $10^{-1}$ (TCID_50_). When we fit the different model parameterizations to the data from (van Binnendijk et al. 1994), since the data does not contain measurements on the total lymphocyte count and MV-specific T cell responses in (van Binnendijk et al. 1994), we fixed *L*(0), $q_s$, $t_d$, *q*, *r*, $d_A$, and *c*. When $10^4$ TCID_50_ MV-BIL is used to infect the macaques in (van Binnendijk et al. 1994) and (Morris et al. 2018), the measured viremia in our study provides a match of superior quality to the measured and estimated viremia from the rhesus macaque 46u in (Morris et al. 2018), as compared to the other macaques in (Morris et al. 2018). For instance, the day and magnitude of the peak viremia in our study best match the ones for macaque 46u in (Morris et al. 2018). Consequently, we set the values of the above fixed parameters to best-fitting parameter values of the model (1)–(4) to the data of the rhesus macaque 46u, from Table 2 and 3 in Morris et al. (2018).

## Results

### Salient Features of MV Infection Dose Responses

We revisited longitudinal measles data from van Binnendijk et al. (1994) to summarize and estimate dynamical features, reflecting the impacts of MV infection doses on PBMC-associated infectious acute viremia.

When MV infection dose is $10^{-1}$ TCID_50_, acute viremia remains below the limit of detection i.e. $\le 0.3$ $log_{10} TCID_{50}$ per $10^6$ PBMCs, suggesting insufficient infectious virions to establish infection, or rapid viral clearance in tissues by the immune system (Zhu et al. 1997; Lemon et al. 2011).

**Fig. 1:**
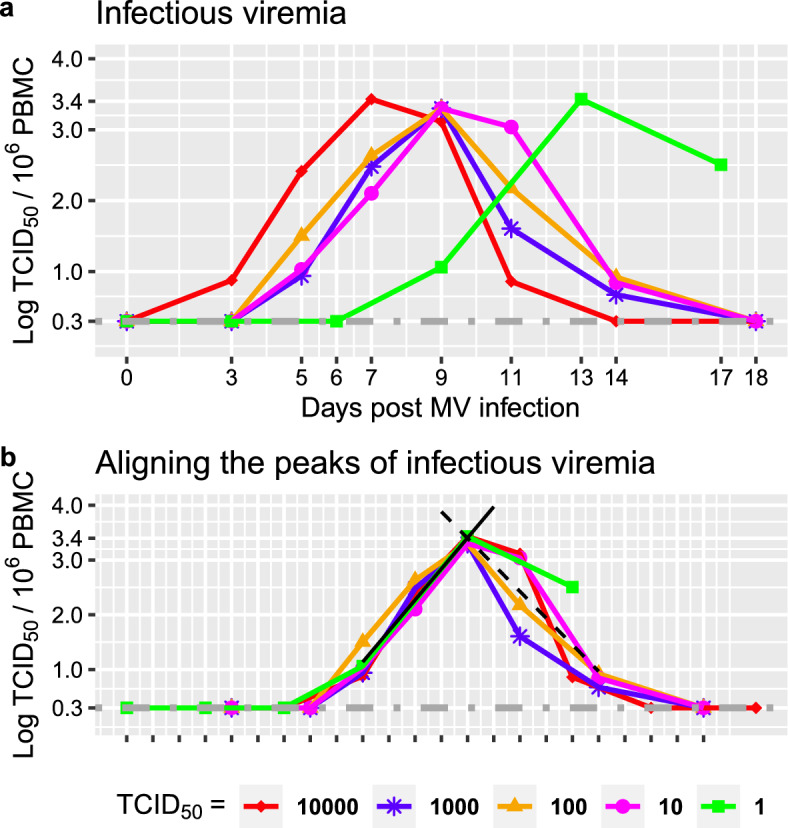
Salient features of MV infection dose responses. **a** Time course of PBMC-associated acute viremia following wild type measles infection of cynomolgus monkeys with different doses. The different colours and points represent different TCID_50_ for infection. The dark grey dotted dashed line represents the limit of detection $$< 0.3$$. Data from (van Binnendijk et al. 1994). **b** MV infection dose responses where the peaks of viremia are aligned. Each black tick corresponds to a day on the x-axis. The oblique block line is the linear regression to estimate the growth rate $$r_d = 0.56$$ $$ LogTCID_{50}/10^6$$ PBMCs per day, $$S.D. = 0.03 $$, $$p<0.001$$ with intercept $$-1.69$$ $$LogTCID_{50}/10^6$$ PBMCs, $$S.D.= 0.23$$, $$p<0.001$$. We considered significance levels with a p-values $$<0.05$$. The oblique dashed block line is the linear regression for the decline rate $$\delta _d = -0.48 $$ $$LogTCID_{50}/10^6$$ PBMCs per day, $$S.D. = 0.06 $$, $$p<0.001$$, with intercept 9.77 $$LogTCID_{50}/10^6$$ PBMCs, $$S.D.= 0.79$$, $$p<0.001$$

In contrast, when MV infection doses are $\ge 1$ TCID_50_, viremia increases up to a peak before decreasing to undetectable levels (Fig. 1a), suggesting a threshold or stochastic processes by which low numbers of infectious MV particles facilitate the establishment and transmission of MV infection (Norrby 1985; Laksono et al. 2016). Since the viremia remains below the limit of detection in the peripheral blood for at least the first 3 days post-infection for all infection doses, this suggest that infectious MV particles are lost in large numbers during intratracheal infection, or absent in the peripheral blood likely due to intracellular delays, slow transfer from the respiratory route, and MV sequestration in lymphoid tissues (Lemon et al. 2011; Griffin 2020); alternatively, infectious MV particles could be present in small numbers, and increasing or fluctuating in PBMCs below the limit of detection, as observed for HIV dynamics (Fiebig et al. 2005; Kahn and Walker 1998).

When MV infection dose increases for $\ge 1$ TCID_50_, we observe synchronisation between MV growth and immune control, since the day of the first detectable viremia and to the day of peak viremia decrease nonlinearly in identical quantities i.e. $-4$ days from 1 TCID_50_ to $10 \le TCID_{50} \le 10^3$, and $-2$ days from $10^3$ to $10^4$ TCID_50_ (Fig. 1), suggesting dose-dependent virus-host interactions in the blood and tissues while viremia is still undetectable during the early days post infection, and this is in agreement with epidemiological findings where high MV exposure is associated with short MV incubation period, early rash mediated by early cellular immunity (Aaby 1992, 1995, 1991). When MV infection dose increases for $\ge 1$ TCID_50_, the peak viremia remains almost constant at about 3.3 $TCID_{50}$ per $10^6$ PBMCs, supporting the notion that cellular immunity plays the role of a predictable and robust control mechanism to achieve clearance of acute viremia (Anelone et al. 2021) (see Fig. 1a, b).

When acute viremia is above the limit of detection i.e.$$ \le 0.3$$ $$log_{10} TCID_{50}$$ per $$10^6$$ PBMCs, acute viremia increases and decreases almost linearly on the log scale, with almost symmetrical slopes for the growth and decline, and almost the same slopes for different infection doses. Consequently, we performed linear regressions and estimated the corresponding viral growth rate, $$r_d = 0.56$$ $$ LogTCID_{50}/10^6$$ PBMCs per day, $$p<0.001$$, and decline rate, $$\delta _d = -0.48$$ $$ LogTCID_{50}/10^6$$ PBMCs per day, $$p<0.001$$, irrespective of MV infection doses. This suggests that when viremia is above the limit of detection, MV growth and clearance are independent of the antigen stimulation induced by MV infection doses (see Fig. 1b). Consequently, MV infection doses $$ \le 10^4 $$ and $$ \ge 1$$ TCID_50_ lead to similar duration for detectable viremia, approximately 8 to 9 days (Fig. 1b), and similar area under the curve (AUC) for acute viremia, $$AUC= 4.598$$ on average, suggesting similar MV exposure and effects in the peripheral blood irrespective of the infection doses. Together, these salient features of MV infection dose responses suggest that measles exhibits dose-dependent virus dynamics in the early days post infection, followed by dose independent viral growth, where the peak viremia and viral clearance are predictably regulated by consistent adaptive immune responses. Thus, these findings also motivate additional studies on early measles dynamics.

### MV Infection Dose Responses are Appropriately Described by Early Virus-Host Interactions

**Fig. 2:**
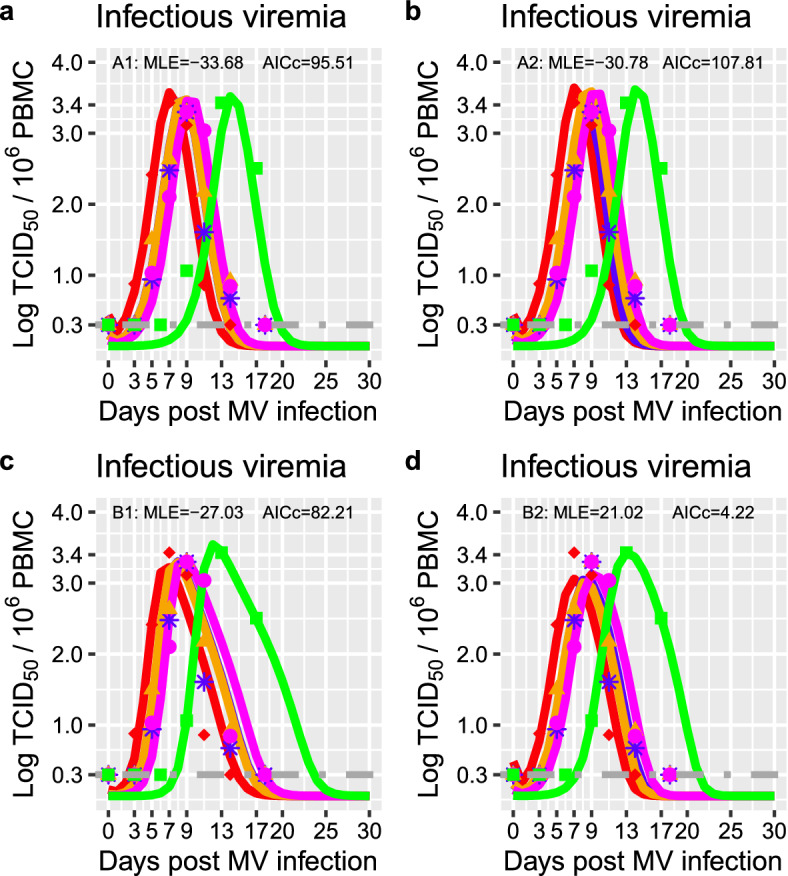
Evaluation of relevant hypotheses on measles infection dose responses using their corresponding model parameterization. Model-data fits for acute viremia in response to changes in MV infection doses using model parameterizations and assumptions in Table 1. Top left **a** Model (1)–(4) with parameterization A1 and changes in *V*(0). $10^4$, $10^3$, $10^2$, 10 and 1 TCID_50_ correspond to red diamonds, blue stars, orange triangles, magenta dots, and green squares respectively. The solid lines represent the trajectories generated by the model. The shapes represent data from (van Binnendijk et al. 1994). The dark grey dotted dashed line represents the limit of detection $< 0.3$. MLE stands for the maximum likelihood estimates. AICc stands for the Akaike Information Criterion corrected for small sample sizes. Top right **b** Model parameterization B1 for changes in *V*(0) and *A*(0). Bottom left **c** Model parameterization A2 combines model A1 with $\delta = 0$. Bottom right d: Model parameterization B2 combines model B1 with $\delta = 0$

**Fig. 3:**
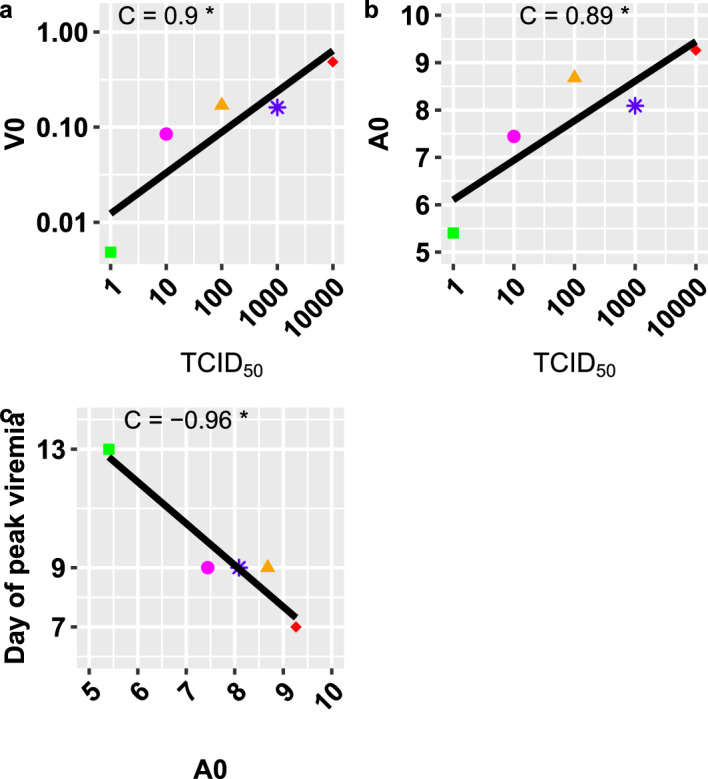
Relationships between key parameters. **a** Linear regression correlation between the infection doses and the initial viral load *V*(0). The different shapes and colours represent different infection doses. The black line represents the linear regression line. *C* stands for the correlation coefficient. $$*$$ stands for the p-values $$<0.05$$ for significance. The linear regression slope $$ = 3.95e-05 $$, $$S.D. = 7.82e-06$$, $$p<0.05$$, with intercept $$=9.32e-02$$, $$S.D.= 0.03$$, $$p=0.07$$. **b** Linear regression and correlation between the infection doses and the initial number of activated MV-specific T cells *A*(0). The linear regression slope $$ = 0.00019 $$, $$S.D. = 0.00015 $$, $$p=0.2$$, with intercept $$=7.33 $$, $$S.D.=0.71 $$, $$p<0.001 $$. c Linear regression and correlation between the number of activated MV-specific T cells *A*(0) and the day of the peak viremia. The linear regression slope $$ = -1.40 $$, $$S.D. = 0.00015 $$, $$p<0.05$$, with intercept $$=20.33 $$, $$S.D.=0.25 $$, $$p<0.01 $$

To describe and evaluate mechanisms underlying MV infection dose responses, we formulated different biological hypotheses on virus-host interactions, and fit the corresponding model parameterizations in Table 1 to the data to test these hypotheses. In particular, we considered changes in the initial number of activated T cells to account for changes in the recruitment and activation of MV-specific immune responses. We also considered virus budding with no virus-induced infected cell death with $\delta =0$, to match virological data. The model-data fits reproduced the patterns of MV infection dose responses (Fig. 2). Since the highest log-likelihood and the lowest AICs are generated by the model B2 (Fig. 2, this model is the most parsimonious model to describe the data on MV infection dose responses, supporting virus budding ($\delta =0$) and dose-dependent changes in the initial viral load and the initial number of activated T cells. Fitting the data to model B2 produces the best-fitting parameter values listed in Table 2, and the profile likelihoods in Fig. 5. As expected, MV infection dose is positively correlated with the initial viral load *V*(0) ($C=0.9$, $p= 0.039$) (see Fig. 3). MV infection dose is also positively correlated with the initial number of activated T cells *A*(0) ($C=0.89$, $p= 0.044$) (see Fig. 3). When the initial number of activated T cells *A*(0) increases, the time to the peak viral load decreases ($C=-0.96$, $p= 0.011$) (Fig. 3), supporting experimental findings in Permar et al. (2003, 2004). Together, these results suggest that the higher the infection doses, the higher the stimulation and recruitment of the MV-specific immune responses.

**Table 2:** Summary of the components of the model (1)–(4) with model parameterization B2

Name	Unit	Symbol	Value
Susceptible lymphocytes	cells/$\mu$L	*S*	$L(0)-A(0)$
Infected lymphocytes	cells/$\mu$L	*I*	0 Fixed
MV-specific activated T cells	cells/$\mu$L	*A*	
		$A_{10000}$	9.26
		$A_{1000}$	8.08
		$A_{100}$	8.67
		$A_{10}$	7.44
		$A_{1}$	5.40
Infectious viral load	$\log \text {TCID}_{50}$/$10^6$ PBMC	*V*	
		$V_{10000}$	0.48
		$V_{1000}$	0.16
		$V_{100}$	0.17
		$V_{10}$	0.084
		$V_{1}$	0.0048
Total lymphocyte count	cells/$\mu$L	$L=S+I+A$	Fixed
			3906
Infection rate	($\log \text {TCID}_{50}$/$10^6$ PBMC)^-1^ day^-1^	$\beta$	0.25
General lymphocyte proliferation rate	day^-1^	$q_s$	Fixed
			0.028
General lymphocyte proliferation function	dimensionless	$\omega (t)$	
Duration of general lymphocyte proliferation	day	$t_d$	Fixed
			2.6
Proliferation function of activated T cells	dimensionless	*f*(*V*)	
Saturation constant of MV-specific T cell proliferation	$\log \text {TCID}_{50}$ / $10^6$ PBMC	*s*	0.23
Conversion rate of MV-specific activated T cells into MV-specific memory T cells	day^-1^	*r*	Fixed
			0.016
Death rate of infected lymphocytes	day^-1^	$\delta$	0
Killing rate of activated T cells	(cells/$\mu$L)^-1^ day^-1^	*k*	0.00071
Proliferation rate of MV-specific activated T cells	day^-1^	*q*	Fixed
			1.11
Death rate of activated T cells	day^-1^	*d*	Fixed
			0.025
Viral replication rate	$\frac{\log \text {TCID}_{50}\text { / }10^6 \text { PBMC}}{(\text {cells/}\mu \text {L}) \text { day}}$	*p*	0.0030
Viral clearance rate	day^-1^	*c*	Fixed
			3

### MV Dose-Dependent Time Shifts on Immune Control and Immunosuppression

**Fig. 4:**
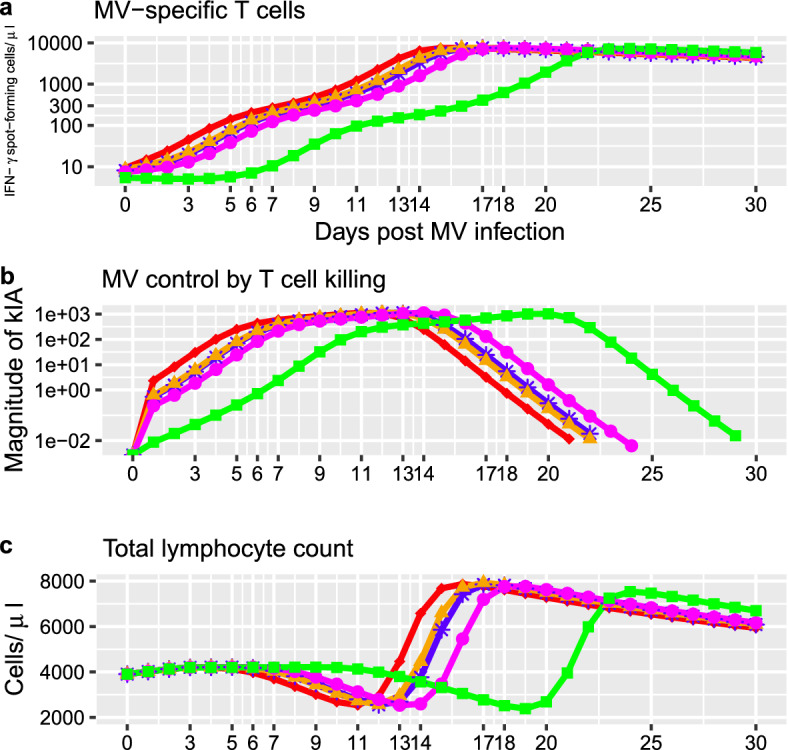
Simulations of the MV-specific T cell response, and lymphopenia for different MV infection doses using the model B2. **a** Time course of the MV-specific T cell response, state variable (*A*). The different colours and shapes represent the simulations for different MV infection doses. **b** Time course of the T cell killing action *kIA*. **c** Time course of the total lymphocyte count ($L=S+I+A$)

**Fig. 5:**
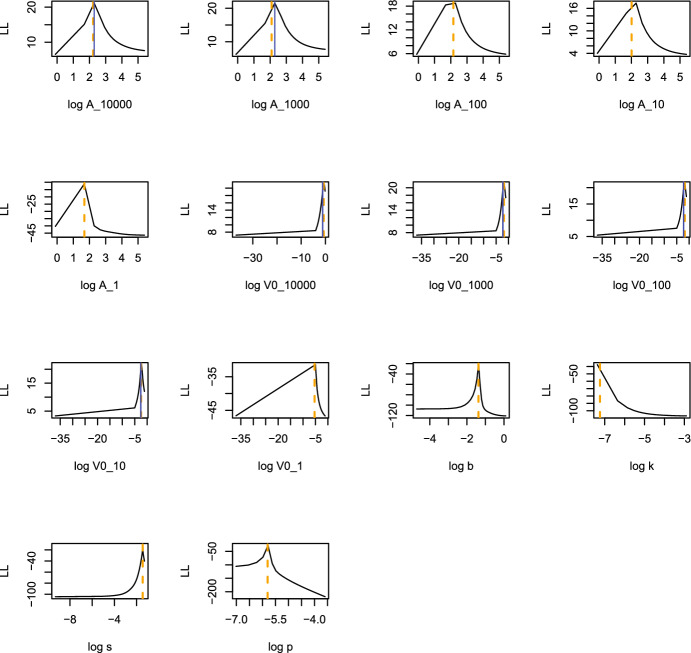
Likelihood profile for the estimated parameters for model B2, (1)–(4) with changes in *V*(0) and *A*(0) and $\delta = 0$. The black curve represents the Likelihood profile. The vertical orange dashed line corresponds to the maximum likelihood parameter estimate, listed in Table 2. The vertical blue line corresponds to the $95 \%$ confidence intervals, calculated using the likelihood ratio test

Since the data in van Binnendijk et al. (1994) does not provide measurements of lymphocyte dynamics for MV infection doses, we investigated the impacts MV infection doses on lymphopenia and the MV-specific T cell response for different infection doses using simulations from model B2. When MV infection dose increases, the time to the peak of activated MV-specific T cells and the peak of killing increase, and their peak magnitudes remain almost constant (Fig. 4a, b), suggesting that MV infection doses influence the expansion and killing action of the MV-specific T cell responses, such that on the day of peak viremia (day 7,9,13) similar number of activated T cells are involved in viral clearance (Fig. 4a, b). In contrast, the infection dose seems to have negligee effects on the contraction of MV-specific T cells, see Fig. 4a. Consequently, when the infection dose increases, the transient phase of lymphopenia occurs earlier but the magnitude of lymphocyte depletion remains relatively constant (Fig. 4c), suggesting that infection dose is not associated with the severity of lymphopenia. Together, these simulation results suggest that the observed dose-dependent patterns are similar for both acute viremia, immune control and immunosuppression.

## Discussion

We have performed mathematical studies on MV infection dose responses reported in van Binnendijk et al. (1994). Our mathematical work contributes to identify and link kinetic parameters and dynamical behaviours, which are dose-dependent or dose-independent. This work also delivers a more realistic model parameterization (Fig. 2), namely B2 in Table 1 and 2, which agrees with no MV-induced infected cell death, MV virions exit through budding processes (Lin et al. 2021; Suwanmanee et al. 2023), and control of acute infectious viremia by T cell immunity (Permar et al. 2004; Lin et al. 2012; Morris et al. 2018; Anelone et al. 2021). Our work predicts that variations of MV infection doses lead to similar changes in timings rather than magnitudes for both acute viremia and lymphocyte dynamics (Figs. 1- 4). MV infection doses have negligible impacts on detectable acute viremia i.e. negligible impacts on the area under the curves of viremia, and on corresponding viral growth and decline (Fig. 1 and Table 3). This work also infers MV dose dependent impacts on virus-host interactions in tissues during the early days preceding detectable viremia post-infection. This work adds that MV infection doses induce proportional changes in the initial viral load, and the initial number of activated T cells (Fig. 2 and Fig. 3). Thus, MV dose-dependent changes, which occur early on post infection for MV absorption and corresponding stimulation of immune cells, determine the onset and time course of viremia and cellular immunity, where the later enforces dose-independent magnitudes for viremia and lymphopenia. Changes in the timings but not the magnitude of viremia and lymphopenia suggest that the higher the MV infection doses, the earlier the onset of MV transmission, and the earlier clinical signs and symptoms such as fever and rash.

We obtained similar viremia and corresponding AUC as compared to those in El Mubarak et al. (2007), where cynomolgus and rhesus macaques were experimentally infected with the wild-type MV-Bil at $10^3$ $TCID_{50}$ (see table 1 in El Mubarak et al. (2007)). The peak viremia occurs on the same day and at the same magnitude for the infection dose $10^3$ TCID_50_ in (El Mubarak et al. 2007) and our data from (van Binnendijk et al. 1994). Wild type MV strains influence either or both the day and the magnitude of the peak PBMC-associated viremia at a given dose, as observed with MV-Sudan in (El Mubarak et al. 2007) or MV-Edmonston and MV-Schwartz in (van Binnendijk et al. 1994). Since some measles strains reproduce human measles in non-human primates (Zhu et al. 1997; Lin et al. 2012; van Binnendijk et al. 1994; El Mubarak et al. 2007; Laksono et al. 2016), our results could be applicable to both human measles and experimental measles infection of non-human primates. Additional research is required to perform comparative studies between infection dose responses from the wild type and the vaccine strains of MV, since the wild type and the vaccine strains of MV have different pathogenesis (Griffin 2018).

This work builds on the previous data-driven mathematical modeling studies in Lin et al. 2012, Morris et al. 2018 and Anelone et al. 2021. The viral load is qualitatively similar for both our data from (van Binnendijk et al. 1994), and that in Morris et al. (2018), where a high single infection dose of wild-type MV-Bil at $10^4$ TCID_50_ was used for experimental measles in rhesus macaques. In particular, the viral load peaks on day 7 post-infection, at 3.43 $log_{10} TCID_{50}$ per $10^6$ PBMCs, on average for 3 cynomolgus macaques used in (van Binnendijk et al. 1994), and 3.64 $log_{10} TCID_{50}$ per $10^6$ PBMCs, on average for 7 rhesus macaques used in (Morris et al. 2018), and subsequently remains undetectable from day 14 onwards. Unlike the data from Morris et al. (2018), our data from (van Binnendijk et al. 1994) contains censored measurements; the viremia remains at zero in Morris et al. (2018) before the peak and after viral decline. When viremia increases, the data from van Binnendijk et al. 1994 has one more measurement i.e. on day 5, as compared to the data from Morris et al. 2018. When viremia decreases, the data from van Binnendijk et al. 1994 has two additional measurements i.e. on day 9 and 11, as compared to the data from Morris et al. 2018. Unlike the data from Morris et al. 2018, the viral load becomes detectable from day 3 in our data from (van Binnendijk et al. 1994). These discrepancies are common, since quantitative differences in virus dynamics have been reported in the literature when experimental measles infection of macaques are conducted under different settings (El Mubarak et al. 2007; Zhu et al. 1997). In contrast, when experiments are conducted in the same settings for a given measles strain at $10^3$ TCID_50_, the investigations in El Mubarak et al. 2007 concluded that cynomolgus and rhesus macaques exhibit similar qualitative and quantitative dynamics for infectious viremia and lymphopenia. Thus, this support our approach to leverage relevant biological and mathematical findings from Lin et al. (2012), Morris et al. (2018) and Anelone et al. (2021).

Unlike in Morris et al. (2018), we do not assess different formulations for the dynamics of susceptible lymphocytes and MV-specific T cells due to the absence of relevant measurements, model parsimony, and the sparsity of our viral load data. Since virus dynamics are similar in Morris et al. (2018) and in van Binnendijk et al. (1994), and the total lymphocyte count and cellular immunity are measured in Morris et al. (2018) but not in our data from van Binnendijk et al. (1994), we used the model parameterization in (Morris et al. 2018) to help with model calibration by fixing the values of relevant parameters and initial conditions. Since the viral load is higher in van Binnendijk et al. (1994) than Morris et al. (2018) during viral growth, we set our fitting algorithm to search within parameter spaces wider than those suggested in Morris et al. (2018). On the one hand, our estimates for the virus replication rate *p* is lower than those in Morris et al. (2018). On the other hand, our estimates are higher for the initial viral load, and the infection rate $\beta$, as compared to those in Morris et al. (2018). Our estimates for the initial number of activated T cells is higher than the corresponding median values in Morris et al. (2018), in agreement with our findings showing that when the initial viral load increases, the initial number of activated T cells increases. Though our estimates for the T cell killing rate *k* and T cell proliferation saturation constant are respectively below and above their corresponding range in Morris et al. (2018), our model parameterization B2 outputs similar magnitudes and time courses for the MV-specific T cells, T cell killing, and the total lymphocyte count as compared to those in Morris et al. (2018) and Anelone et al. (2021), in agreement with our findings in Fig. 4. Together, our estimates suggest higher levels of measles uptake and dissemination compared to prior model parameterization in Morris et al. (2018). This is consistent with experimental data showing early detection of measles growth in van Binnendijk et al. (1994); El Mubarak et al. (2007) as compared to those in Morris et al. (2018).

The best fitting model parameterization from Morris et al. (2018) does not qualitatively reproduce the reported MV infection dose responses, unless one adjusts appropriately both the initial viral load, and the initial number of activated T cells. Note that the work in Morris et al. (2018) estimated both the initial viral load, and the initial number of activated T cells for each macaque. These observations helped formulating hypotheses in Table 1 on the relationships between MV infection dose, the initial viral load, and the initial number of activated T cells. This relationship is supported by our results for the AICc despite the sparsity of the data. Since AICc values decrease when our fitting algorithm estimates both the initial viral load and the initial number of activated T cells for each infection dose. Furthermore, our AICc values experience a more pronounced decrease, when we excluded virus-induced infected cell death. This was not tested in Morris et al. (2018). The work in Morris et al. (2018) did not estimate the infected cell death rate from the measles data, but instead used an estimate from previous mathematical modeling work on HIV. Our AICc results are in line with experimental work supporting that the measles virus does not directly induce death of infected lymphocytes by lysis.

Our results are also consistent with T cell depletion experiments in rhesus macaques and mathematical studies, since higher acute infectious viremia and delayed viral clearance were reported when for a given infection dose, the number of T cells, including activated MV-specific T cells, was significantly reduced, and T cell activation and proliferation where inhibited from the start up to 4 days post infection (Permar et al. 2003; Morris et al. 2018). Mathematical modeling of measles infection dose responses reveals the importance of cellular immunity in regulating the peak viremia and lymphopenia for different doses. According to control systems theory (Anelone et al. 2021; Astrom 1994; Edwards and Spurgeon 1998), T cell killing exhibits adaptive control strategies, through the adjustment of the immune stimulation and activation with respect to a given dose. The antigen independent phase of the T cell proliferation function in our study contributes to the production of a sufficient killing action to enforce viral clearance from similar peaks for different infection doses, thus allowing the T cell mediated control of MV infection to exhibit robust control performance in presence of variations in MV infection doses (Anelone et al. 2021).

Acute viremia and lymphopenia share similar dynamical responses to MV infection doses, since T cell killing of infected lymphocytes is the means to control acute viremia (Anelone et al. 2021). On the one hand, this is beneficial to mitigate quickly potential adverse effects due to high MV infection doses. On the other hand, this is detrimental because this establishes a similar period of immune vulnerability for any successful wild type MV infection, thereby allowing high chances of morbidity and mortality for children. Strong immune responses at low infection doses have also been observed for influenza infection in mice (Powell et al. 2006). The relationship between MV infection doses and lymphopenia is an additional motivation for prevention, vaccination, and early treatment for measles. From a public health standpoint, MV infection dose responses suggest that reducing the infection dose provides additional days to vaccinate, and to identify and isolate exposed individuals before they could transmit MV. Delays in MV dynamics due to low doses suggest that prophylaxis treatment could be successful beyond the recommended 72 h following exposure (WHO 2023; McLean et al. 2013), as observed in (Arciuolo et al. 2017a), thus motivating research to extend the recommended 72 h window to take advantage of the dynamics of low dose MV infections. Furthermore, our work shows similarities between the impacts of MV infection doses and the effects of post-exposure prophylaxis (PEP) with remdesivir on PBMC-associated viremia and on lymphocyte counts (Peart Akindele et al. 2023). In particular, when PEP with remdesivir is imitated from day 3 to day 12 post infection rhesus macaques, the day of the peak viremia vary but the magnitude of the peal viremia and lymphocyte counts remain similar. Thus, our modeling framework suggest that PEP with remdesivir influences initial amounts of infectious viremia and the corresponding immune stimulation, however, the resultant effects are insufficient and/or too late to prevent disease progression.

Epidemiological studies in children in Aaby (1992, 1995) and Aaby and Coovadia (1985) indicate that high exposure to measles led to early rash and more severe lymphopenia. Although it is unclear how exposure to MV relates to MV infection dose, our modeling framework suggest a positive correlation between MV exposure and infection dose, since the observed early onset of the rash would match early peak viremia and early viral control due to high infection dose. Since our results predict dose-independent lymphopenia, we hypothesize that the positive correlations between exposure, dose, lymphopenia, and morbidity could emerge during severe measles due to immune responses perturb by opportunistic infections such as pneumonia. In contrast, one could hypothesize trade-offs between rapid immune mediated viral control and high lymphopenia at high infection doses, with have bearings on the severity, morbidity, and mortality of measles, as observed for influenza infection in mice (Powell et al. 2006).

Early onset of clinical symptoms is also observed when the infection dose increases for pneumonia virus of mice (PVM) in mice (Horsfall Jr and Ginsberg 1951), adenovirus type 5 (ADV) infection of cotton rats (Prince et al. 1993; Li and Handel 2014), and influenza A virus (IAV) PR8 strain infection in mice (Ginsberg and Horsfall Jr 1952; Handel et al. 2018b). PVM infection doses influence the timings but not the magnitude of lung damage and pneumonia (Horsfall Jr and Ginsberg 1951). PVM infection doses do not influence the initial viral growth rate and the rate of increase in the amount of pneumonia. However, when PVM infection dose is very low and below a certain threshold, the peak viremia and the magnitude of pneumonia decrease (Horsfall Jr and Ginsberg 1951). It is unclear whether such phenomena happens at very low MV infection doses between 1 and 0.1 $TCID_50$. Unlike measles, the higher the PVM infection dose, the higher the PVM accumulation (AUC) (Horsfall Jr and Ginsberg 1951), suggesting that PVN infection dose influences the decline rate of acute viremia, and virus control by the immune system.

Measles infection dose responses also share some similarities with foot-and-mouth disease virus (FMDV) in pigs and infectious bronchitis virus (IBV) (Callison et al. 2006; Li and Handel 2014; Quan et al. 2004; Howey et al. 2009). Model-data fitting in Howey et al. (2009) show a proportional relationship between the FMDV infection dose and the initial viral load in the peripheral blood. In particular, the modeling work in Howey et al. (2009) used a Hill function to describe the infection process with saturation in the concentration of FMDV in the interstitial space. This saturation function enforces a delay to slow down the FMDV infection of susceptible cells when the concentration is very low for FMDV in the interstitial space (Howey et al. 2009). Such saturation effect in the infection process has not yet been reported or tested for measles in the literature. Though our modeling results support virus-to-cell infection for MV with a mass-action process, additional experimental and mathematical studies are required to improve our understanding of virus-to-cell and cell-to-cell infection for measles.

When the infection dose increases in the context of acute IBV infection in chickens, the magnitude of the peak viremia remains almost constant whist the initial growth rate, the time to peak, and the duration of infection decrease. modeling studies in Li and Handel (2014) showed that when the initial number of responding T cell is large, the observed infection dose responses could be described by ODEs where the antigen-specific T cell proliferation saturates with increases in the viral load, and T cell killing saturates with increases in the number of infected cells (Li and Handel 2014). Model-data fitting results estimated low initial numbers ($<100$) of activated MV-specific T cells in this work and in Morris et al. (2018). Additionally, the mass-action term and modeling results in this work and in Morris et al. (2018) produced relatively low numbers of infected and effector cells up to the peak viremia. Thus, MV infection dose responses are likely to be unrelated to saturation in T cell killing. In contrast with our results, the work in in Li and Handel (2014) shows that when T cell killing follows mass-action kinetics within a certain parameter space, the higher the infection dose, the higher the peak viremia. As expected, the parameter space may allow, or block a given ODE model to exhibit patterns of infection dose responses. Thus, the features of infection dose responses could be described by similar or different mechanisms and parameter values, to match particular phenomena in virus pathogenesis and immune responses. Together, current findings motivate close collaborations between mathematical and experimental studies to discriminate between different mechanisms, and advance understanding of MV interactions with immune cells such as antigen-presenting cells and precursor T cells.

## Supplementary information

The data and code are available online in the supplementary information, and upon request to the authors. The data is published in van Binnendijk et al. (1994).

### Supplementary Information

Below is the link to the electronic supplementary material.

Supplementary file 1 (csv 1 KB)

Supplementary file 2 (pdf 219 KB)

Supplementary file 3 (pdf 512 KB)

## Data Availability

Data and code are available online in the supplementary information, and upon request to the authors.

## Appendix A

### Area Under the Curve of Acute Viremia

We computed the area under the curve (AUC) to assess MV accumulation in PBMCs. We followed the method in (El Mubarak et al. 2007), and computed the AUC between days 0 and 18 days post MV infection, using the trapezoidal method of numerical integration, after natural logarithmic transformation of measurements of PBMC-associated viremia at or above the limit of detection. Next, we performed natural logarithmic transformation of the resultant AUC, to ease comparison with the AUC computed from similar MV experimental studies in El Mubarak et al. (2007), see Table 3.

**Table 3:** Area under the curve (AUC) for PBMC-associated acute viremia for different MV infection doses, data from van Binnendijk et al. (1994)

TCID_50_	$10^4$				
AUC	4.61	4.43	4.66	4.66	4.63

### Model Selection

We performed model selection using the Akaike Information Criterion corrected for small sample sizes (AICc) (Hurvich and Tsai 1989),

A1 $$\begin{aligned} AICc= & {} 2 \hat{k} - 2 ln \hat{L} + \frac{ 2 \hat{k} (\hat{k} + 1) }{ n- \hat{k} -1 }. \end{aligned}$$

where *n* is the number of data points used for fitting, $Ln \hat{L}_{1}$ is the maximum likelihood estimate (13), and $\hat{k}$ is the number of estimated parameters of the given model. We used the AICc to evaluate the goodness of fit whilst also penalizing models with more parameters. We used AICc values to identify the best-fitting model parameterization because a more parsimonious fit leads to a lower AICc.

### Computational Environment

We used Rstudio with R version 4.0.4. We solved ODEs numerically using the R package deSolve version 1.32; the relative and absolute tolerance of the solver was set to $10^-14$. We designed the figures using the R packages cowplot version 1.1.1, ggplot2 version 3.3.6, and gridExtra version 2.3.
