# Supplementary material for: Measles Infection Dose Responses: Insights from Mathematical Modeling
Source: Bull Math Biol. 2024 Jun 9;86(7):85. doi: 10.1007/s11538-024-01305-0 (PMC11162976; doi:10.1007/s11538-024-01305-0)
Supplement: Supplementary file 2 — (pdf 219 KB) [file 11538_2024_1305_MOESM2_ESM.pdf]

```
########################################################################################
#fitting data from IAV infections at different inoculum doses
#written by Andreas Handel (ahandel@uga.edu)
#last changed on 10/28/2017
#######################


cat(  "set workingdirectory HPC")
setwd("/home/svu/anelone")
"Linux config"


#################################################################
rm(list=ls()) #this clears the workspace to make sure no leftover variables are floating around. Not strictly needed
graphics.off(); #close all graphics windows
library(deSolve)  #loads ODE solver package
library(nloptr) #for fitting
 #for data prep
library(snow) #for parallel computing
library(matrixcalc)
library(rlang)
library(ggplot2)
library(gridExtra)


library(cowplot)
#https://mran.microsoft.com/snapshot/2017-03-04/web/packages/EGRET/vignettes/rResid.html
library(tgp)


#setwd("~/Anet_MEV_NUS/mv_rcode")



#  source('iavequations.R') #load function that contains ODE model
```

```
source('mvequations.R') #load mv model


#######################################################################
mvequations_di=function(t,y,parms)
{
   with(
      as.list(c(y,parms)), #lets us access variables and parameters stored in y and pars by name
      {

         # Activation of susceptible lymphocytes
          DL <- 0
         if ( t < td )  {
           DL <-  1
         } else {
           DL <- 0
         }

         #  susceptible cells
         dSdt =  - b*S* V + qs*DL*S + r*(1- ( V/ (s+ V )))*A



         # Infected cells
         dIdt =  b * ( S + A )*V   - k*I*A

         # Activated T cells
         dAdt = q* ( V/ (s+ V ))* A - b* A * V - ( 1- ( V/ (s+ V )))*(d+r) * A

         # Infectious virus particles
```

```
        dVdt = p * I - c* V

        list(c(dSdt, dIdt, dAdt, dVdt ))

      }
  ) #close with statement
} #end function withinode



######################################
# multistart
#number of multisart
set.seed(1)

NMS <- 50

NMS_seq  <-   seq(1, NMS )
NMS_seq
##############################################
#function that fits the ODE model to data
###########################

###########################



# Step 2: Derive the likelihood function




# Likelihood function
log_like <- function(theta ){
```

```
#one monkey at a time

# print(sprintf('Running LL Monkey ID = %f _ %s ',ii, mcqii ))
# times
# X <- as.matrix(X  );
loglik = NULL;
loglik_IV_10000  = NULL;
loglik_IV_1000  = NULL;
loglik_IV_100  = NULL;
loglik_IV_10  = NULL;
loglik_IV_1 = NULL;


#data
#   Y <- as.matrix( Y);

# times
#vector for integration times
t_tlym<- as.matrix( day_tlym );
t_ivl  <- as.matrix( day_ivl );
# counts of data
#  N       <- nrow(X)
n_times  <- length(times);
n_days_tlym  <- length( day_tlym );
n_days_ivl  <- length( day_ivl );
#1994




# Data
```

```
#data accummulation code
#  allvirus_data = c(allvirus_data, virusdata)

m_tlym  <- as.matrix(  mes_tlym  );
m_tcell  <- as.matrix(  mes_tcell );
m_ivl  <- as.matrix(  mes_ivl  );

#print(sprintf(' prep ode ' ))
# times
###prep  ode
#parameters
parsode = 10^(theta); #transform parameters back to exponential since we fit in log space

#  model Initial conditions
# efi <- tail( parsode,1)

A0_10000 <-    parsode[1];
A0_1000 <-  parsode[2];
A0_100 <-  parsode[3];
A0_10 <-  parsode[4];
A0_1 <-  parsode[5];

V_10000  <-  parsode[6];
V_1000  <-  parsode[7];
V_100  <-  parsode[8];
V_10  <-  parsode[9];
V_1  <-  parsode[10];
```

```
L0_10000 <-  3906 #parsode[11];




#names(theta)=parnames; #assign names to parameters
#print(sprintf(' prep ode para assign ' ))
#print(sprintf('A0 LL = %f  ',A0_10000 ))

#Define parameter value

allparsode <-   c( 0.028, #  parsode[ 12] ,
                 2.6 , #  parsode[13] ,
                  parsode[11] ,
                  0.5 ,
                  parsode[12] ,
                 1.11, #  parsode[12] ,
                  parsode[13] ,
                  0.025,
                 0.016, #  parsode[18] ,
                  parsode[14] ,
                  3 )

names(allparsode)=defparnames; #assign names to parameters

#  print(sprintf('td LL  = %f  ',allparsode[2] ))
# times
# RUN ODE
#print(sprintf(' start run ode ' ))

INC <- c(  L0_10000-A0_10000, 0, A0_10000,  V_10000 );
```

```
  names(INC)= namevar; #assign names to parameters

  odestack=NULL
  IVmlin = NULL
  #  odestack=try(ode( func = mvequations,  y = INC, times = t_al, parms=allparsode, method ="daspk")); #runs the ODE equations

  odestack=try(lsoda( INC, t_al,mvequations_di,parms=allparsode, atol=atolv,rtol=rtolv)); #runs the ODE equations
    if (length(odestack)==1) {cat('!!unresolvable integrator error - triggering early return from optimizer!!'); return(eturmerror) } #catching errors that might happen during fitting
    IVmlin =odestack[match( t_ivl_10 ,odestack[,1]),Vpos+1]; #extract values for virus load at time points corresponding to experimental measurements
  #plot( t_ivl_10,   m_ivl_10000)
    if (sum(is.na(IVmlin))>0) {cat('!!ODE 10000fail VL NA return!!'); return(eturmerror) } #catching errors that might happen during fitting
  if (sum( is.infinite(10^IVmlin))>0) {cat('!!ODE 10000fail VL INF correct!!');  return(eturmerror) } # IVmlin[ which( IVmlin>300  ) ] <-300 } #catching errors that might happen during fitting

  Smlin=odestack[match(t_al,odestack[,1]),Spos+1]; #extract values for virus load at time points corresponding to experimental measurements
  Imlin=odestack[match( t_al ,odestack[,1]),Ipos+1]; #extract values for virus load at time points corresponding to experimental measurements
  Amlin_10000=odestack[match(t_al ,odestack[,1]),Apos+1]; #extract values for virus load at time points corresponding to experimental measurements
  Lmscal_10000 = Smlin + Imlin + Amlin_10000
  #if (Lmscal_10000[length(Lmscal_10000)]< 2000) {cat('!!ODE 10000faIL LOW TLYM'); return(eturmerror) }

  IVmlong_10000 =odestack[match( t_al ,odestack[,1]),Vpos+1]; #extract values for virus load at time points corresponding to experimental measurements

  # correct lod
```

```
  if (IVmlin[1] <= 0.3) {IVmlin[1]  <- 0.3 }

  if (IVmlin[7] <= 0.3) {IVmlin[7]  <- 0.3 }

  if (IVmlin[8] <= 0.3) {IVmlin[8]  <- 0.3 }


  RSS_IV    <-   log( 10^IVmlin )  - log( 10^m_ivl_10000 )


  loglik_IV_10000 <-  -(n_days_ivl_10 /2)*  log(  (2*pi/n_days_ivl_10) * ( t( RSS_IV ) %*% RSS_IV )  ) - (n_days_ivl_10 /2)


  IVmlin_10000  <- IVmlin




  INC <- c(  L0_10000-A0_1000, 0, A0_1000,  V_1000 );

  names(INC)= namevar; #assign names to parameters

  odestack=NULL

  IVmlin = NULL



  #  odestack=try(ode( func = mvequations,  y = INC, times = t_al, parms=allparsode, method ="daspk")); #runs the ODE equations

  odestack=try(lsoda( INC, t_al,mvequations_di,parms=allparsode, atol=atolv,rtol=rtolv)); #runs the ODE equations

  if (length(odestack)==1) {cat('!!unresolvable integrator error - triggering early return from optimizer!!'); return(eturmerror) } #catching errors that might happen during fitting

  IVmlin =odestack[match( t_ivl_10 ,odestack[,1]),Vpos+1]; #extract values for virus load at time points corresponding to experimental measurements

  if (sum(is.na(IVmlin))>0) {cat('!!ODE 10000fail VL NA return!!'); return(eturmerror) } #catching errors that might happen during fitting

  if (sum( is.infinite(10^IVmlin))>0) {cat('!!ODE 10000fail VL INF correct!!'); return(eturmerror) } # IVmlin[ which( IVmlin>300  ) ] <-300 } #catching errors that might happen during fitting


  Smlin=odestack[match(t_al,odestack[,1]),Spos+1]; #extract values for virus load at time points corresponding to experimental measurements
```

```
  Imlin=odestack[match( t_al ,odestack[,1]),Ipos+1]; #extract values for virus load at time points corresponding to experimental measurements

  Amlin_1000 =odestack[match(t_al ,odestack[,1]),Apos+1]; #extract values for virus load at time points corresponding to experimental measurements

  Lmscal_1000 = Smlin + Imlin + Amlin_1000

  IVmlong_1000 =odestack[match( t_al ,odestack[,1]),Vpos+1]; #extract values for virus load at time points corresponding to experimental measurements


  # correct lod

  if (IVmlin[1] <= 0.3) {IVmlin[1]  <- 0.3 }

  if (IVmlin[2] <= 0.3) {IVmlin[2]  <- 0.3 }

  if (IVmlin[8] <= 0.3) {IVmlin[8]  <- 0.3 }


  RSS_IV     <-    log(  10^IVmlin )  - log(  10^m_ivl_1000 )

  loglik_IV_1000 <-  -(n_days_ivl_10 /2)*  log(  (2*pi/n_days_ivl_10) * ( t( RSS_IV ) %*% RSS_IV )  ) - (n_days_ivl_10 /2)


  IVmlin_1000  <- IVmlin



  INC <- c(  L0_10000-A0_100, 0, A0_100,  V_100 );


  names(INC)= namevar; #assign names to parameters

  odestack=NULL

  IVmlin = NULL



  #  odestack=try(ode( func = mvequations,  y = INC, times = t_al, parms=allparsode, method ="daspk")); #runs the ODE equations

  odestack=try(lsoda( INC, t_al,mvequations_di,parms=allparsode, atol=atolv,rtol=rtolv)); #runs the ODE equations

  if (length(odestack)==1) {cat('!!unresolvable integrator error - triggering early return from optimizer!!'); return(eturmerror) } #catching errors that might happen during fitting
```

```
  IVmlin =odestack[match( t_ivl_10 ,odestack[,1]),Vpos+1]; #extract values for virus load at time
points corresponding to experimental measurements

  if (sum(is.na(IVmlin))>0) {cat('!!ODE 10000fail VL NA return!!'); return(eturmerror) } #catching
errors that might happen during fitting

  if (sum( is.infinite(10^IVmlin))>0) {cat('!!ODE 10000fail VL INF correct!!'); return(eturmerror) } #
IVmlin[ which( IVmlin>300  ) ] <-300 } #catching errors that might happen during fitting


  Smlin=odestack[match(t_al,odestack[,1]),Spos+1]; #extract values for virus load at time points
corresponding to experimental measurements

  Imlin=odestack[match( t_al ,odestack[,1]),Ipos+1]; #extract values for virus load at time points
corresponding to experimental measurements

  Amlin_100=odestack[match(t_al ,odestack[,1]),Apos+1]; #extract values for virus load at time points
corresponding to experimental measurements

  Lmscal_100 = Smlin + Imlin + Amlin_100


  IVmlong_100 =odestack[match( t_al ,odestack[,1]),Vpos+1]; #extract values for virus load at time
points corresponding to experimental measurements


  # correct lod

  if (IVmlin[1] <= 0.3) {IVmlin[1]  <- 0.3 }

  if (IVmlin[2] <= 0.3) {IVmlin[2]  <- 0.3 }

  if (IVmlin[8] <= 0.3) {IVmlin[8]  <- 0.3 }


  RSS_IV     <-    log(  10^IVmlin )  - log(  10^m_ivl_100 )

  loglik_IV_100 <-  -(n_days_ivl_10 /2)*  log(  (2*pi/n_days_ivl_10) * ( t( RSS_IV ) %*% RSS_IV )  ) -
(n_days_ivl_10 /2)


  IVmlin_100  <- IVmlin


  INC <- c(  L0_10000-A0_10, 0, A0_10,  V_10 );


  names(INC)= namevar; #assign names to parameters

  odestack=NULL
```

```
  IVmlin = NULL



  #  odestack=try(ode( func = mvequations,  y = INC, times = t_al, parms=allparsode, method ="daspk")); #runs the ODE equations

  odestack=try(lsoda( INC, t_al,mvequations_di,parms=allparsode, atol=atolv,rtol=rtolv)); #runs the ODE equations

  if (length(odestack)==1) {cat('!!unresolvable integrator error - triggering early return from optimizer!!'); return(eturmerror) } #catching errors that might happen during fitting

  IVmlin =odestack[match( t_ivl_10 ,odestack[,1]),Vpos+1]; #extract values for virus load at time points corresponding to experimental measurements

  if (sum(is.na(IVmlin))>0) {cat('!!ODE 10000fail VL NA return!!'); return(eturmerror) } #catching errors that might happen during fitting

  if (sum( is.infinite(10^IVmlin))>0) {cat('!!ODE 10000fail VL INF correct!!');  return(eturmerror) } # IVmlin[ which( IVmlin>300  ) ] <-300 } #catching errors that might happen during fitting


  Smlin=odestack[match(t_al,odestack[,1]),Spos+1]; #extract values for virus load at time points corresponding to experimental measurements

  Imlin=odestack[match( t_al ,odestack[,1]),Ipos+1]; #extract values for virus load at time points corresponding to experimental measurements

  Amlin_10 =odestack[match(t_al ,odestack[,1]),Apos+1]; #extract values for virus load at time points corresponding to experimental measurements

  Lmscal_10 = Smlin + Imlin + Amlin_10


  IVmlong_10 =odestack[match( t_al ,odestack[,1]),Vpos+1]; #extract values for virus load at time points corresponding to experimental measurements


  # correct lod

  if (IVmlin[1] <= 0.3) {IVmlin[1]  <- 0.3 }

  if (IVmlin[2] <= 0.3) {IVmlin[2]  <- 0.3 }

  if (IVmlin[8] <= 0.3) {IVmlin[8]  <- 0.3 }


  RSS_IV     <-    log(  10^IVmlin )  - log(  10^m_ivl_10 )

  loglik_IV_10 <-  -(n_days_ivl_10 /2)*  log(  (2*pi/n_days_ivl_10) * ( t( RSS_IV ) %*% RSS_IV )  ) - (n_days_ivl_10 /2)
```

```
  IVmlin_10  <- IVmlin



  INC <- c(  L0_10000-A0_1, 0, A0_1,  V_1 );

  names(INC)= namevar; #assign names to parameters
  odestack=NULL
  IVmlin = NULL


  #  odestack=try(ode( func = mvequations,  y = INC, times = t_al, parms=allparsode, method ="daspk")); #runs the ODE equations
  odestack=try(lsoda( INC, t_al,mvequations_di,parms=allparsode, atol=atolv,rtol=rtolv)); #runs the ODE equations
  if (length(odestack)==1) {cat('!!unresolvable integrator error - triggering early return from optimizer!!'); return(eturmerror) } #catching errors that might happen during fitting
  IVmlin =odestack[match( t_ivl_1 ,odestack[,1]),Vpos+1]; #extract values for virus load at time points corresponding to experimental measurements
  if (sum(is.na(IVmlin))>0) {cat('!!ODE 10000fail VL NA return!!'); return(eturmerror) } #catching errors that might happen during fitting
  if (sum( is.infinite(10^IVmlin))>0) {cat('!!ODE 10000fail VL INF correct!!'); return(eturmerror) } # IVmlin[ which( IVmlin>300  ) ] <-300 } #catching errors that might happen during fitting

  Smlin=odestack[match(t_al,odestack[,1]),Spos+1]; #extract values for virus load at time points corresponding to experimental measurements
  Imlin=odestack[match( t_al ,odestack[,1]),Ipos+1]; #extract values for virus load at time points corresponding to experimental measurements
  Amlin_1 =odestack[match(t_al ,odestack[,1]),Apos+1]; #extract values for virus load at time points corresponding to experimental measurements
  Lmscal_1 = Smlin + Imlin + Amlin_1
```

```
  IVmlong_1 =odestack[match( t_al ,odestack[,1]),Vpos+1]; #extract values for virus load at time
points corresponding to experimental measurements


  # correct lod
  if (IVmlin[1] <= 0.3) {IVmlin[1]  <- 0.3 }
  if (IVmlin[2] <= 0.3) {IVmlin[2]  <- 0.3 }
  if (IVmlin[3] <= 0.3) {IVmlin[3]  <- 0.3 }


  RSS_IV     <-    log(  10^IVmlin )  - log(  10^m_ivl_1 )
  loglik_IV_1 <-  -(n_days_ivl_1 /2)*  log(  (2*pi/n_days_ivl_1) * ( t( RSS_IV ) %*% RSS_IV )  ) -
(n_days_ivl_1 /2)


  IVmlin_1  <- IVmlin


  odestack=NULL
  IVmlin = NULL


  loglik <-  loglik_IV_10000  +  loglik_IV_1000  +loglik_IV_100  + loglik_IV_10  +  loglik_IV_1
  #print(sprintf(' end -loglik ' ))
  #print(sprintf(' end -loglik ' ))


  if (is.na(loglik))  {cat('!!na loglik!!'); return(eturmerror) }


  return(-loglik)
}
##########################################
##############################################
##############################################
#####################################################
#wrapper function that runs parallel over different methods
##############################################################
outerfitfc <- function(NMS_seq  )
```

```
{

  fitAICC =NULL;
  MLE_estimates =NULL;
  para_guess =NULL;


  #selection of one start

  para_guess = log10(   (para_guess_MS[,NMS_seq ]))


  #error check
  #if (min(logub-para_guess)<0 | min(para_guess-loglb)<0)
  #{ print(sprintf('initial condition out of bound')); para_guess=pmax(pmin(logub,para_guess),loglb);
}

  names(para_guess)=parnames; #assign names to parameters
  #para_guess


  MLE_estimates <- optim(fn=log_like,                  # Likelihood function
                      #par= log10(MLE_estimates$par),                  # Initial guess
                      par= para_guess,                   # Initial guess
                      lower = loglb,        # Lower bound on parameters
                      upper = logub,          # Upper bound on parameters
                      hessian=TRUE,
                      #method = "BFGS",
                      method = "L-BFGS-B",
                      control = list(maxit=10^9,   reltol= 1e-20,  factr = 1e-20))
```

```
MLE_par <-  10^(MLE_estimates$par)


if (as.numeric(MLE_estimates$value) == eturmerror) {
  cat('!!FULL EARLY RETURN OPTIM!');
  MLE_estimates$value = - MLE_estimates$value;
  MLE_SE <-  MLE_par *0
   #return(resvector)
} else {
  if ( is.singular.matrix(as.matrix(MLE_estimates$hessian), tol = 1e-08) )
  {   MLE_SE <-  MLE_par *0
  } else {
    MLE_SE <-sqrt(diag(solve(MLE_estimates$hessian)));
  }
}
fitAICC = 2* knep -2* (-MLE_estimates$value) + (2*knep*(knep+1) )/( ndatafitted - knep -1)


print(sprintf('MScur  = %f , loglik = %f , AICC= %f  ', NMS_seq, -MLE_estimates$value, fitAICC  ))



#if (-MLE_estimates$value > temploglik ) {
 # temploglik <- -MLE_estimates$value
#  flagms  <- NMS_seq
#}


# Standard error
#


resvector=c(flagms,NMS_seq, MLE_estimates$convergence, -
MLE_estimates$value,fitAICC,MLE_SE,MLE_par)
```

```
  names(resvector) <-  matnames

  # if ( -MLE_estimates$value > temploglik ) {
  #  temploglik <- -MLE_estimates$value
  #  fitAICC

  #save( fitAICC, file = 
paste("~/Anet_MEV_NUS/mv_rcode/mlepat_mevac_var_1994_QSdI_ALLP_tempaicc.RData", 
sep=""))
  # save(MLE_estimates, file = 
paste("~/Anet_MEV_NUS/mv_rcode/mlepat_mevac_var_1994_QSdI_ALLP_full.RData", sep=""))
  #}

  ###################
  #check if we alrea
  return(resvector) #return results from optimizer to main function

} #finish function for each scenario

##################################
#main progr
##################################
#main program
##################################
#tstart=proc.time(); #capture current time to measure duration of process


#%%%%%%%%%%%%%%%%%%%%%%%%%%%%%%%
#data from Ginsberg 1952
#virus and lung damage sampled at different times
#position/index for the different variables
#The palette with black:
```

```
cbbPalette <- c( "#E69F00", "#009E73",  "#0072B2", "#D55E00", "#CC79A7")
# To use for fills, add

# To use for line and point colors, add
#scale_colour_manual(values=cbPalette)

temploglik <-  -Inf
flagms  <- 0

namevar = c('S','I','A','V')
# Initial conditions from the previous model fit
iS0 <-   c( 3828,  3905, 2456, 4053,   5487,  3342,   3876 )
iI0 <-   c( 0, 0, 0, 0, 0 , 0, 0)
iA0 <-   c(  36.5,  1.1, 1.2, 74.7,  2.6,67.4, 3.8 )
iV0 <-   c(  1.1*10^-5 ,  2.5*10^-5,1.0*10^-5, 1.0*10^-6, 9.9*10^-5, 3.0*10^-6,  1.0*10^-4 )


mcnames  <- c(  '15U','46U','55U','67U','40V','43V','55V')

#  Biological rates of each macaques
#Read csv file containing theestimarted parameter values from th previous work
estparamvac <- read.csv("estparamvac.csv", header = TRUE, sep = ",")
# view and check data
estparamvac



cat("red data from mvdata csv")
mesacmv <- read.csv("mvdata.csv", header = TRUE, sep = ",")
"show data from csv"
mesacmv
```

```
unique(mesacmv$ID)


min(mesacmv$MV.specific.T.cells )


mesacmv$IDM <- (mesacmv$ID)
mesacmv$IDM <-0
mesacmv$IDM <- as.numeric(mesacmv$IDM)
"start looping mc id"


for (ni in 1:7) {
  "befoe which output"
  mesacmv$ID== mcnames[ni]
  "which output"
  which(mesacmv$ID== mcnames[ni] )
  selidmw <- which(mesacmv$ID== mcnames[ni] )
  selidmw <-as.numeric(selidmw)
  "numeric selidmw"
  selidmw
  "before"
  mesacmv$IDM[selidmw]
  mesacmv$IDM[selidmw] <- ni
  "after"
  mesacmv$IDM[selidmw]
}
mesacmv$IDM <- as.numeric(mesacmv$IDM)
"create numerical newID for monkeys"
mesacmv$IDM


########################################
```

```
cat("red data from mevrnacsv")
mevrnadata <- read.csv("mevrnadatalin12.csv", header = TRUE, sep = ",")
"show data from mevrnadata csv"
mevrnadata



mevrnadata$IDM <- mevrnadata$ID
mevrnadata$IDM <-0
mevrnadata$IDM <- as.numeric(mevrnadata$IDM)
"start looping mc id"

for (ni in 1:7) {
  "befoe which output"
  mevrnadata$ID== mcnames[ni]
  "which output"
  which(mevrnadata$ID== mcnames[ni] )
  selidmw <- which(mevrnadata$ID== mcnames[ni] )
  selidmw <-as.numeric(selidmw)
  "numeric selidmw"
  selidmw
  "before"
  mevrnadata$IDM[selidmw]
  mevrnadata$IDM[selidmw] <- ni
  "after"

}
mevrnadata$IDM <- as.numeric(mevrnadata$IDM)
"create numerical newID for monkeys"
mevrnadata$IDM
```

```
###########################################

# ivlmev1994


cat("red data from ivlmev1994 csv")

ivlmev <- read.csv("ivlmev1994.csv", header = TRUE, sep = ",")

ivlmev

EID <- unique(ivlmev$eid)


for ( itc in 1: length(EID) ) {


  assign(paste0("fmes_ivl_", EID[itc]),  ivlmev$ivl[ which(ivlmev$eid == EID[itc] & ivlmev$cens == 0  )
])

  assign(paste0("mes_day_", EID[itc]),  ivlmev$day[ which(ivlmev$eid == EID[itc] & ivlmev$cens == 0
& ivlmev$day >= 0 ) ])

  assign(paste0("mes_lod_", EID[itc]),  ivlmev$lod[ which(ivlmev$eid == EID[itc] & ivlmev$cens == 0 &
ivlmev$day >= 0  ) ])

  assign(paste0("mes_ivl_0_", EID[itc]),  ivlmev$ivl[ which(ivlmev$eid == EID[itc] & ivlmev$cens == 0
& ivlmev$day == 0  ) ])

  assign(paste0("mes_ivl_", EID[itc]),  ivlmev$ivl[ which(ivlmev$eid == EID[itc] & ivlmev$cens == 0 &
ivlmev$day >= 0  ) ])


}


mes_day_1

n_days_ivl_1 <- length(mes_day_1)

n_days_ivl_10 <- length(mes_day_10)


#ivlmevsub <- subset (ivlmev, ivlmev$eid <=1000 & ivlmev$cens == 0)

#ivlmevsub0 <- subset (ivlmev, ivlmev$eid <=1000 & ivlmev$cens == 0  & ivlmev$day > 0)


ivlmevsub <- subset (ivlmev, ivlmev$eid != 1001 &ivlmev$eid !=1002 & ivlmev$cens == 0)

ivlmevsub0 <- subset (ivlmev, ivlmev$eid != 1001 & ivlmev$eid !=1002 & ivlmev$cens == 0  &
ivlmev$day > 0)
```

```
#

cat(  "Data 1994")

data94 <- data.frame(  TCID = c( mes_day_10*0+10^4,   mes_day_10*0+10^3,mes_day_10*0+10^2,
mes_day_10*0+10^1, mes_day_1*0+10^0)  ,
                    mes =  c( mes_ivl_10000, mes_ivl_1000, mes_ivl_100, mes_ivl_10, mes_ivl_1  ) ,
                    t =   c(  mes_day_10, mes_day_10, mes_day_10, mes_day_10,   mes_day_1 ) )

data94

cat("ndatafitted ")

ndatafitted <-  length(mes_day_1)+ 4*length(mes_day_10)
ndatafitted

t_ivl_1  <- as.matrix( mes_day_1 );
t_ivl_10  <- as.matrix( mes_day_10 );

cat("t_ivl_1 t_ivl_10 ")
t_ivl_1

t_ivl_10

##############
n_days_ivl_1 <- length(t_ivl_1)
```

```
n_days_ivl_10 <- length(t_ivl_10)


m_ivl_10000  <- as.matrix(  mes_ivl_10000  );
m_lod_10000  <- as.matrix(  mes_lod_10000  );
m_ivl_1000  <- as.matrix(  mes_ivl_1000  );
m_lod_1000  <- as.matrix(  mes_lod_1000  );
m_ivl_100  <- as.matrix(  mes_ivl_100  );
m_lod_100  <- as.matrix(  mes_lod_100  );
m_ivl_10  <- as.matrix(  mes_ivl_10  );
m_lod_10  <- as.matrix(  mes_lod_10  );
m_ivl_1  <- as.matrix(  mes_ivl_1  );
m_lod_1  <- as.matrix(  mes_lod_1  );

####################################
#  parnames=c('A_10000','V0_10000','V0_1000','V0_100','V0_10','V0_1','q','s')

#parnames=c('A0','V0_10000','qs','td','b','k','q','s','r','p','fi' )

defparnames = c('qs','td','b','di','k','q','s','d','r','p','c' )

# parmdl=c('qs','td','b','k','q','s','r','p','fi' )

Spos=1; Ipos=2; Apos=3; Vpos=4;

eturmerror <- 10^100

atolv=1e-14; rtolv=1e-14; #tolerances for ODE solver

n_monkeys = 7
eps=1e-15;# epsilon for log
```

```
#####################################################



####################
########################################

#pdf(paste("~/Anet_MEV_NUS/mv_rcode/testMLEincoculum".pdf", sep=""),, paper="a4r")

#par(mfrow=c(3,1), oma=c(1,1,1), mar=c(2,1,1))




#bounds on initial conditions
#bounds for parameters improves solver convergence
IV_10000_low=10^-16 ; IV_10000_high= 0.8; #for V0
IV_1000_low=1e-16; IV_1000_high=  0.3; #
IV_100_low=1e-16; IV_100_high=  0.3 ; #
IV_10_low=1e-16; IV_10_high= 0.3; #
IV_1_low=1e-16; IV_1_high= 0.3   ; #
IV_01_low=1e-16; IV_01_high=0.3     ; #

L_10000_low=3906 ; L_10000_high= 3907; #for L0
S0_low=1 ; S0_high=10^7; #for S0

R_low=1e-10; R_high=10^6; #for rna

A_10000_low=1 ;   A_10000_high= 200; #for V0
A_1000_low=1 ;   A_1000_high= 200 ; #for V0
```

```
A_100_low=1 ;   A_100_high=200 ; #for V0

A_10_low=1 ;   A_10_high= 200 ; #for V0

A_1_low=1 ;   A_1_high= 200 ; #for V0



q_low= 1.11 ; q_high= 1.119 ;

s_low= 0.0001 ; s_high= 0.25 ;


#bounds on parameters, all is in units o


qs_low= 0.028 ; qs_high= 0.029 ;

td_low=  2.6 ; td_high=2.7;


b_low= 0.01 ; b_high= 1 ;


di_low= 0.5 ; di_high=0.5;


k_low= 0.0007 ; k_high=0.05;


d_low= 1/40 ; d_high= 1/40  ;

r_low= 0.016 ; r_high= 0.017 ;

p_low=0.001; p_high= 0.025 ;

c_low= 3 ; c_high= 3 ;

fi_low=100; fi_high=10000;




#vector of parameter names and lower/upper bounds

# parnames=c('A0','V0_10000','qs','td','fi' )


# parnames=c('td','b','k','q','s','r','p','fi' )
```

```
#parnames=c('A_10000','A_1000','A_100','A_10','A_1','V0_10000','V0_1000','V0_100','V0_10','V0_1'
,'qs','td','b','k','q','s','r','p','fi' )

#lb=c(A0_low, IV_10000_low,  qs_low, td_low, fi_low)

# ub = c (A0_high, IV_10000_high,  qs_high , td_high, fi_high)

lb=c( A_10000_low,A_1000_low, A_100_low, A_10_low, A_1_low,
IV_10000_low,IV_1000_low,IV_100_low,IV_10_low,IV_1_low, b_low,k_low,  s_low, p_low );

ub = c(A_10000_high,A_1000_high,A_100_high,A_10_high,A_1_high,
IV_10000_high,IV_1000_high,IV_100_high,IV_10_high,IV_1_high, b_high,k_high,  s_high, p_high);

parnames=c('A_10000','A_1000','A_100','A_10','A_1','V0_10000','V0_1000','V0_100','V0_10','V0_1','
b','k','s','p')

parnames_se =
c('se_A_10000','se_A_1000','se_A_100','se_A_10','se_A_1','se_V0_10000','se_V0_1000','se_V0_100'
,'se_V0_10','se_V0_1','se_b','se_k','se_s','se_p')

rect_lbub <- cbind(lb, ub)

length(parnames )

length(rect_lbub)/2

length(lb )

######################################
```

```
# multistart
#number of multisart



real.time.start=date(); #get current time to measure length of optimization for each strain
tstart=proc.time(); #capture current time
print(sprintf('Optimization started at %s ',real.time.start))

############### unbiformn multistart
IV_10000_ug <-   seq(IV_10000_low, IV_10000_high, length=NMS )
IV_1000_ug <-   seq(IV_1000_low, IV_1000_high, length=NMS )
IV_100_ug <-   seq(IV_100_low, IV_100_high, length=NMS )
IV_10_ug <-   seq(IV_10_low, IV_10_high, length=NMS )
IV_1_ug <-   seq(IV_1_low, IV_1_high, length=NMS )
IV_01_ug <-   seq(IV_01_low, IV_01_high, length=NMS )

L_10000_ug <-   seq(L_10000_low, L_10000_high, length=NMS )
A_10000_ug <-   seq(A_10000_low, A_10000_high, length=NMS )
A_1000_ug <-   seq(A_1000_low, A_1000_high, length=NMS )
A_100_ug <-   seq(A_100_low, A_100_high, length=NMS )
A_10_ug <-   seq(A_10_low, A_10_high, length=NMS )
A_1_ug <-   seq(A_1_low, A_1_high, length=NMS )

R_ug <-   seq(R_low, R_high, length=NMS )

qs_ug <-   seq(qs_low, qs_high, length=NMS )
td_ug <-   seq(td_low, td_high, length=NMS )
b_ug <-   seq(b_low, b_high, length=NMS )
k_ug <-   seq(k_low, k_high, length=NMS )
q_ug <-   seq(q_low, q_high, length=NMS )
```

```
s_ug <-   seq(qs_low, qs_high, length=NMS )

r_ug <-   seq(r_low, r_high, length=NMS )

p_ug <-   seq(p_low, p_high, length=NMS )

fi_ug <-   seq(fi_low, fi_high, length=NMS )



para_guess_ug <-  rbind(   A_1000_ug,  A_100_ug,  A_10_ug,  A_1_ug,
IV_10000_ug,IV_1000_ug,IV_100_ug,IV_10_ug,IV_1_ug,b_ug,k_ug, q_ug, s_ug,   p_ug);


para_guess_ug <- unname(para_guess_ug)




#####################################################

#######random sampling from uniform


IV_10000_rug <-   runif(NMS, min = IV_10000_low, IV_10000_high )

IV_1000_rug <-    runif(NMS, min = IV_1000_low, IV_1000_high )

IV_100_rug <-    runif(NMS, min = IV_100_low, IV_100_high )

IV_10_rug <-    runif(NMS, min =  IV_10_low, IV_10_high )

IV_1_rug <-    runif(NMS, min = IV_1_low, IV_1_high )

IV_01_rug <-    runif(NMS, min = IV_01_low, IV_01_high )


L_10000_rug <-    runif(NMS, min =  L_10000_low, L_10000_high )

A_10000_rug <-    runif(NMS, min =  A_10000_low, A_10000_high)

A_1000_rug <-    runif(NMS, min =  A_1000_low, A_1000_high)

A_100_rug <-    runif(NMS, min =  A_100_low, A_100_high)

A_10_rug <-    runif(NMS, min =  A_10_low, A_10_high)

A_1_rug <-    runif(NMS, min =  A_1_low, A_1_high)


R_rug <-    runif(NMS, min = R_low, R_high )
```

```
qs_rug <-    runif(NMS, min = qs_low, qs_high )

td_rug <-    runif(NMS, min = td_low, td_high )

b_rug <-    runif(NMS, min = b_low, b_high )

k_rug <-    runif(NMS, min = k_low, k_high )

q_rug <-    runif(NMS, min = q_low, q_high )

s_rug <-  runif(NMS, min = qs_low, qs_high )

r_rug <-  runif(NMS, min = r_low, r_high )

p_rug <-  runif(NMS, min = p_low, p_high )

fi_rug <-    runif(NMS, min =  fi_low, fi_high )



para_guess_rug <-    rbind( A_10000_rug,  A_1000_rug,  A_100_rug,  A_10_rug,  A_1_rug,
IV_10000_rug,IV_1000_rug,IV_100_rug,IV_10_rug,IV_1_rug,b_rug,k_rug,  s_rug, p_rug);



para_guess_rug <- unname(para_guess_rug)



###########

###### LHS latin hypercube


para_guess_lhs <- lhs(NMS, rect_lbub)

para_guess_lhs[1,]


para_guess_lhs <-  t(para_guess_lhs )


##########################################################

##########################################


#looping for each  of the 7 macaqies

ii <-  2
```

```
##################################################
# for ( ii in 1:7){
"ii"
ii
"mes day pi"
mesacmv$Day

mcqii <- unique(mesacmv$ID[ which(mesacmv$IDM== ii)])
mcqii

day_tlym <- mesacmv$Day[ which(mesacmv$IDM== ii & !is.na(mesacmv$Total.lymphocytes)  ) ]

enddpo <- max(mesacmv$Day)+ 1
times   <- seq(0, enddpo, by = 1)
t_al <- as.matrix(times );


"mes day pi day_tlym"
day_tlym


mes_tlym <- mesacmv$Total.lymphocytes[ which(mesacmv$IDM== ii &
!is.na(mesacmv$Total.lymphocytes)  ) ]

"mes pi mes_tlym"
mes_tlym


"length mes_tlym"
length(mes_tlym)
```

```
mes_tcell <- mesacmv$MV.specific.T.cells[ which(mesacmv$IDM== ii &
!is.na(mesacmv$MV.specific.T.cells )  ) ]

mes_AB <- mesacmv$MV.specific.antibodies[ which(mesacmv$IDM== ii &
!is.na(mesacmv$MV.specific.antibodies)  ) ]

day_ivl <- mesacmv$Day[ which(mesacmv$IDM== ii & !is.na(mesacmv$Viral.load)  ) ]

idmonkeydata <- subset(mesacmv, mesacmv$IDM== ii )

idxday_ivl <- which( !is.na(idmonkeydata$Viral.load)  )

mes_ivl <- mesacmv$Viral.load[ which(mesacmv$IDM== ii & !is.na(mesacmv$Viral.load)  ) ]

mes_tlym0 <-  mes_tlym[1]
fmes_tlym <-  mes_tlym
mes_tlym <-  mes_tlym
mes_tcell0 <-  mes_tcell[1]
fmes_tcell <-  mes_tcell
#log 0 issues
#reference
# https://aosmith.rbind.io/2018/09/19/the-log-0-
problem/#:~:text=The%20log%20transformation%20tends%20to%20feature%20prominently%20for,
This%20isn%E2%80%99t%20necessarily%20an%20incorrect%20thing%20to%20do.

HMA <-  min(fmes_tcell[fmes_tcell>0])/2
mes_tcell <-  mes_tcell
```

```
simday_tlym <- day_tlym

day_tlym <- day_tlym

n_days_tlym <- length(day_tlym)

(n_days_tlym)

length(day_tlym)

length(mes_tlym)

length(mes_tcell)



mes_ivl0 <-  mes_ivl[1]

fmes_ivl <-  mes_ivl

HMV <-  min(fmes_ivl[fmes_ivl>0])/2

mes_ivl <-  mes_ivl

day_ivl <- day_ivl

n_days_ivl <- length(mes_ivl)

length(n_days_ivl)

length(day_ivl)

length(mes_ivl)


cbdata_mes <-  append ( mes_tlym, mes_tcell )


cbdata_mes <-  append ( cbdata_mes, mes_ivl )



################################################


# Initial conditions



yicA =iA0[ii]
```

```
#Define parameter values

resvec  <-   c( qs = estparamvac$qs[ii] ,

             td =  estparamvac$td[ii] ,

             b  = estparamvac$b[ii],

             di  = estparamvac$di[ii],

             k = estparamvac$k[ii],

             q  = estparamvac$q[ii],

             s  = estparamvac$s[ii],

             d  = estparamvac$d[ii],

             r  = estparamvac$r[ii],

             p  = estparamvac$p[ii],

             c  = estparamvac$c[ii],

             fi  = estparamvac$fi[ii]  )


# RUN ODE


# ####################

yic    = c( S =iS0[ii], I = iI0[ii], A =iA0[ii], V =iV0[ii]   )

"Initialcondition"

yic

#   odestack=try(ode( func = mvequations,  y = yic, times = times, parms=resvec, method ="daspk"));
#runs the ODE equations


odestack=try(lsoda( yic, times, mvequations_di,parms=resvec, atol=atolv,rtol=rtolv)); #runs the ODE
equations

if (length(odestack)==1) {cat('!!unresolvable integrator error - triggering early return from
optimizer!!'); return(1e10) } #catching errors that might happen during fitting

print(sprintf(' end run ode out' ))



Smlin=odestack[match(mes_day_10,odestack[,1]),Spos+1]; #extract values for virus load at time
points corresponding to experimental measurements
```

```
Imlin=odestack[match( mes_day_10,odestack[,1]),Ipos+1]; #extract values for virus load at time
points corresponding to experimental measurements

Amlin=odestack[match(mes_day_10 ,odestack[,1]),Apos+1]; #extract values for virus load at time
points corresponding to experimental measurements


#   print(sprintf(' prep loglik ' ))


simes_tlym  = (Smlin + Imlin + Amlin)

simes_tcell  =  Amlin / estparamvac$fi[ii]

simes_ivl  =  odestack[match( mes_day_10,odestack[,1]),Vpos+1]; #extract values for virus load at
time points corresponding to experimental measurements


omes_tlym  <- mes_tlym

omes_tcell  <- mes_tcell

omes_ivl  <- mes_ivl_10000


mdata <- data.frame(  mtlym = omes_tlym ,
                mtcell = omes_tcell*1000  ,
                tm = day_tlym )




####################
####################################################


loglb=log10(lb); logub=log10(ub); #fit in log space


# initial guess


para_guess_mb = (lb+ub)/2


#para_guess_MS =   (para_guess_ug)
```

```
para_guess_MS =   (para_guess_rug)
 #para_guess_MS =   (para_guess_lhs)

knep <-  length(para_guess_MS[,1])
knep
length(para_guess_rug)

length(loglb)
length(logub)
length(parnames)

#selection of one start
para_guess_MS[,1]


##############################################################
#para_guess = (   (para_guess_MS[,1 ]))

#para_guess =   c(   1,
#                 0.3,
#                0.1,
#               0.1,
#              0.05,
#             iS0[ii],
#            1.307430e-02,
#           4.2,
#          0.05 ,
#         0.01 ,
#        0.5  ,
#       0.5,
#      0.1 ,
```

```
#      0.01  )

# Number of estimated parameters
#knep <-  length(para_guess)
#knep
#para_guess = log10(   (para_guess))

#names(para_guess)=parnames; #assign names to parameters

#MLE_estimates <- optim(fn=log_like,                  # Likelihood function
 #                    #par= log10(MLE_estimates$par),                  # Initial guess
  #                  par= para_guess,                  # Initial guess
   #                lower = loglb,        # Lower bound on parameters
    #              upper = logub,          # Upper bound on parameters
     #            hessian=TRUE,
      #          #method = "BFGS",
       #        method = "L-BFGS-B")
        #      #control = list(   reltol= 1e-20, lmm=31,  factr = 1e-20))
#

####################
#####################
##################################################
#matrix containing all results
length(parnames_se)+length(parnames)

matnames=c('FLAGLL','MS','CONVG','LOGLIK','AICC',parnames_se,parnames)
resmatrix=matrix(0,nrow=NMS,ncol=length(matnames))
colnames(resmatrix) <- matnames;
# resmatrix
```

```
reslist <- NULL
parallel.comp = 1 ; #turn on or off parallel computing -
node.num=NMS; #number of sockets/nodes to use for parallel computing
node.type=1; #choose socket/node type. 1 for SOCK (can be run locally), 2 for MPI


cat(  "start par optim")

if (parallel.comp==0) #standard non-parallel run using 1 core
{
  node.num=1; #just for printing purposes below
  node.type=1;
  reslist <- lapply(NMS_seq , outerfitfc)   #reslist contains best fits for all strains and all models
}
if (parallel.comp==1) #using snow package to do parallel computing
{
  if (node.type==1) {clust <- makeCluster(node.num, type = "SOCK")} #for local machines
  if (node.type==2) {clust <- makeCluster(node.num, type = "MPI")} #if run on a cluster
  clusterExport(clust,ls()) #make global variables available on each node/slave
  clusterEvalQ(clust, library(nloptr)) #load packages on each node
  clusterEvalQ(clust, library(deSolve))
  #clusterEvalQ(clust, library(dplyr))
  clusterEvalQ(clust, library(matrixcalc))
  reslist <- clusterApplyLB(clust, NMS_seq , outerfitfc)
  stopCluster(clust)
}


cat(  "end optim")
```

```
###########################################

real.time.stop=date();
tend=proc.time(); #capture current time
tdiff=tend-tstart;
runtime.minutes=tdiff[[3]]/60;

#print(resmatrix)
if (node.type==2) {mpi.quit() }

print(sprintf('Optimization ended at %s and took %f minutes using %d sockets with solver type
%d',real.time.stop,tdiff[[3]]/60,node.num,NMS_seq ));

##################
#reslist



ct=1;
for (nn in 1:(NMS))
{
  ind=match(names(reslist[[ct]]),names(resmatrix[nn,]));
  resmatrix[nn,ind]=reslist[[ct]]
  ct=ct+1;
}
#resmatrix

#take fit with highest loglikelihood (best fit) and process

cat("best fit all loglik ")
resmatrix[,"LOGLIK"]
maxind = which.max(resmatrix[,"LOGLIK"])
```

```
minind = which.min(resmatrix[,"AICC"])

minind

maxind


cat("best fit mle ")

resmatrix[maxind,]


MSLL_resmatrix = resmatrix[maxind,];


##save estimated  parameters

save(MSLL_resmatrix, file = paste("mle_saveparametrsL.RData", sep=""))


## save(MSLL_resmatrix, file = paste("mle_MShpc_1994FXICdi_res_46u_050_eeflo8xxLL.RData",
sep=""))


cat("optim duration")


runtime.minutes/60


############# PLots

##########################################################


A0_10000 <-   MSLL_resmatrix["A_10000"];

A0_1000 <-   MSLL_resmatrix["A_1000"];

A0_100 <-   MSLL_resmatrix["A_100"];

A0_10 <-   MSLL_resmatrix["A_10"];

A0_1 <-   MSLL_resmatrix["A_1"];




V_10000  <- MSLL_resmatrix["V0_10000"];

V_1000  <-   MSLL_resmatrix["V0_1000"];
```

```
V_100  <-   MSLL_resmatrix["V0_100"];
V_10  <-   MSLL_resmatrix["V0_10"];
V_1  <-   MSLL_resmatrix["V0_1"];

L0_10000 <-   3906# MSLL_resmatrix["L_10000"];

#Define parameter value

allparsode <-   c( 0.028, # MSLL_resmatrix["qs"] ,
                 2.6 , #MSLL_resmatrix["td"],
                 MSLL_resmatrix["b"] ,
                 0.5 ,
                 MSLL_resmatrix["k"],
                1.11, # MSLL_resmatrix["q"],
                 MSLL_resmatrix["s"],
                 0.025,
               0.016, #  MSLL_resmatrix["r"],
                 MSLL_resmatrix["p"]  ,
                 3 )

names(allparsode)=defparnames; #assign names to parameters

cat("best para ")
allparsode



# RUN ODE
#print(sprintf(' start run ode ' ))
INC <- c(  L0_10000-A0_10000, 0, A0_10000,  V_10000 );
names(INC)= namevar; #assign names to parameters
```

```
cat("best INC 10000")

INC



odestack=NULL

IVmlin = NULL

#  odestack=try(ode( func = mvequations,  y = INC, times = t_al, parms=allparsode, method ="daspk")); #runs the ODE equations


odestack=try(lsoda( INC, t_al,mvequations_di,parms=allparsode, atol=atolv,rtol=rtolv)); #runs the ODE equations

if (length(odestack)==1) {cat('!!unresolvable integrator error - triggering early return from optimizer!!'); return(eturmerror) } #catching errors that might happen during fitting

IVmlin =odestack[match( t_ivl_10 ,odestack[,1]),Vpos+1]; #extract values for virus load at time points corresponding to experimental measurements


cat("sim V 10000 ")

IVmlin


#plot( t_ivl_10,   m_ivl_10000)


if (sum(is.na(IVmlin))>0) {cat('!!ODE 10000fail VL NA return!!'); return(eturmerror) } #catching errors that might happen during fitting

if (sum( is.infinite(10^IVmlin))>0) {cat('!!ODE 10000fail VL INF correct!!');  return(eturmerror) } # IVmlin[ which( IVmlin>300  ) ] <-300 } #catching errors that might happen during fitting



Smlin=odestack[match(t_al,odestack[,1]),Spos+1]; #extract values for virus load at time points corresponding to experimental measurements

Imlin=odestack[match( t_al ,odestack[,1]),Ipos+1]; #extract values for virus load at time points corresponding to experimental measurements

Amlin_10000=odestack[match(t_al ,odestack[,1]),Apos+1]; #extract values for virus load at time points corresponding to experimental measurements

Lmscal_10000 = Smlin + Imlin + Amlin_10000
```

```
#if (Lmscal_10000[length(Lmscal_10000)]< 2000) {cat('!!ODE 10000faIL LOW TLYM');
return(eturmerror) }

IVmlong_10000 =odestack[match( t_al ,odestack[,1]),Vpos+1]; #extract values for virus load at time
points corresponding to experimental measurements

# correct lod
if (IVmlin[1] <= 0.3) {IVmlin[1]  <- 0.3 }
if (IVmlin[7] <= 0.3) {IVmlin[7]  <- 0.3 }
if (IVmlin[8] <= 0.3) {IVmlin[8]  <- 0.3 }

RSS_IV     <-    log( 10^IVmlin )  - log( 10^m_ivl_10000 )

loglik_IV_10000_bis  <-  -(n_days_ivl_10 /2)*  log(  (2*pi/n_days_ivl_10) * ( t( RSS_IV ) %*% RSS_IV )
) -(n_days_ivl_10 /2)

cat("loglik_IV_10000_bis ")
loglik_IV_10000_bis

IVmlin_10000  <- IVmlin

INC <- c(  L0_10000-A0_1000, 0, A0_1000,  V_1000 );
names(INC)= namevar; #assign names to parameters
odestack=NULL
IVmlin = NULL
```

```
#  odestack=try(ode( func = mvequations,  y = INC, times = t_al, parms=allparsode, method ="daspk")); #runs the ODE equations

odestack=try(lsoda( INC, t_al,mvequations_di,parms=allparsode, atol=atolv,rtol=rtolv)); #runs the ODE equations

if (length(odestack)==1) {cat('!!unresolvable integrator error - triggering early return from optimizer!!'); return(eturmerror) } #catching errors that might happen during fitting

lVmlin =odestack[match( t_ivl_10 ,odestack[,1]),Vpos+1]; #extract values for virus load at time points corresponding to experimental measurements

if (sum(is.na(lVmlin))>0) {cat('!!ODE 10000fail VL NA return!!'); return(eturmerror) } #catching errors that might happen during fitting

if (sum( is.infinite(10^lVmlin))>0) {cat('!!ODE 10000fail VL INF correct!!');  return(eturmerror) } # lVmlin[ which( lVmlin>300  ) ] <-300 } #catching errors that might happen during fitting


Smlin=odestack[match(t_al,odestack[,1]),Spos+1]; #extract values for virus load at time points corresponding to experimental measurements

Imlin=odestack[match( t_al ,odestack[,1]),Ipos+1]; #extract values for virus load at time points corresponding to experimental measurements

Amlin_1000 =odestack[match(t_al ,odestack[,1]),Apos+1]; #extract values for virus load at time points corresponding to experimental measurements

Lmscal_1000 = Smlin + Imlin + Amlin_1000

lVmlong_1000 =odestack[match( t_al ,odestack[,1]),Vpos+1]; #extract values for virus load at time points corresponding to experimental measurements


# correct lod

if (lVmlin[1] <= 0.3) {lVmlin[1]  <- 0.3 }

if (lVmlin[2] <= 0.3) {lVmlin[2]  <- 0.3 }

if (lVmlin[8] <= 0.3) {lVmlin[8]  <- 0.3 }


RSS_IV     <-    log(  10^lVmlin )  - log(  10^m_ivl_1000 )

loglik_IV_1000_bis  <-  -(n_days_ivl_10 /2)*  log(  (2*pi/n_days_ivl_10) * ( t( RSS_IV ) %*% RSS_IV )  ) -(n_days_ivl_10 /2)


cat("loglik_IV_1000_bis ")

loglik_IV_1000_bis
```

```
IVmlin_1000  <- IVmlin



INC <- c(  L0_10000-A0_100, 0, A0_100,  V_100 );

names(INC)= namevar; #assign names to parameters

odestack=NULL

IVmlin = NULL



#  odestack=try(ode( func = mvequations,  y = INC, times = t_al, parms=allparsode, method ="daspk")); #runs the ODE equations

odestack=try(lsoda( INC, t_al,mvequations_di,parms=allparsode, atol=atolv,rtol=rtolv)); #runs the ODE equations

if (length(odestack)==1) {cat('!!unresolvable integrator error - triggering early return from optimizer!!'); return(eturmerror) } #catching errors that might happen during fitting

IVmlin =odestack[match( t_ivl_10 ,odestack[,1]),Vpos+1]; #extract values for virus load at time points corresponding to experimental measurements

if (sum(is.na(IVmlin))>0) {cat('!!ODE 10000fail VL NA return!!'); return(eturmerror) } #catching errors that might happen during fitting

if (sum( is.infinite(10^IVmlin))>0) {cat('!!ODE 10000fail VL INF correct!!');  return(eturmerror) } # IVmlin[ which( IVmlin>300  ) ] <-300 } #catching errors that might happen during fitting


Smlin=odestack[match(t_al,odestack[,1]),Spos+1]; #extract values for virus load at time points corresponding to experimental measurements

Imlin=odestack[match( t_al ,odestack[,1]),Ipos+1]; #extract values for virus load at time points corresponding to experimental measurements

Amlin_100=odestack[match(t_al ,odestack[,1]),Apos+1]; #extract values for virus load at time points corresponding to experimental measurements

Lmscal_100 = Smlin + Imlin + Amlin_100


IVmlong_100 =odestack[match( t_al ,odestack[,1]),Vpos+1]; #extract values for virus load at time points corresponding to experimental measurements
```

```
# correct lod

if (IVmlin[1] <= 0.3) {IVmlin[1]  <- 0.3 }

if (IVmlin[2] <= 0.3) {IVmlin[2]  <- 0.3 }

if (IVmlin[8] <= 0.3) {IVmlin[8]  <- 0.3 }


RSS_IV     <-    log(  10^IVmlin )  - log(  10^m_ivl_100 )

loglik_IV_100_bis  <-  -(n_days_ivl_10 /2)*  log(  (2*pi/n_days_ivl_10) * ( t( RSS_IV ) %*% RSS_IV )  ) - (n_days_ivl_10 /2)


cat("loglik_IV_100_bis ")

loglik_IV_100_bis


IVmlin_100  <- IVmlin


INC <- c(  L0_10000-A0_10, 0, A0_10,  V_10 );


names(INC)= namevar; #assign names to parameters

odestack=NULL

IVmlin = NULL


#  odestack=try(ode( func = mvequations,  y = INC, times = t_al, parms=allparsode, method ="daspk")); #runs the ODE equations

odestack=try(lsoda( INC, t_al,mvequations_di,parms=allparsode, atol=atolv,rtol=rtolv)); #runs the ODE equations

if (length(odestack)==1) {cat('!!unresolvable integrator error - triggering early return from optimizer!!'); return(eturmerror) } #catching errors that might happen during fitting

IVmlin =odestack[match( t_ivl_10 ,odestack[,1]),Vpos+1]; #extract values for virus load at time points corresponding to experimental measurements

if (sum(is.na(IVmlin))>0) {cat('!!ODE 10000fail VL NA return!!'); return(eturmerror) } #catching errors that might happen during fitting
```

```
if (sum( is.infinite(10^IVmlin))>0) {cat('!!ODE 10000fail VL INF correct!!');  return(eturmerror) } # IVmlin[ which( IVmlin>300  ) ] <-300 } #catching errors that might happen during fitting


Smlin=odestack[match(t_al,odestack[,1]),Spos+1]; #extract values for virus load at time points corresponding to experimental measurements

Imlin=odestack[match( t_al ,odestack[,1]),Ipos+1]; #extract values for virus load at time points corresponding to experimental measurements

Amlin_10 =odestack[match(t_al ,odestack[,1]),Apos+1]; #extract values for virus load at time points corresponding to experimental measurements

Lmscal_10 = Smlin + Imlin + Amlin_10


IVmlong_10 =odestack[match( t_al ,odestack[,1]),Vpos+1]; #extract values for virus load at time points corresponding to experimental measurements


# correct lod

if (IVmlin[1] <= 0.3) {IVmlin[1]  <- 0.3 }

if (IVmlin[2] <= 0.3) {IVmlin[2]  <- 0.3 }

if (IVmlin[8] <= 0.3) {IVmlin[8]  <- 0.3 }


RSS_IV     <-    log(  10^IVmlin )  - log(  10^m_ivl_10 )

loglik_IV_10_bis  <-  -(n_days_ivl_10 /2)*  log(  (2*pi/n_days_ivl_10) * ( t( RSS_IV ) %*% RSS_IV )  ) - (n_days_ivl_10 /2)


cat("n_days_ivl_10 ")

n_days_ivl_10


cat("loglik_IV_10_bis ")

loglik_IV_10_bis


IVmlin_10  <- IVmlin
```

```
INC <- c(  L0_10000-A0_1, 0, A0_1,  V_1 );

names(INC)= namevar; #assign names to parameters

odestack=NULL

IVmlin = NULL

#  odestack=try(ode( func = mvequations,  y = INC, times = t_al, parms=allparsode, method ="daspk")); #runs the ODE equations

odestack=try(lsoda( INC, t_al,mvequations_di,parms=allparsode, atol=atolv,rtol=rtolv)); #runs the ODE equations

if (length(odestack)==1) {cat('!!unresolvable integrator error - triggering early return from optimizer!!'); return(eturmerror) } #catching errors that might happen during fitting

IVmlin =odestack[match( t_ivl_1 ,odestack[,1]),Vpos+1]; #extract values for virus load at time points corresponding to experimental measurements

cat("sim V1 ")

IVmlin

if (sum(is.na(IVmlin))>0) {cat('!!ODE 10000fail VL NA return!!'); return(eturmerror) } #catching errors that might happen during fitting

if (sum( is.infinite(10^IVmlin))>0) {cat('!!ODE 10000fail VL INF correct!!');   IVmlin[ which( IVmlin>300 ) ] <-300 } #catching errors that might happen during fitting

Smlin=odestack[match(t_al,odestack[,1]),Spos+1]; #extract values for virus load at time points corresponding to experimental measurements

Imlin=odestack[match( t_al ,odestack[,1]),Ipos+1]; #extract values for virus load at time points corresponding to experimental measurements

Amlin_1 =odestack[match(t_al ,odestack[,1]),Apos+1]; #extract values for virus load at time points corresponding to experimental measurements

Lmscal_1 = Smlin + Imlin + Amlin_1
```

```
IVmlong_1 =odestack[match( t_al ,odestack[,1]),Vpos+1]; #extract values for virus load at time points corresponding to experimental measurements


# correct lod

if (IVmlin[1] <= 0.3) {IVmlin[1]  <- 0.3 }

if (IVmlin[2] <= 0.3) {IVmlin[2]  <- 0.3 }

if (IVmlin[3] <= 0.3) {IVmlin[3]  <- 0.3 }


RSS_IV     <-    log(  10^IVmlin )  - log(  10^m_ivl_1 )

loglik_IV_1_bis  <-  -(n_days_ivl_1 /2)*  log(  (2*pi/n_days_ivl_1) * ( t( RSS_IV ) %*% RSS_IV )  ) - (n_days_ivl_1 /2)


cat("n_days_ivl_1 ")

n_days_ivl_1


cat("loglik_IV_1_bis ")

loglik_IV_1_bis


IVmlin_1  <- IVmlin


odestack=NULL

IVmlin = NULL


loglik_bis <-  loglik_IV_10000_bis   +  loglik_IV_1000_bis   +loglik_IV_100_bis   + loglik_IV_10_bis   + loglik_IV_1_bis


cat("loglik_bis  ")

loglik_bis


cat("loglik_ref  ")
```

```
MSLL_resmatrix["LOGLIK"]

#####################



####################################################





#MLE <- data.table(param = parnames,

#               estimates = MLE_par,

#              sd = MLE_SE,

#             aicc =  MLE_par*0+ datasim$AICC[1],

#            loglik = MLE_par*0-MLE_estimates$value )


#             TABMLE  <-  kable(MLE)


#            kable(data.table(MSLL_resmatrix))




datafit  <- data.frame(  TCID = c( t_al*0+10^4,   t_al*0+10^3,t_al*0+10^2, t_al*0+10^1, t_al*0+10^0)
,
                      X50. =  c( IVmlong_10000,IVmlong_1000, IVmlong_100, IVmlong_10, IVmlong_1  ) ,

                      #mes =  c( m_ivl_10000, m_ivl_1000, m_ivl_100, m_ivl_10, m_ivl_1  ) ,

                      tlym = c(Lmscal_10000, Lmscal_1000,Lmscal_100,Lmscal_10,Lmscal_1 ),

                      tcell = c(Amlin_10000, Amlin_1000, Amlin_100, Amlin_10, Amlin_1    ),

                      t =   c(  t_al, t_al, t_al, t_al,   t_al   ) )



#####################################################
```

```
fig_IV <-    ggplot() +
  #geom_ribbon(aes(ymin = X5., ymax = X95.), fill = "orange", alpha = 0.35) +
  geom_line(data = data94, aes(x = t, y = mes, color=as.factor(TCID) ),  size =0.25) +
  geom_line(data = datafit, aes(x = t, y = X50., color=as.factor(TCID)) , size =1.5, alpha=0.75) +
  geom_point(data = data94,  aes(x = t, y = mes, color=as.factor(TCID)),     size=1.5) +
  #geom_line(mapping = aes( y = si,m_ivl),linetype = "dashed",size=2, color = "blue") +
  labs(x = "Days post infection", y =bquote(  "Log" ~  TCID[50] ~  "/" ~  10^6 ~  "PBMC"))+
  ggtitle("Infectious virus ")  +
  #annotate(geom="text", x=25, y=4, label=mcqii, color="black")+
  labs(color = "Inoculum TCID=")+
  scale_fill_manual(values=cbbPalette)+
  scale_x_continuous(limits=c(0, 70), breaks=c(0,  3,  5,  7,  9, 11, 14, 18, 25, 70) )+
  annotate(geom="text", x=10, y=6,size=3,
           label= paste("Loglik= ",round(as.numeric(MSLL_resmatrix["LOGLIK"] ),2), sep=""),
           color="black")+
  annotate(geom="text", x=40, y=6,size=3,
           label= paste("AICC= ",round(as.numeric(MSLL_resmatrix["AICC"] ),2), sep=""),
           color="black")



fig_L <-   ggplot() +
  #geom_ribbon(aes(ymin = X5., ymax = X95.), fill = "orange", alpha = 0.35) +
  geom_point(data = mesacmv ,  aes(x = Day, y = Total.lymphocytes ),      size=1) +
  geom_line(data = mesacmv ,  aes(x = Day, y = Total.lymphocytes, group=as.factor(ID) ),       size=0.25)
+
  geom_line(data = datafit, aes(x = t, y = tlym, color=as.factor(TCID)) ,  size=1.5, aplha= 0.8) +
  #geom_line(mapping = aes(y = sim_tlym),linetype = "dashed", color = "blue",size=2) +
  labs(x = "Day", y = expression( "Cells/" ~ mu ~ "l") )  +
```

```
  ggtitle(" Total lymphocytes ") +
  scale_fill_manual(values=cbbPalette)+
  theme(legend.position = "none") +
  scale_x_continuous(breaks=c(0,  3,  5,  7,  9, 11, 14, 18, 25,35,50,70) )


"Generatefigure A"


fig_A <-     ggplot() +
  #geom_ribbon(aes(ymin = X5., ymax = X95.), fill = "orange", alpha = 0.35) +
  #geom_point(data = mesacmv ,  aes(x = Day , y = MV.specific.T.cells ),      size=1) +
  #geom_line(data = mesacmv ,  aes(x = Day, y = MV.specific.T.cells, group=as.factor(ID) ),
size=0.25) +
  geom_line(data = datafit, aes(x = t, y =tcell, color=as.factor(TCID)) ,  size=1.5, alpha=0.7) +
  # geom_line(mapping = aes(y = sim_tcell),linetype = "dashed", color = "blue",size=2) +
  labs(x = "Day", y = " IFN-?? spot-forming cells per microliter of blood") +
  ggtitle("MV-specific T cell response") +
  scale_fill_manual(values=cbbPalette)+
  theme(legend.position = "none")  +
  scale_x_continuous(breaks=c(0,  3,  5,  7,  9, 11, 14, 18, 25,35,50,70) )


################################################################


"combinefigdynamics"
mdlfrna <- plot_grid(  fig_IV, fig_L,  fig_A ,
                    labels = c("A", "B", "c"),
                    align="h", ncol=1,
                    label_size = 10)
```

```
mdlfrna

cat("loglik_bis  ")
loglik_bis

"savecombinefigdynamics"

save_plot(paste("f-mv-model_1994_hpcMS_ICDIFXpara_46u_050_eflo08XXLL.pdf", sep=""), mdlfrna,
ncol = 1, nrow = 3 )
```
