# Supplementary material for: Measles Infection Dose Responses: Insights from Mathematical Modeling
Source: Bull Math Biol. 2024 Jun 9;86(7):85. doi: 10.1007/s11538-024-01305-0 (PMC11162976; doi:10.1007/s11538-024-01305-0)
Supplement: Supplementary file 3 — (pdf 512 KB) [file 11538_2024_1305_MOESM3_ESM.pdf]

```
##################################################################################
#fitting data from IAV infections at different inoculum doses
#written by Andreas Handel (ahandel@uga.edu)
#last changed on 10/28/2017
#####################


cat(  "set workingdirectory HPC")


setwd("~/Anet_MEV_NUS/mv_rcode")


#############################################################
rm(list=ls()) #this clears the workspace to make sure no leftover variables are floating around. Not strictly needed
graphics.off(); #close all graphics windows
library(deSolve)  #loads ODE solver package
library(nloptr) #for fitting
 #for data prep
library(snow) #for parallel computing
library(matrixcalc)
library(rlang)
library(ggplot2)
library(gridExtra)
library(dplyr)


library(forcats)


library(cowplot)
#https://mran.microsoft.com/snapshot/2017-03-04/web/packages/EGRET/vignettes/rResid.html
library(tgp)


#setwd("~/Anet_MEV_NUS/mv_rcode")
```

```
#  source('iavequations.R') #load function that contains ODE model


source('mvequations.R') #load mv model


source('mvequations_di.R') # model proliferation T cell

source('mvequations_qs.R') # model proliferation T cell

####################################
# multistart
#number of multisart
set.seed(1)

NMS <- 1

NMS_seq  <-   seq(1, NMS )
NMS_seq
############################################
#function that fits the ODE model to data
#########################

#########################


# Step 2: Derive the likelihood function
#cbbPalette <- c( "#E69F00", "#009E73",  "#0072B2", "#D55E00", "#CC79A7")
```

```
cbbPalette <- c( "red",   "blue", "orange","magenta"  , "green")

# To use for fills, add

# Likelihood function

getlog_like <- function(allestimates, pos, parafocus){
  #one monkey at a time

  # print(sprintf('Running LL Monkey ID = %f _ %s ',ii, mcqii ))
  # times
  # X <- as.matrix(X  );
  loglik = NULL;
  loglik_IV_10000  = NULL;
  loglik_IV_1000  = NULL;
  loglik_IV_100  = NULL;
  loglik_IV_10  = NULL;
  loglik_IV_1 = NULL;

  #data
  #   Y <- as.matrix( Y);

  # times
  #vector for integration times
  t_tlym<- as.matrix( day_tlym );
  t_ivl  <- as.matrix( day_ivl );
  # counts of data
  #  N      <- nrow(X)
```

```
n_times  <- length(times);
n_days_tlym  <- length( day_tlym );
n_days_ivl  <- length( day_ivl );
#1994

# Data
#data accummulation code
#  allvirus_data = c(allvirus_data, virusdata)

m_tlym  <- as.matrix(  mes_tlym  );
m_tcell  <- as.matrix(  mes_tcell );
m_ivl  <- as.matrix(  mes_ivl  );

#print(sprintf(' prep ode ' ))
# times
###prep  ode
#parameters

parsode = allestimates; #transform parameters back to exponential since we fit in log space

if ( pos > 0) { parsode[pos]  <- parafocus }

#  model Initial conditions
# efi <- tail( parsode,1)
```

```
A0_10000 <-   parsode[1];
A0_1000 <-  parsode[2];
A0_100 <-  parsode[3];
A0_10 <-  parsode[4];
A0_1 <-  parsode[5];



V_10000  <-  parsode[6];
V_1000  <-  parsode[7];
V_100  <-  parsode[8];
V_10  <-  parsode[9];
V_1  <-  parsode[10];

L0_10000 <-  3906 #parsode[11];



#names(theta)=parnames; #assign names to parameters
#print(sprintf(' prep ode para assign ' ))
#print(sprintf('A0 LL = %f  ',A0_10000 ))

#Define parameter value

allparsode <-   c( 0.028, #  parsode[ 12] ,
                2.6 , #  parsode[13] ,
                parsode[11] ,
                0.5 ,
                parsode[12] ,
                1.11, #  parsode[12] ,
                parsode[13] ,
```

```
                0.025,
                0.016, #  parsode[18] ,
                parsode[14] ,
                3 )

  names(allparsode)=defparnames; #assign names to parameters

  #  print(sprintf('td LL  = %f  ',allparsode[2] ))
  # times
  # RUN ODE
  #print(sprintf(' start run ode ' ))

  INC <- c(  L0_10000-A0_10000, 0, A0_10000,  V_10000 );

  names(INC)= namevar; #assign names to parameters

  odestack=NULL
  lVmlin = NULL
  #  odestack=try(ode( func = mvequations,  y = INC, times = t_al, parms=allparsode, method
="daspk")); #runs the ODE equations

  odestack=try(lsoda( INC, t_al,mvequations_di,parms=allparsode, atol=atolv,rtol=rtolv)); #runs the
ODE equations
  if (length(odestack)==1) {cat('!!unresolvable integrator error - triggering early return from
optimizer!!'); return(eturmerror) } #catching errors that might happen during fitting
  lVmlin =odestack[match( t_ivl_10 ,odestack[,1]),Vpos+1]; #extract values for virus load at time
points corresponding to experimental measurements
  #plot( t_ivl_10,   m_ivl_10000)
  if (sum(is.na(lVmlin))>0) {cat('!!ODE 10000fail VL NA return!!'); return(eturmerror) } #catching
errors that might happen during fitting
  if (sum( is.infinite(10^lVmlin))>0) {cat('!!ODE 10000fail VL INF correct!!');   lVmlin[ which(
lVmlin>300  ) ] <-300 } #catching errors that might happen during fitting
```

```
  Smlin=odestack[match(t_al,odestack[,1]),Spos+1]; #extract values for virus load at time points corresponding to experimental measurements

  Imlin=odestack[match( t_al ,odestack[,1]),Ipos+1]; #extract values for virus load at time points corresponding to experimental measurements

  Amlin_10000=odestack[match(t_al ,odestack[,1]),Apos+1]; #extract values for virus load at time points corresponding to experimental measurements

  Lmscal_10000 = Smlin + Imlin + Amlin_10000

  #if (Lmscal_10000[length(Lmscal_10000)]< 2000) {cat('!!ODE 10000faIL LOW TLYM'); return(eturmerror) }


  IVmlong_10000 =odestack[match( t_al ,odestack[,1]),Vpos+1]; #extract values for virus load at time points corresponding to experimental measurements


  # correct lod

  if (IVmlin[1] <= 0.3) {IVmlin[1]  <- 0.3 }

  if (IVmlin[7] <= 0.3) {IVmlin[7]  <- 0.3 }

  if (IVmlin[8] <= 0.3) {IVmlin[8]  <- 0.3 }


  RSS_IV     <-    log( 10^IVmlin )  - log( 10^m_ivl_10000 )


  loglik_IV_10000 <-  -(n_days_ivl_10 /2)*  log(  (2*pi/n_days_ivl_10) * ( t( RSS_IV ) %*% RSS_IV )  ) - (n_days_ivl_10 /2)


  IVmlin_10000  <- IVmlin




  INC <- c(  L0_10000-A0_1000, 0, A0_1000,  V_1000 );

  names(INC)= namevar; #assign names to parameters

  odestack=NULL

  IVmlin = NULL
```

```
  #  odestack=try(ode( func = mvequations,  y = INC, times = t_al, parms=allparsode, method ="daspk")); #runs the ODE equations

  odestack=try(lsoda( INC, t_al,mvequations_di,parms=allparsode, atol=atolv,rtol=rtolv)); #runs the ODE equations

  if (length(odestack)==1) {cat('!!unresolvable integrator error - triggering early return from optimizer!!'); return(eturmerror) } #catching errors that might happen during fitting

  lVmlin =odestack[match( t_ivl_10 ,odestack[,1]),Vpos+1]; #extract values for virus load at time points corresponding to experimental measurements

  if (sum(is.na(lVmlin))>0) {cat('!!ODE 10000fail VL NA return!!'); return(eturmerror) } #catching errors that might happen during fitting

  if (sum( is.infinite(10^lVmlin))>0) {cat('!!ODE 10000fail VL INF correct!!');   lVmlin[ which( lVmlin>300  ) ] <-300 } #catching errors that might happen during fitting


  Smlin=odestack[match(t_al,odestack[,1]),Spos+1]; #extract values for virus load at time points corresponding to experimental measurements

  Imlin=odestack[match( t_al ,odestack[,1]),Ipos+1]; #extract values for virus load at time points corresponding to experimental measurements

  Amlin_1000 =odestack[match(t_al ,odestack[,1]),Apos+1]; #extract values for virus load at time points corresponding to experimental measurements

  Lmscal_1000 = Smlin + Imlin + Amlin_1000

  lVmlong_1000 =odestack[match( t_al ,odestack[,1]),Vpos+1]; #extract values for virus load at time points corresponding to experimental measurements


  # correct lod

  if (lVmlin[1] <= 0.3) {lVmlin[1]  <- 0.3 }

  if (lVmlin[2] <= 0.3) {lVmlin[2]  <- 0.3 }

  if (lVmlin[8] <= 0.3) {lVmlin[8]  <- 0.3 }


  RSS_lV     <-    log(  10^lVmlin )  - log(  10^m_ivl_1000 )

  loglik_lV_1000 <-  -(n_days_ivl_10 /2)*  log(  (2*pi/n_days_ivl_10) * ( t( RSS_lV ) %*% RSS_lV )  ) - (n_days_ivl_10 /2)


  lVmlin_1000  <- lVmlin
```

```
INC <- c(  L0_10000-A0_100, 0, A0_100,  V_100 );

names(INC)= namevar; #assign names to parameters

odestack=NULL

IVmlin = NULL

#  odestack=try(ode( func = mvequations,  y = INC, times = t_al, parms=allparsode, method ="daspk")); #runs the ODE equations

odestack=try(lsoda( INC, t_al,mvequations_di,parms=allparsode, atol=atolv,rtol=rtolv)); #runs the ODE equations

if (length(odestack)==1) {cat('!!unresolvable integrator error - triggering early return from optimizer!!'); return(eturmerror) } #catching errors that might happen during fitting

IVmlin =odestack[match( t_ivl_10 ,odestack[,1]),Vpos+1]; #extract values for virus load at time points corresponding to experimental measurements

if (sum(is.na(IVmlin))>0) {cat('!!ODE 10000fail VL NA return!!'); return(eturmerror) } #catching errors that might happen during fitting

if (sum( is.infinite(10^IVmlin))>0) {cat('!!ODE 10000fail VL INF correct!!');   IVmlin[ which( IVmlin>300  ) ] <-300 } #catching errors that might happen during fitting

Smlin=odestack[match(t_al,odestack[,1]),Spos+1]; #extract values for virus load at time points corresponding to experimental measurements

Imlin=odestack[match( t_al ,odestack[,1]),Ipos+1]; #extract values for virus load at time points corresponding to experimental measurements

Amlin_100=odestack[match(t_al ,odestack[,1]),Apos+1]; #extract values for virus load at time points corresponding to experimental measurements

Lmscal_100 = Smlin + Imlin + Amlin_100

IVmlong_100 =odestack[match( t_al ,odestack[,1]),Vpos+1]; #extract values for virus load at time points corresponding to experimental measurements

# correct lod
```

```
if (IVmlin[1] <= 0.3) {IVmlin[1]  <- 0.3 }

if (IVmlin[2] <= 0.3) {IVmlin[2]  <- 0.3 }

if (IVmlin[8] <= 0.3) {IVmlin[8]  <- 0.3 }


RSS_IV    <-   log(  10^IVmlin )  - log(  10^m_ivl_100 )

loglik_IV_100 <-  -(n_days_ivl_10 /2)*  log(  (2*pi/n_days_ivl_10) * ( t( RSS_IV ) %*% RSS_IV )  ) - (n_days_ivl_10 /2)


IVmlin_100  <- IVmlin


INC <- c(  L0_10000-A0_10, 0, A0_10,  V_10 );


names(INC)= namevar; #assign names to parameters

odestack=NULL

IVmlin = NULL


#  odestack=try(ode( func = mvequations,  y = INC, times = t_al, parms=allparsode, method ="daspk")); #runs the ODE equations

odestack=try(lsoda( INC, t_al,mvequations_di,parms=allparsode, atol=atolv,rtol=rtolv)); #runs the ODE equations

if (length(odestack)==1) {cat('!!unresolvable integrator error - triggering early return from optimizer!!'); return(eturmerror) } #catching errors that might happen during fitting

IVmlin =odestack[match( t_ivl_10 ,odestack[,1]),Vpos+1]; #extract values for virus load at time points corresponding to experimental measurements

if (sum(is.na(IVmlin))>0) {cat('!!ODE 10000fail VL NA return!!'); return(eturmerror) } #catching errors that might happen during fitting

if (sum( is.infinite(10^IVmlin))>0) {cat('!!ODE 10000fail VL INF correct!!');   IVmlin[ which( IVmlin>300  ) ] <-300 } #catching errors that might happen during fitting


Smlin=odestack[match(t_al,odestack[,1]),Spos+1]; #extract values for virus load at time points corresponding to experimental measurements
```

```
  Imlin=odestack[match( t_al ,odestack[,1]),Ipos+1]; #extract values for virus load at time points corresponding to experimental measurements

  Amlin_10 =odestack[match(t_al ,odestack[,1]),Apos+1]; #extract values for virus load at time points corresponding to experimental measurements

  Lmscal_10 = Smlin + Imlin + Amlin_10


  IVmlong_10 =odestack[match( t_al ,odestack[,1]),Vpos+1]; #extract values for virus load at time points corresponding to experimental measurements


  # correct lod

  if (IVmlin[1] <= 0.3) {IVmlin[1]  <- 0.3 }

  if (IVmlin[2] <= 0.3) {IVmlin[2]  <- 0.3 }

  if (IVmlin[8] <= 0.3) {IVmlin[8]  <- 0.3 }


  RSS_IV     <-    log(  10^IVmlin )  - log(  10^m_ivl_10 )

  loglik_IV_10 <-  -(n_days_ivl_10 /2)*  log(  (2*pi/n_days_ivl_10) * ( t( RSS_IV ) %*% RSS_IV )  ) - (n_days_ivl_10 /2)


  IVmlin_10  <- IVmlin


  INC <- c(  L0_10000-A0_1, 0, A0_1,  V_1 );


  names(INC)= namevar; #assign names to parameters

  odestack=NULL

  IVmlin = NULL


  #  odestack=try(ode( func = mvequations,  y = INC, times = t_al, parms=allparsode, method ="daspk")); #runs the ODE equations

  odestack=try(lsoda( INC, t_al,mvequations_di,parms=allparsode, atol=atolv,rtol=rtolv)); #runs the ODE equations
```

```
  if (length(odestack)==1) {cat('!!unresolvable integrator error - triggering early return from optimizer!!'); return(eturmerror) } #catching errors that might happen during fitting

  IVmlin =odestack[match( t_ivl_1 ,odestack[,1]),Vpos+1]; #extract values for virus load at time points corresponding to experimental measurements

  if (sum(is.na(IVmlin))>0) {cat('!!ODE 10000fail VL NA return!!'); return(eturmerror) } #catching errors that might happen during fitting

  if (sum( is.infinite(10^IVmlin))>0) {cat('!!ODE 10000fail VL INF correct!!');   IVmlin[ which( IVmlin>300  ) ] <-300 } #catching errors that might happen during fitting


  Smlin=odestack[match(t_al,odestack[,1]),Spos+1]; #extract values for virus load at time points corresponding to experimental measurements

  Imlin=odestack[match( t_al ,odestack[,1]),Ipos+1]; #extract values for virus load at time points corresponding to experimental measurements

  Amlin_1 =odestack[match(t_al ,odestack[,1]),Apos+1]; #extract values for virus load at time points corresponding to experimental measurements

  Lmscal_1 = Smlin + Imlin + Amlin_1


  IVmlong_1 =odestack[match( t_al ,odestack[,1]),Vpos+1]; #extract values for virus load at time points corresponding to experimental measurements


  # correct lod

  if (IVmlin[1] <= 0.3) {IVmlin[1]  <- 0.3 }

  if (IVmlin[2] <= 0.3) {IVmlin[2]  <- 0.3 }

  if (IVmlin[3] <= 0.3) {IVmlin[3]  <- 0.3 }


  RSS_IV     <-    log(  10^IVmlin )  - log(  10^m_ivl_1 )

  loglik_IV_1 <-  -(n_days_ivl_1 /2)*  log(  (2*pi/n_days_ivl_1) * ( t( RSS_IV ) %*% RSS_IV )  ) - (n_days_ivl_1 /2)


  IVmlin_1  <- IVmlin


  odestack=NULL

  IVmlin = NULL
```

```
  loglik <-  loglik_IV_10000  +  loglik_IV_1000  +loglik_IV_100  + loglik_IV_10  +  loglik_IV_1
  #print(sprintf(' end -loglik ' ))
  #print(sprintf(' end -loglik ' ))

  if (is.na(loglik))  {cat('!!na loglik!!'); return(eturmerror) }

  return(loglik)
}
##############################################

########################################################

# Likelihood function
log_like <- function(theta ){
  #one monkey at a time

  # print(sprintf('Running LL Monkey ID = %f _ %s ',ii, mcqii ))
  # times
  # X <- as.matrix(X  );
  loglik = NULL;
  loglik_IV_10000  = NULL;
  loglik_IV_1000  = NULL;
  loglik_IV_100  = NULL;
  loglik_IV_10  = NULL;
  loglik_IV_1 = NULL;



  #data
  #   Y <- as.matrix( Y);

  # times
```

```
#vector for integration times
t_tlym<- as.matrix( day_tlym );
t_ivl  <- as.matrix( day_ivl );
# counts of data
#  N       <- nrow(X)
n_times  <- length(times);
n_days_tlym  <- length( day_tlym );
n_days_ivl  <- length( day_ivl );
#1994




# Data
#data accummulation code
#  allvirus_data = c(allvirus_data, virusdata)

m_tlym  <- as.matrix(  mes_tlym  );
m_tcell  <- as.matrix(  mes_tcell );
m_ivl  <- as.matrix(  mes_ivl  );

#print(sprintf(' prep ode ' ))
# times
###prep  ode
#parameters
parsode = 10^(theta); #transform parameters back to exponential since we fit in log space

#  model Initial conditions
# efi <- tail( parsode,1)
```

```
A0_10000 <-    parsode[1];
A0_1000 <-  parsode[2];
A0_100 <-  parsode[3];
A0_10 <-  parsode[4];
A0_1 <-  parsode[5];




V_10000  <-  parsode[6];
V_1000  <-  parsode[7];
V_100  <-  parsode[8];
V_10  <-  parsode[9];
V_1  <-  parsode[10];

L0_10000 <-  3906 #parsode[11];




#names(theta)=parnames; #assign names to parameters
#print(sprintf(' prep ode para assign ' ))
#print(sprintf('A0 LL = %f  ',A0_10000 ))

#Define parameter value

allparsode <-   c( 0.028, #  parsode[ 12] ,
                 2.6 , #  parsode[13] ,
                  parsode[11] ,
                  0.5 ,
                  parsode[12] ,
                 1.11, #  parsode[12] ,
                  parsode[13] ,
```

```
                0.025,
               0.016, #  parsode[18] ,
                parsode[14] ,
                3 )

  names(allparsode)=defparnames; #assign names to parameters

  #  print(sprintf('td LL  = %f  ',allparsode[2] ))
  # times
  # RUN ODE
  #print(sprintf(' start run ode ' ))

  INC <- c(  L0_10000-A0_10000, 0, A0_10000,  V_10000 );

  names(INC)= namevar; #assign names to parameters

  odestack=NULL
  lVmlin = NULL
  #  odestack=try(ode( func = mvequations,  y = INC, times = t_al, parms=allparsode, method
="daspk")); #runs the ODE equations

  odestack=try(lsoda( INC, t_al,mvequations_di,parms=allparsode, atol=atolv,rtol=rtolv)); #runs the
ODE equations
    if (length(odestack)==1) {cat('!!unresolvable integrator error - triggering early return from
optimizer!!'); return(eturmerror) } #catching errors that might happen during fitting
    lVmlin =odestack[match( t_ivl_10 ,odestack[,1]),Vpos+1]; #extract values for virus load at time
points corresponding to experimental measurements
  #plot( t_ivl_10,   m_ivl_10000)
    if (sum(is.na(lVmlin))>0) {cat('!!ODE 10000fail VL NA return!!'); return(eturmerror) } #catching
errors that might happen during fitting
  if (sum( is.infinite(10^lVmlin))>0) {cat('!!ODE 10000fail VL INF correct!!');   lVmlin[ which(
lVmlin>300  ) ] <-300 } #catching errors that might happen during fitting
```

```
  Smlin=odestack[match(t_al,odestack[,1]),Spos+1]; #extract values for virus load at time points corresponding to experimental measurements

  Imlin=odestack[match( t_al ,odestack[,1]),Ipos+1]; #extract values for virus load at time points corresponding to experimental measurements

  Amlin_10000=odestack[match(t_al ,odestack[,1]),Apos+1]; #extract values for virus load at time points corresponding to experimental measurements

  Lmscal_10000 = Smlin + Imlin + Amlin_10000

  #if (Lmscal_10000[length(Lmscal_10000)]< 2000) {cat('!!ODE 10000faIL LOW TLYM'); return(eturmerror) }


  IVmlong_10000 =odestack[match( t_al ,odestack[,1]),Vpos+1]; #extract values for virus load at time points corresponding to experimental measurements


  # correct lod

  if (IVmlin[1] <= 0.3) {IVmlin[1]  <- 0.3 }

  if (IVmlin[7] <= 0.3) {IVmlin[7]  <- 0.3 }

  if (IVmlin[8] <= 0.3) {IVmlin[8]  <- 0.3 }


  RSS_IV     <-    log( 10^IVmlin )  - log( 10^m_ivl_10000 )


  loglik_IV_10000 <-  -(n_days_ivl_10 /2)*  log(  (2*pi/n_days_ivl_10) * ( t( RSS_IV ) %*% RSS_IV )  ) - (n_days_ivl_10 /2)


  IVmlin_10000  <- IVmlin




  INC <- c(  L0_10000-A0_1000, 0, A0_1000,  V_1000 );

  names(INC)= namevar; #assign names to parameters

  odestack=NULL

  IVmlin = NULL
```

```
  #  odestack=try(ode( func = mvequations,  y = INC, times = t_al, parms=allparsode, method ="daspk")); #runs the ODE equations

  odestack=try(lsoda( INC, t_al,mvequations_di,parms=allparsode, atol=atolv,rtol=rtolv)); #runs the ODE equations

  if (length(odestack)==1) {cat('!!unresolvable integrator error - triggering early return from optimizer!!'); return(eturmerror) } #catching errors that might happen during fitting

  IVmlin =odestack[match( t_ivl_10 ,odestack[,1]),Vpos+1]; #extract values for virus load at time points corresponding to experimental measurements

  if (sum(is.na(IVmlin))>0) {cat('!!ODE 10000fail VL NA return!!'); return(eturmerror) } #catching errors that might happen during fitting

  if (sum( is.infinite(10^IVmlin))>0) {cat('!!ODE 10000fail VL INF correct!!');   IVmlin[ which( IVmlin>300  ) ] <-300 } #catching errors that might happen during fitting


  Smlin=odestack[match(t_al,odestack[,1]),Spos+1]; #extract values for virus load at time points corresponding to experimental measurements

  Imlin=odestack[match( t_al ,odestack[,1]),Ipos+1]; #extract values for virus load at time points corresponding to experimental measurements

  Amlin_1000 =odestack[match(t_al ,odestack[,1]),Apos+1]; #extract values for virus load at time points corresponding to experimental measurements

  Lmscal_1000 = Smlin + Imlin + Amlin_1000

  IVmlong_1000 =odestack[match( t_al ,odestack[,1]),Vpos+1]; #extract values for virus load at time points corresponding to experimental measurements


  # correct lod

  if (IVmlin[1] <= 0.3) {IVmlin[1]  <- 0.3 }

  if (IVmlin[2] <= 0.3) {IVmlin[2]  <- 0.3 }

  if (IVmlin[8] <= 0.3) {IVmlin[8]  <- 0.3 }


  RSS_IV     <-    log(  10^IVmlin )  - log(  10^m_ivl_1000 )

  loglik_IV_1000 <-  -(n_days_ivl_10 /2)*  log(  (2*pi/n_days_ivl_10) * ( t( RSS_IV ) %*% RSS_IV )  ) - (n_days_ivl_10 /2)


  IVmlin_1000  <- IVmlin
```

```
  INC <- c(  L0_10000-A0_100, 0, A0_100,  V_100 );


  names(INC)= namevar; #assign names to parameters

  odestack=NULL

  IVmlin = NULL



  #  odestack=try(ode( func = mvequations,  y = INC, times = t_al, parms=allparsode, method ="daspk")); #runs the ODE equations

  odestack=try(lsoda( INC, t_al,mvequations_di,parms=allparsode, atol=atolv,rtol=rtolv)); #runs the ODE equations

  if (length(odestack)==1) {cat('!!unresolvable integrator error - triggering early return from optimizer!!'); return(eturmerror) } #catching errors that might happen during fitting

  IVmlin =odestack[match( t_ivl_10 ,odestack[,1]),Vpos+1]; #extract values for virus load at time points corresponding to experimental measurements

  if (sum(is.na(IVmlin))>0) {cat('!!ODE 10000fail VL NA return!!'); return(eturmerror) } #catching errors that might happen during fitting

  if (sum( is.infinite(10^IVmlin))>0) {cat('!!ODE 10000fail VL INF correct!!');   IVmlin[ which( IVmlin>300  ) ] <-300 } #catching errors that might happen during fitting


  Smlin=odestack[match(t_al,odestack[,1]),Spos+1]; #extract values for virus load at time points corresponding to experimental measurements

  Imlin=odestack[match( t_al ,odestack[,1]),Ipos+1]; #extract values for virus load at time points corresponding to experimental measurements

  Amlin_100=odestack[match(t_al ,odestack[,1]),Apos+1]; #extract values for virus load at time points corresponding to experimental measurements

  Lmscal_100 = Smlin + Imlin + Amlin_100


  IVmlong_100 =odestack[match( t_al ,odestack[,1]),Vpos+1]; #extract values for virus load at time points corresponding to experimental measurements


  # correct lod
```

```
  if (IVmlin[1] <= 0.3) {IVmlin[1]  <- 0.3 }

  if (IVmlin[2] <= 0.3) {IVmlin[2]  <- 0.3 }

  if (IVmlin[8] <= 0.3) {IVmlin[8]  <- 0.3 }


  RSS_IV     <-    log(  10^IVmlin )  - log(  10^m_ivl_100 )

  loglik_IV_100 <-  -(n_days_ivl_10 /2)*  log(  (2*pi/n_days_ivl_10) * ( t( RSS_IV ) %*% RSS_IV )  ) - (n_days_ivl_10 /2)


  IVmlin_100  <- IVmlin



  INC <- c(  L0_10000-A0_10, 0, A0_10,  V_10 );


  names(INC)= namevar; #assign names to parameters

  odestack=NULL

  IVmlin = NULL



  #  odestack=try(ode( func = mvequations,  y = INC, times = t_al, parms=allparsode, method ="daspk")); #runs the ODE equations

  odestack=try(lsoda( INC, t_al,mvequations_di,parms=allparsode, atol=atolv,rtol=rtolv)); #runs the ODE equations

  if (length(odestack)==1) {cat('!!unresolvable integrator error - triggering early return from optimizer!!'); return(eturmerror) } #catching errors that might happen during fitting

  IVmlin =odestack[match( t_ivl_10 ,odestack[,1]),Vpos+1]; #extract values for virus load at time points corresponding to experimental measurements

  if (sum(is.na(IVmlin))>0) {cat('!!ODE 10000fail VL NA return!!'); return(eturmerror) } #catching errors that might happen during fitting

  if (sum( is.infinite(10^IVmlin))>0) {cat('!!ODE 10000fail VL INF correct!!');   IVmlin[ which( IVmlin>300  ) ] <-300 } #catching errors that might happen during fitting


  Smlin=odestack[match(t_al,odestack[,1]),Spos+1]; #extract values for virus load at time points corresponding to experimental measurements
```

Imlin=odestack[match(t_al ,odestack[,1]),Ipos+1]; #extract values for virus load at time points corresponding to experimental measurements

Amlin_10 =odestack[match(t_al ,odestack[,1]),Apos+1]; #extract values for virus load at time points corresponding to experimental measurements

Lmscal_10 = Smlin + Imlin + Amlin_10

IVmlong_10 =odestack[match(t_al ,odestack[,1]),Vpos+1]; #extract values for virus load at time points corresponding to experimental measurements

correct lod

if (IVmlin[1] <= 0.3) {IVmlin[1] <- 0.3 }

if (IVmlin[2] <= 0.3) {IVmlin[2] <- 0.3 }

if (IVmlin[8] <= 0.3) {IVmlin[8] <- 0.3 }

RSS_IV <- log(10^IVmlin) - log(10^m_ivl_10)

loglik_IV_10 <- -(n_days_ivl_10 /2)* log((2*pi/n_days_ivl_10) * (t(RSS_IV) %*% RSS_IV)) - (n_days_ivl_10 /2)

IVmlin_10 <- IVmlin

INC <- c(L0_10000-A0_1, 0, A0_1, V_1);

names(INC)= namevar; #assign names to parameters

odestack=NULL

IVmlin = NULL

odestack=try(ode(func = mvequations, y = INC, times = t_al, parms=allparsode, method ="daspk")); #runs the ODE equations

odestack=try(lsoda(INC, t_al,mvequations_di,parms=allparsode, atol=atolv,rtol=rtolv)); #runs the ODE equations

```
  if (length(odestack)==1) {cat('!!unresolvable integrator error - triggering early return from optimizer!!'); return(eturmerror) } #catching errors that might happen during fitting

  IVmlin =odestack[match( t_ivl_1 ,odestack[,1]),Vpos+1]; #extract values for virus load at time points corresponding to experimental measurements

  if (sum(is.na(IVmlin))>0) {cat('!!ODE 10000fail VL NA return!!'); return(eturmerror) } #catching errors that might happen during fitting

  if (sum( is.infinite(10^IVmlin))>0) {cat('!!ODE 10000fail VL INF correct!!');   IVmlin[ which( IVmlin>300  ) ] <-300 } #catching errors that might happen during fitting


  Smlin=odestack[match(t_al,odestack[,1]),Spos+1]; #extract values for virus load at time points corresponding to experimental measurements

  Imlin=odestack[match( t_al ,odestack[,1]),Ipos+1]; #extract values for virus load at time points corresponding to experimental measurements

  Amlin_1 =odestack[match(t_al ,odestack[,1]),Apos+1]; #extract values for virus load at time points corresponding to experimental measurements

  Lmscal_1 = Smlin + Imlin + Amlin_1


  IVmlong_1 =odestack[match( t_al ,odestack[,1]),Vpos+1]; #extract values for virus load at time points corresponding to experimental measurements


  # correct lod

  if (IVmlin[1] <= 0.3) {IVmlin[1]  <- 0.3 }

  if (IVmlin[2] <= 0.3) {IVmlin[2]  <- 0.3 }

  if (IVmlin[3] <= 0.3) {IVmlin[3]  <- 0.3 }


  RSS_IV     <-    log(  10^IVmlin )  - log(  10^m_ivl_1 )

  loglik_IV_1 <-  -(n_days_ivl_1 /2)*  log(  (2*pi/n_days_ivl_1) * ( t( RSS_IV ) %*% RSS_IV )  ) - (n_days_ivl_1 /2)


  IVmlin_1  <- IVmlin


  odestack=NULL

  IVmlin = NULL
```

```
  loglik <-  loglik_IV_10000  +  loglik_IV_1000  +loglik_IV_100  + loglik_IV_10  +  loglik_IV_1
  #print(sprintf(' end -loglik ' ))
  #print(sprintf(' end -loglik ' ))

  if (is.na(loglik))  {cat('!!na loglik!!'); return(eturmerror) }

  return(-loglik)
}
########################################
##############################################
##############################################
########################################################
#wrapper function that runs parallel over different methods
#############################################################
outerfitfc <- function(NMS_seq  )
{

  fitAICC =NULL;
  MLE_estimates =NULL;
  para_guess =NULL;



  #selection of one start


  para_guess = log10(   (para_guess_MS[,NMS_seq ]))



  #error check
  #if (min(logub-para_guess)<0 | min(para_guess-loglb)<0)
  #{ print(sprintf('initial condition out of bound')); para_guess=pmax(pmin(logub,para_guess),loglb);
}
```

```
names(para_guess)=parnames; #assign names to parameters
#para_guess


MLE_estimates <- optim(fn=log_like,                  # Likelihood function
                    #par= log10(MLE_estimates$par),                  # Initial guess
                    par= para_guess,                  # Initial guess
                    lower = loglb,        # Lower bound on parameters
                    upper = logub,          # Upper bound on parameters
                    hessian=TRUE,
                    #method = "BFGS",
                    method = "L-BFGS-B",
                    control = list(maxit=10^9,   reltol= 1e-15,  factr = 1e-15))


MLE_par <-  10^(MLE_estimates$par)

if (as.numeric(MLE_estimates$value) == eturmerror) {
  cat('!!FULL EARLY RETURN OPTIM!');
  MLE_estimates$value = - MLE_estimates$value;
  MLE_SE <-  MLE_par *0
   #return(resvector)
} else {
  if ( is.singular.matrix(as.matrix(MLE_estimates$hessian), tol = 1e-08) )
  {   MLE_SE <-  MLE_par *0
  } else {
    MLE_SE <-sqrt(diag(solve(MLE_estimates$hessian)));
  }
}
fitAICC = 2* knep -2* (-MLE_estimates$value) + (2*knep*(knep+1) )/( ndatafitted - knep -1)
```

```
print(sprintf('MScur  = %f , loglik = %f , AICC= %f  ', NMS_seq, -MLE_estimates$value, fitAICC  ))



#if (-MLE_estimates$value > temploglik ) {
 # temploglik <- -MLE_estimates$value
#  flagms  <- NMS_seq
#}

# Standard error
#

resvector=c(flagms,NMS_seq, MLE_estimates$convergence, -
MLE_estimates$value,fitAICC,MLE_SE,MLE_par)

names(resvector) <-  matnames

# if ( -MLE_estimates$value > temploglik ) {
#  temploglik <- -MLE_estimates$value
#  fitAICC

#save( fitAICC, file =
paste("~/Anet_MEV_NUS/mv_rcode/mlepat_mevac_var_1994_QSdI_ALLP_tempaicc.RData",
sep=""))
# save(MLE_estimates, file =
paste("~/Anet_MEV_NUS/mv_rcode/mlepat_mevac_var_1994_QSdI_ALLP_full.RData", sep=""))
#}

###################
#check if we alrea
return(resvector) #return results from optimizer to main function
```

```
} #finish function for each scenario

###################################
#main progr
###################################
#main program
###################################
#tstart=proc.time(); #capture current time to measure duration of process



#%%%%%%%%%%%%%%%%%%%%%%%%%%%%%%%%%%%
#data from Ginsberg 1952
#virus and lung damage sampled at different times
#position/index for the different variables
#The palette with black:
#cbbPalette <- c( "#E69F00", "#009E73",  "#0072B2", "#D55E00", "#CC79A7")
# To use for fills, add

# To use for line and point colors, add
#scale_colour_manual(values=cbPalette)

temploglik <-  -Inf
flagms  <- 0

namevar = c('S','I','A','V')
# Initial conditions from the previous model fit
iS0 <-   c( 3828,  3905, 2456, 4053,   5487,  3342,   3876 )
iI0 <-   c( 0, 0, 0, 0, 0 , 0, 0)
iA0 <-   c(  36.5,  1.1, 1.2, 74.7,  2.6,67.4, 3.8 )
iV0 <-   c(  1.1*10^-5 ,  2.5*10^-5,1.0*10^-5, 1.0*10^-6, 9.9*10^-5, 3.0*10^-6,  1.0*10^-4 )
```

```
mcnames  <- c(  '15U','46U','55U','67U','40V','43V','55V')

#  Biological rates of each macaques
#Read csv file containing theestimarted parameter values from th previous work
estparamvac <- read.csv("estparamvac.csv", header = TRUE, sep = ",")
# view and check data
estparamvac




cat("red data from mvdata csv")
mesacmv <- read.csv("mvdata.csv", header = TRUE, sep = ",")
"show data from csv"
mesacmv

unique(mesacmv$ID)

min(mesacmv$MV.specific.T.cells )

mesacmv$IDM <- (mesacmv$ID)
mesacmv$IDM <-0
mesacmv$IDM <- as.numeric(mesacmv$IDM)
"start looping mc id"

for (ni in 1:7) {
  "befoe which output"
  mesacmv$ID== mcnames[ni]
  "which output"
  which(mesacmv$ID== mcnames[ni] )
  selidmw <- which(mesacmv$ID== mcnames[ni] )
```

```
  selidmw <-as.numeric(selidmw)
  "numeric selidmw"
  selidmw
  "before"
  mesacmv$IDM[selidmw]
  mesacmv$IDM[selidmw] <- ni
  "after"
  mesacmv$IDM[selidmw]
}
mesacmv$IDM <- as.numeric(mesacmv$IDM)
"create numerical newID for monkeys"
mesacmv$IDM

####################################

cat("red data from mevrnacsv")
mevrnadata <- read.csv("mevrnadatalin12.csv", header = TRUE, sep = ",")
"show data from mevrnadata csv"
mevrnadata



mevrnadata$IDM <- mevrnadata$ID
mevrnadata$IDM <-0
mevrnadata$IDM <- as.numeric(mevrnadata$IDM)
"start looping mc id"

for (ni in 1:7) {
  "befoe which output"
  mevrnadata$ID== mcnames[ni]
  "which output"
  which(mevrnadata$ID== mcnames[ni] )
```

```
  selidmw <- which(mevrnadata$ID== mcnames[ni] )
  selidmw <-as.numeric(selidmw)
  "numeric selidmw"
  selidmw
  "before"
  mevrnadata$IDM[selidmw]
  mevrnadata$IDM[selidmw] <- ni
  "after"

}
mevrnadata$IDM <- as.numeric(mevrnadata$IDM)
"create numerical newID for monkeys"
mevrnadata$IDM



###########################################
# ivlmev1994

cat("red data from ivlmev1994 csv")
ivlmev <- read.csv("ivlmev1994.csv", header = TRUE, sep = ",")
ivlmev
EID <- unique(ivlmev$eid)

for ( itc in 1: length(EID) ) {

  assign(paste0("fmes_ivl_", EID[itc]),  ivlmev$ivl[ which(ivlmev$eid == EID[itc] & ivlmev$cens == 0  )
])
  assign(paste0("mes_day_", EID[itc]),  ivlmev$day[ which(ivlmev$eid == EID[itc] & ivlmev$cens == 0
& ivlmev$day >= 0 ) ])
  assign(paste0("mes_lod_", EID[itc]),  ivlmev$lod[ which(ivlmev$eid == EID[itc] & ivlmev$cens == 0 &
ivlmev$day >= 0  ) ])
```

```
  assign(paste0("mes_ivl_0_", EID[itc]),  ivlmev$ivl[ which(ivlmev$eid == EID[itc] & ivlmev$cens == 0
& ivlmev$day == 0  ) ])

  assign(paste0("mes_ivl_", EID[itc]),  ivlmev$ivl[ which(ivlmev$eid == EID[itc] & ivlmev$cens == 0 &
ivlmev$day >= 0  ) ])


}


mes_day_1

n_days_ivl_1 <- length(mes_day_1)

n_days_ivl_10 <- length(mes_day_10)


#ivlmevsub <- subset (ivlmev, ivlmev$eid <=1000 & ivlmev$cens == 0)

#ivlmevsub0 <- subset (ivlmev, ivlmev$eid <=1000 & ivlmev$cens == 0  & ivlmev$day > 0)


ivlmevsub <- subset (ivlmev, ivlmev$eid != 1001 &ivlmev$eid !=1002 & ivlmev$cens == 0)

ivlmevsub0 <- subset (ivlmev, ivlmev$eid != 1001 & ivlmev$eid !=1002 & ivlmev$cens == 0  &
ivlmev$day > 0)

#


cat(  "Data 1994")


data94 <- data.frame(  TCID = c( mes_day_10*0+10^4,   mes_day_10*0+10^3,mes_day_10*0+10^2,
mes_day_10*0+10^1, mes_day_1*0+10^0)  ,

              mes =  c( mes_ivl_10000, mes_ivl_1000, mes_ivl_100, mes_ivl_10, mes_ivl_1  ) ,

              t =   c(  mes_day_10, mes_day_10, mes_day_10, mes_day_10,   mes_day_1 ) )




data94
```

```
cat("ndatafitted ")

ndatafitted <-  length(mes_day_1)+ 4*length(mes_day_10)
ndatafitted


t_ivl_1  <- as.matrix( mes_day_1 );
t_ivl_10  <- as.matrix( mes_day_10 );

cat("t_ivl_1 t_ivl_10 ")
t_ivl_1

t_ivl_10

##############
n_days_ivl_1 <- length(t_ivl_1)
n_days_ivl_10 <- length(t_ivl_10)


m_ivl_10000  <- as.matrix(  mes_ivl_10000  );
m_lod_10000  <- as.matrix(  mes_lod_10000  );
m_ivl_1000  <- as.matrix(  mes_ivl_1000  );
m_lod_1000  <- as.matrix(  mes_lod_1000  );
m_ivl_100  <- as.matrix(  mes_ivl_100  );
m_lod_100  <- as.matrix(  mes_lod_100  );
m_ivl_10  <- as.matrix(  mes_ivl_10  );
m_lod_10  <- as.matrix(  mes_lod_10  );
m_ivl_1  <- as.matrix(  mes_ivl_1  );
m_lod_1  <- as.matrix(  mes_lod_1  );

###################################
```

```
#  parnames=c('A_10000','V0_10000','V0_1000','V0_100','V0_10','V0_1','q','s')

#parnames=c('A0','V0_10000','qs','td','b','k','q','s','r','p','fi' )

defparnames = c('qs','td','b','di','k','q','s','d','r','p','c' )

# parmdl=c('qs','td','b','k','q','s','r','p','fi' )

Spos=1; Ipos=2; Apos=3; Vpos=4;

eturmerror <- 10^100

atolv=1e-14; rtolv=1e-14; #tolerances for ODE solver

n_monkeys = 7
eps=1e-15;# epsilon for log

#####################################################



####################
########################################

#pdf(paste("~/Anet_MEV_NUS/mv_rcode/testMLEincoculum".pdf", sep=""),, paper="a4r")

#par(mfrow=c(3,1), oma=c(1,1,1), mar=c(2,1,1))
```

```
#bounds on initial conditions
#bounds for parameters improves solver convergence
IV_10000_low=10^-16 ; IV_10000_high= 0.8; #for V0
IV_1000_low=1e-16; IV_1000_high=  0.3; #
IV_100_low=1e-16; IV_100_high=  0.3 ; #
IV_10_low=1e-16; IV_10_high= 0.3; #
IV_1_low=1e-16; IV_1_high= 0.3   ; #
IV_01_low=1e-16; IV_01_high=0.3     ; #

L_10000_low=3906 ; L_10000_high= 3907; #for L0
S0_low=1 ; S0_high=10^7; #for S0

R_low=1e-10; R_high=10^6; #for rna

A_10000_low=1 ;   A_10000_high= 200; #for V0
A_1000_low=1 ;   A_1000_high= 200 ; #for V0
A_100_low=1 ;   A_100_high=200 ; #for V0
A_10_low=1 ;   A_10_high= 200 ; #for V0
A_1_low=1 ;   A_1_high= 200 ; #for V0


q_low= 1.11 ; q_high= 1.119 ;
s_low= 0.0001 ; s_high= 0.25 ;

#bounds on parameters, all is in units o

qs_low= 0.028 ; qs_high= 0.029 ;
td_low=  2.6 ; td_high=2.7;
```

```
b_low= 0.01 ; b_high= 1 ;

di_low= 0.5 ; di_high=0.5;

k_low= 0.0007 ; k_high=0.05;

d_low= 1/40 ; d_high= 1/40  ;
r_low= 0.016 ; r_high= 0.017 ;
p_low=0.001; p_high= 0.025 ;
c_low= 3 ; c_high= 3 ;
fi_low=100; fi_high=10000;

#vector of parameter names and lower/upper bounds
# parnames=c('A0','V0_10000','qs','td','fi' )

# parnames=c('td','b','k','q','s','r','p','fi' )

#parnames=c('A_10000','A_1000','A_100','A_10','A_1','V0_10000','V0_1000','V0_100','V0_10','V0_1'
,'qs','td','b','k','q','s','r','p','fi' )

#lb=c(A0_low, IV_10000_low,  qs_low, td_low, fi_low)
# ub = c (A0_high, IV_10000_high,  qs_high , td_high, fi_high)

lb=c( A_10000_low,A_1000_low, A_100_low, A_10_low, A_1_low,
IV_10000_low,IV_1000_low,IV_100_low,IV_10_low,IV_1_low, b_low,k_low,  s_low, p_low );

ub = c(A_10000_high,A_1000_high,A_100_high,A_10_high,A_1_high,
IV_10000_high,IV_1000_high,IV_100_high,IV_10_high,IV_1_high, b_high,k_high,  s_high, p_high);
```

```
parnames=c('A_10000','A_1000','A_100','A_10','A_1','V0_10000','V0_1000','V0_100','V0_10','V0_1','
b','k','s','p')


parnames_se =
c('se_A_10000','se_A_1000','se_A_100','se_A_10','se_A_1','se_V0_10000','se_V0_1000','se_V0_100'
,'se_V0_10','se_V0_1','se_b','se_k','se_s','se_p')


rect_lbub <- cbind(lb, ub)

length(parnames )
length(rect_lbub)/2
length(lb )


######################################
# multistart
#number of multisart


real.time.start=date(); #get current time to measure length of optimization for each strain
tstart=proc.time(); #capture current time
print(sprintf('Optimization started at %s ',real.time.start))

############### unbiformn multistart
IV_10000_ug <-   seq(IV_10000_low, IV_10000_high, length=NMS )
IV_1000_ug <-   seq(IV_1000_low, IV_1000_high, length=NMS )
IV_100_ug <-   seq(IV_100_low, IV_100_high, length=NMS )
IV_10_ug <-   seq(IV_10_low, IV_10_high, length=NMS )
```

```
IV_1_ug <-   seq(IV_1_low, IV_1_high, length=NMS )

IV_01_ug <-   seq(IV_01_low, IV_01_high, length=NMS )


L_10000_ug <-   seq(L_10000_low, L_10000_high, length=NMS )

A_10000_ug <-   seq(A_10000_low, A_10000_high, length=NMS )

A_1000_ug <-   seq(A_1000_low, A_1000_high, length=NMS )

A_100_ug <-   seq(A_100_low, A_100_high, length=NMS )

A_10_ug <-   seq(A_10_low, A_10_high, length=NMS )

A_1_ug <-   seq(A_1_low, A_1_high, length=NMS )


R_ug <-   seq(R_low, R_high, length=NMS )


qs_ug <-   seq(qs_low, qs_high, length=NMS )

td_ug <-   seq(td_low, td_high, length=NMS )

b_ug <-   seq(b_low, b_high, length=NMS )

k_ug <-   seq(k_low, k_high, length=NMS )

q_ug <-   seq(q_low, q_high, length=NMS )

s_ug <-   seq(qs_low, qs_high, length=NMS )

r_ug <-   seq(r_low, r_high, length=NMS )

p_ug <-   seq(p_low, p_high, length=NMS )

fi_ug <-   seq(fi_low, fi_high, length=NMS )



para_guess_ug <-  rbind(   A_1000_ug,  A_100_ug,  A_10_ug,  A_1_ug,
IV_10000_ug,IV_1000_ug,IV_100_ug,IV_10_ug,IV_1_ug,b_ug,k_ug, q_ug, s_ug,   p_ug);


para_guess_ug <- unname(para_guess_ug)



#######################################################

#######random sampling from uniform
```

```
IV_10000_rug <-   runif(NMS, min = IV_10000_low, IV_10000_high )
IV_1000_rug <-    runif(NMS, min = IV_1000_low, IV_1000_high )
IV_100_rug <-    runif(NMS, min = IV_100_low, IV_100_high )
IV_10_rug <-    runif(NMS, min =  IV_10_low, IV_10_high )
IV_1_rug <-    runif(NMS, min = IV_1_low, IV_1_high )
IV_01_rug <-    runif(NMS, min = IV_01_low, IV_01_high )

L_10000_rug <-    runif(NMS, min =  L_10000_low, L_10000_high )
A_10000_rug <-    runif(NMS, min =  A_10000_low, A_10000_high)
A_1000_rug <-    runif(NMS, min =  A_1000_low, A_1000_high)
A_100_rug <-    runif(NMS, min =  A_100_low, A_100_high)
A_10_rug <-    runif(NMS, min =  A_10_low, A_10_high)
A_1_rug <-    runif(NMS, min =  A_1_low, A_1_high)

R_rug <-    runif(NMS, min = R_low, R_high )

qs_rug <-    runif(NMS, min = qs_low, qs_high )
td_rug <-    runif(NMS, min = td_low, td_high )
b_rug <-    runif(NMS, min = b_low, b_high )
k_rug <-    runif(NMS, min = k_low, k_high )
q_rug <-    runif(NMS, min = q_low, q_high )
s_rug <-  runif(NMS, min = qs_low, qs_high )
r_rug <-  runif(NMS, min = r_low, r_high )
p_rug <-  runif(NMS, min = p_low, p_high )
fi_rug <-    runif(NMS, min =  fi_low, fi_high )

para_guess_rug <-    rbind( A_10000_rug,  A_1000_rug,  A_100_rug,  A_10_rug,  A_1_rug,
IV_10000_rug,IV_1000_rug,IV_100_rug,IV_10_rug,IV_1_rug,b_rug,k_rug,  s_rug, p_rug);
```

```
para_guess_rug <- unname(para_guess_rug)



###########
###### LHS latin hypercube

para_guess_lhs <- lhs(NMS, rect_lbub)
para_guess_lhs[1,]

para_guess_lhs <-  t(para_guess_lhs )

######################################################
#######################################

#looping for each  of the 7 macaqies
ii <-  2
##################################################
# for ( ii in 1:7){
"ii"
ii
"mes day pi"
mesacmv$Day

mcqii <- unique(mesacmv$ID[ which(mesacmv$IDM== ii)])
mcqii

day_tlym <- mesacmv$Day[ which(mesacmv$IDM== ii & !is.na(mesacmv$Total.lymphocytes)  ) ]

enddpo <- max(mesacmv$Day)+ 1
times   <- seq(0, enddpo, by = 1)
```

```
t_al <- as.matrix(times );

"mes day pi day_tlym"
day_tlym

mes_tlym <- mesacmv$Total.lymphocytes[ which(mesacmv$IDM== ii &
!is.na(mesacmv$Total.lymphocytes)  ) ]

"mes pi mes_tlym"
mes_tlym

"length mes_tlym"
length(mes_tlym)

mes_tcell <- mesacmv$MV.specific.T.cells[ which(mesacmv$IDM== ii &
!is.na(mesacmv$MV.specific.T.cells )  ) ]

mes_AB <- mesacmv$MV.specific.antibodies[ which(mesacmv$IDM== ii &
!is.na(mesacmv$MV.specific.antibodies)  ) ]

day_ivl <- mesacmv$Day[ which(mesacmv$IDM== ii & !is.na(mesacmv$Viral.load)  ) ]

idmonkeydata <- subset(mesacmv, mesacmv$IDM== ii )

idxday_ivl <- which( !is.na(idmonkeydata$Viral.load)  )
```

```
mes_ivl <- mesacmv$Viral.load[ which(mesacmv$IDM== ii & !is.na(mesacmv$Viral.load)  ) ]


mes_tlym0 <-  mes_tlym[1]

fmes_tlym <-  mes_tlym

mes_tlym <-  mes_tlym

mes_tcell0 <-  mes_tcell[1]

fmes_tcell <-  mes_tcell

#log 0 issues

#reference

# https://aosmith.rbind.io/2018/09/19/the-log-0-
problem/#:~:text=The%20log%20transformation%20tends%20to%20feature%20prominently%20for,
This%20isn%E2%80%99t%20necessarily%20an%20incorrect%20thing%20to%20do.


HMA <-  min(fmes_tcell[fmes_tcell>0])/2

mes_tcell <-  mes_tcell

simday_tlym <- day_tlym

day_tlym <- day_tlym

n_days_tlym <- length(day_tlym)

(n_days_tlym)

length(day_tlym)

length(mes_tlym)

length(mes_tcell)



mes_ivl0 <-  mes_ivl[1]

fmes_ivl <-  mes_ivl

HMV <-  min(fmes_ivl[fmes_ivl>0])/2

mes_ivl <-  mes_ivl

day_ivl <- day_ivl

n_days_ivl <- length(mes_ivl)
```

```
length(n_days_ivl)

length(day_ivl)

length(mes_ivl)


cbdata_mes <-  append ( mes_tlym, mes_tcell )


cbdata_mes <-  append ( cbdata_mes, mes_ivl )



################################################


# Initial conditions



yicA =iA0[ii]


#Define parameter values

resvec  <-   c( qs = estparamvac$qs[ii] ,

             td =  estparamvac$td[ii] ,

             b  = estparamvac$b[ii],

             di  = estparamvac$di[ii],

             k = estparamvac$k[ii],

             q  = estparamvac$q[ii],

             s  = estparamvac$s[ii],

             d  = estparamvac$d[ii],

             r  = estparamvac$r[ii],

             p  = estparamvac$p[ii],

             c  = estparamvac$c[ii],

             fi  = estparamvac$fi[ii]  )


 mesacmv$MV.specific.T.cellsFS <-   mesacmv$MV.specific.T.cells * estparamvac$fi
```

```
# RUN ODE

# #####################

yic    = c( S =iS0[ii], I = iI0[ii], A =iA0[ii], V =iV0[ii]   )

"Initialcondition"

yic

#   odestack=try(ode( func = mvequations,  y = yic, times = times, parms=resvec, method ="daspk")); #runs the ODE equations

odestack=try(lsoda( yic, times, mvequations_di,parms=resvec, atol=atolv,rtol=rtolv)); #runs the ODE equations

if (length(odestack)==1) {cat('!!unresolvable integrator error - triggering early return from optimizer!!'); return(1e10) } #catching errors that might happen during fitting

print(sprintf(' end run ode out' ))

Smlin=odestack[match(mes_day_10,odestack[,1]),Spos+1]; #extract values for virus load at time points corresponding to experimental measurements

Imlin=odestack[match( mes_day_10,odestack[,1]),Ipos+1]; #extract values for virus load at time points corresponding to experimental measurements

Amlin=odestack[match(mes_day_10 ,odestack[,1]),Apos+1]; #extract values for virus load at time points corresponding to experimental measurements

#   print(sprintf(' prep loglik ' ))

simes_tlym  = (Smlin + Imlin + Amlin)

simes_tcell  =  Amlin / estparamvac$fi[ii]

simes_ivl  =  odestack[match( mes_day_10,odestack[,1]),Vpos+1]; #extract values for virus load at time points corresponding to experimental measurements

omes_tlym  <- mes_tlym

omes_tcell  <- mes_tcell
```

```
omes_ivl  <- mes_ivl_10000


mdata <- data.frame(  mtlym = omes_tlym ,
                  mtcell = omes_tcell*1000  ,
                  tm = day_tlym )




####################
####################################################


loglb=log10(lb); logub=log10(ub); #fit in log space


# initial guess


para_guess_mb = (lb+ub)/2


#para_guess_MS =   (para_guess_ug)
para_guess_MS =   (para_guess_rug)
 #para_guess_MS =   (para_guess_lhs)


knep <-  length(para_guess_MS[,1])
knep
length(para_guess_rug)


length(loglb)
length(logub)
length(parnames)


#selection of one start
para_guess_MS[,1]
```

```
##########################################################
#para_guess = (   (para_guess_MS[,1 ]))


#para_guess =   c(   1,
#                 0.3,
#                0.1,
#               0.1,
#              0.05,
#             iS0[ii],
#            1.307430e-02,
#           4.2,
#          0.05 ,
#         0.01 ,
#        0.5  ,
#       0.5,
#      0.1 ,
#     0.01  )


# Number of estimated parameters
#knep <-  length(para_guess)
#knep
#para_guess = log10(   (para_guess))


#names(para_guess)=parnames; #assign names to parameters


#MLE_estimates <- optim(fn=log_like,                  # Likelihood function
 #                  #par= log10(MLE_estimates$par),                  # Initial guess
  #                 par= para_guess,                  # Initial guess
   #               lower = loglb,        # Lower bound on parameters
```

```
   #              upper = logub,          # Upper bound on parameters
    #            hessian=TRUE,
     #          #method = "BFGS",
      #        method = "L-BFGS-B")
       #      #control = list(   reltol= 1e-20, lmm=31,  factr = 1e-20))
#

####################
#####################
################################################
#matrix containing all results
length(parnames_se)+length(parnames)

matnames=c('FLAGLL','MS','CONVG','LOGLIK','AICC',parnames_se,parnames)
resmatrix=matrix(0,nrow=NMS,ncol=length(matnames))
colnames(resmatrix) <- matnames;
# resmatrix




reslist <- NULL
parallel.comp = 1 ; #turn on or off parallel computing -
node.num=NMS; #number of sockets/nodes to use for parallel computing
node.type=1; #choose socket/node type. 1 for SOCK (can be run locally), 2 for MPI



cat(  "start psim ")

load("~/Anet_MEV_NUS/mv_rcode/mle_MShpc_1994FXICdi_res_46u_050_eeflo8xxLL_ww.RData")

#save(MSLL_resmatrix, file = paste("mle_MShpc_1994FX_ICdi_pres.RData", sep=""))
```

```
cat("best fit ")
MSLL_resmatrix



TCID <- c(10^4, 10^3, 10^2, 10^1, 1)
V0 <- c(4.853541e-01, 1.610022e-01, 1.700542e-01, 8.458384e-02, 4.845793e-03)
A0 <- c(9.260934e+00 , 8.088699e+00, 8.678568e+00, 7.442463e+00, 5.404490e+00)
TTP <- c(7, 9, 9, 9, 13)

# Create a data frame
data <- data.frame(TCID, V0, A0, TTP)

# Calculate correlation matrix
cor_matrix <- cor(data)

# Plot correlation matrix
library(corrplot)
##########################################




###############################################

corrplot(cor_matrix)

corrplot.mixed( cor_matrix,
        lower = "number",
        upper = "circle",
```

```
            tl.col = "black")




# Perform linear regression
lm_model <- lm(A0 ~ V0)


# Get regression coefficients and p-value
coef <- coef(lm_model)[2]
p_val <- summary(lm_model)$coefficients[2, 4]
cor_test <- cor.test(A0, V0)
p_val<- cor_test$p.value
coef <- as.numeric(cor_test$estimate)

# Create a data frame
data <- data.frame(V0, A0)

# Plot linear regression using ggplot2
V0A0 <-  ggplot(data, aes(x = V0, y = A0,  color=as.factor(TCID))) +
  geom_point(size=2) +
  scale_x_log10( breaks= round(V0,3)) +
  scale_y_continuous( breaks= round(A0,2)) +
    geom_smooth(method = "lm", se = FALSE, color = "black") +
  labs(x = "V0", y = "A0") +
  labs(color = "TCID=")+
  scale_fill_manual(values=cbbPalette)+
    annotate("text", x = 0.1, y =11,
           label = paste("Coefficient =", round(coef, 2)),)+
  annotate("text", x = 0.1, y = 10 ,
```

```
        label= paste("p =", round(as.numeric(format(p_val, scientific = TRUE)),3), sep=" "),
        color="black")+
  theme(legend.position = "none")+
  theme(axis.text = element_text(size = 12, face = "bold"),
      axis.title = element_text(size = 14, face = "bold"))



# Perform linear regression
lm_model <- lm(A0~ TCID)

lm_growth  <- lm_model
summary(lm_growth )

lrnexp_value


#   0.56725

coef <- coef(lm_growth )[2]
p_val <- summary(lm_growth )$coefficients[2, 4]

# -0.48618



# Get the summary of the model
summary_lm <- summary(  lm_growth )

# Extract the standard errors of coefficients

coefficients <- summary_lm$coefficients[, "Estimate"]
```

```
coeff_standard_errors <- summary_lm$coefficients[, "Std. Error"]

# Extract the intercept and slope (coefficient of x)
intercept <- coefficients[1]
slope <- coefficients[2]

lr_exp <- slope*  c(5,9,10) +intercept

lr_exp

df_lr_exp = data.frame(categ = as.factor(c(1,1,1)),tt = c(5,9,10), yy= lr_exp)

# Extract the standard deviations for intercept and slope
intercept_std_dev <- coeff_standard_errors[1]
slope_std_dev <- coeff_standard_errors[2]

# Print the values and standard deviations
cat("Intercept:", intercept, " (Standard Deviation:", intercept_std_dev, ")\n")
cat("Slope (Coefficient of x):", slope, " (Standard Deviation:", slope_std_dev, ")\n")




# Get regression coefficients and p-value
coef <- coef(lm_model)[2]
p_val <- summary(lm_model)$coefficients[2, 4]
cor_test <- cor.test(A0, log10(TCID))
p_val<- cor_test$p.value
coef <- as.numeric(cor_test$estimate)

# Create a data frame
```

```
data <- data.frame(TCID, A0)

data$categ <- fct_rev( as.factor( data$TCID))


# Plot linear regression using ggplot2
T0A0 <-    ggplot(  data, aes(x = TCID, y = A0,  color=categ   ) ) +
  # geom_line(  size =1.25) +
  geom_point(  aes(  color=categ, shape=categ),     size=2) +
  geom_smooth(method = "lm", se = FALSE, color = "black") +
  scale_shape_manual( values=c( 18, 8,17,19,  15)) +
  # scale_fill_manual(values=cbbPalette) +
  scale_color_manual(values=cbbPalette) +
  labs( shape = bquote(  TCID[50] ~ "="), color = bquote(  TCID[50] ~ "=") ) +
  scale_x_log10( breaks= TCID) +
  scale_y_log10( ) +
  labs(x =bquote(  TCID[50]), y = "A0") +
  # labs(color = bquote(  TCID[50]))+
  # scale_fill_manual(values=cbbPalette)+
  annotate("text", x = 100, y =10,
           label = paste("C =", round(coef, 2),"*"))+
  #annotate("text", x = 10, y = 0.9,
  #        legend.position = "top" ,
  #      legend.direction = "horizontal",  # Display the legend horizontally
  #               label= paste("p =", round(as.numeric(format(p_val, scientific = TRUE)),3), sep=" "),
  #     color="black")+
  theme(axis.text = element_text(size = 12, face = "bold"),
        axis.title = element_text(size = 12, face = "bold")) +
  theme(legend.position = "none")+
  theme(axis.text.x = element_text(angle=45, vjust=1, hjust=1))+
  scale_y_continuous(limits = c(5,10),breaks=c(5,6,7,8,9,10 ))
```

```
# Perform linear regression
lm_model <- lm(V0~ TCID)
summary(lm_model)

lm_growth  <- lm_model
summary(lm_growth )

lrnexp_value


#   0.56725

coef <- coef(lm_growth )[2]
p_val <- summary(lm_growth )$coefficients[2, 4]

# -0.48618


# Get the summary of the model
summary_lm <- summary(  lm_growth )
```

```
# Extract the standard errors of coefficients

coefficients <- summary_lm$coefficients[, "Estimate"]
coeff_standard_errors <- summary_lm$coefficients[, "Std. Error"]

# Extract the intercept and slope (coefficient of x)
intercept <- coefficients[1]
slope <- coefficients[2]

lr_exp <- slope*  c(5,9,10) +intercept

lr_exp

df_lr_exp = data.frame(categ = as.factor(c(1,1,1)),tt = c(5,9,10), yy= lr_exp)

# Extract the standard deviations for intercept and slope
intercept_std_dev <- coeff_standard_errors[1]
slope_std_dev <- coeff_standard_errors[2]

# Print the values and standard deviations
cat("Intercept:", intercept, " (Standard Deviation:", intercept_std_dev, ")\n")
cat("Slope (Coefficient of x):", slope, " (Standard Deviation:", slope_std_dev, ")\n")



# Get regression coefficients and p-value
coef <- coef(lm_model)[2]
p_val <- summary(lm_model)$coefficients[2, 4]
cor_test <- cor.test(V0, log10(TCID))
p_val<- cor_test$p.value
coef <- as.numeric(cor_test$estimate)
```

```
# Create a data frame
data <- data.frame(TCID, V0 )


data$categ <- fct_rev( as.factor( data$TCID))



# Plot linear regression using ggplot2
T0V0 <-    ggplot(  data, aes(x = TCID, y = V0,  color=categ   ) ) +
                # geom_line(  size =1.25) +
                geom_point(  aes(  color=categ, shape=categ),     size=2) +
                    geom_smooth(method = "lm", se = FALSE, color = "black") +
                   scale_shape_manual( values=c( 18, 8,17,19,  15)) +
               # scale_fill_manual(values=cbbPalette) +
                scale_color_manual(values=cbbPalette) +
                labs( shape = bquote(  TCID[50] ~ "="), color = bquote(  TCID[50] ~ "=") ) +
                scale_x_log10( breaks= c(1,10,10^2,10^3,10^4   )) +
  scale_y_log10( ) +
  labs(x =bquote(  TCID[50]), y = "V0") +
  # labs(color = bquote(  TCID[50]))+
  # scale_fill_manual(values=cbbPalette)+
  annotate("text", x = 10, y =1.5,
          label = paste("C =", round(coef, 2), "*"))+
  #annotate("text", x = 10, y = 0.9,
   #        legend.position = "top" ,
    #      legend.direction = "horizontal",  # Display the legend horizontally
     #              label= paste("p =", round(as.numeric(format(p_val, scientific = TRUE)),3), sep=" "),
      #     color="black")+
  theme(axis.text = element_text(size = 12, face = "bold"),
```

```
    axis.title = element_text(size = 12, face = "bold"))+
  theme(legend.position = "none") +
  theme(axis.text.x = element_text(angle=45, vjust=1, hjust=1))



cor_test <- cor.test(TTP, log10(TCID))
p_val<- cor_test$p.value
coef <- as.numeric(cor_test$estimate)

# Create a data frame
data <- data.frame(TCID, TTP)

# Plot linear regression using ggplot2
T0TTP <-  ggplot(data, aes(x = TCID, y = TTP,  color=as.factor(TCID))) +
  geom_point(size=2) +
  scale_x_log10( breaks= TCID) +
  geom_smooth(method = "lm", se = FALSE, color = "black") +
  labs(x =bquote(  TCID[50]), y = "TTP") +
  labs(color = bquote(  TCID[50]))+
  scale_fill_manual(values=cbbPalette)+
  annotate("text", x = 100, y =15,
         label = paste("Coefficient =", round(coef, 2)))+
  annotate("text", x = 100, y = 14,
         label= paste("p =", round(as.numeric(format(p_val, scientific = TRUE)),3), sep=" "),
         color="black")+
  theme(legend.position = "none")+
  theme(axis.text = element_text(size = 12, face = "bold"),
      axis.title = element_text(size = 14, face = "bold"))
```

```
cor_test <- cor.test(TTP, V0)
p_val<- cor_test$p.value
coef <- as.numeric(cor_test$estimate)

# Create a data frame
data <- data.frame(V0, TTP)

# Plot linear regression using ggplot2
V0TTP <-  ggplot(data, aes(x = V0 ,y = TTP,  color=as.factor(TCID))) +
  geom_point(size=2) +
  scale_x_log10( breaks= round(V0,3)) +
  geom_smooth(method = "lm", se = FALSE, color = "black") +
  labs(x = "V0", y = "TTP") +
  labs(color = "TCID=")+
  scale_fill_manual(values=cbbPalette)+
  annotate("text", x = .1, y =15,
           label = paste("Coefficient =", round(coef, 2)))+
  annotate("text", x = .1, y = 14,
           label= paste("p =", round(as.numeric(format(p_val, scientific = TRUE)),3), sep=" "),
           color="black")+
  theme(legend.position = "none")+
  theme(axis.text = element_text(size = 12, face = "bold"),
        axis.title = element_text(size = 14, face = "bold"))




cor_test <- cor.test(TTP, A0)

# Perform linear regression
```

```
lm_model <- lm(TTP~ A0)


lm_growth  <- lm_model
summary(lm_growth )



lrnexp_value


#   0.56725

coef <- coef(lm_growth )[2]
p_val <- summary(lm_growth )$coefficients[2, 4]

# -0.48618


# Get the summary of the model
summary_lm <- summary(  lm_growth )

# Extract the standard errors of coefficients

coefficients <- summary_lm$coefficients[, "Estimate"]
coeff_standard_errors <- summary_lm$coefficients[, "Std. Error"]

# Extract the intercept and slope (coefficient of x)
intercept <- coefficients[1]
slope <- coefficients[2]
```

```
lr_exp <- slope*  c(5,9,10) +intercept

lr_exp

df_lr_exp = data.frame(categ = as.factor(c(1,1,1)),tt = c(5,9,10), yy= lr_exp)

# Extract the standard deviations for intercept and slope
intercept_std_dev <- coeff_standard_errors[1]
slope_std_dev <- coeff_standard_errors[2]

# Print the values and standard deviations
cat("Intercept:", intercept, " (Standard Deviation:", intercept_std_dev, ")\n")
cat("Slope (Coefficient of x):", slope, " (Standard Deviation:", slope_std_dev, ")\n")



cor_test <- cor.test(TTP, A0)
p_val<- cor_test$p.value
coef <- as.numeric(cor_test$estimate)

# Create a data frame
data <- data.frame(A0, TTP,TCID)

data$categ <- fct_rev( as.factor( data$TCID))


# Plot linear regression using ggplot2
A0TTP <-    ggplot(  data, aes(x = A0 ,y = TTP,  color=categ   ) ) +
  # geom_line(  size =1.25) +
  geom_point(  aes(  color=categ, shape=categ),     size=2) +
  geom_smooth(method = "lm", se = FALSE, color = "black") +
```

```
scale_shape_manual( values=c( 18, 8,17,19,  15)) +
# scale_fill_manual(values=cbbPalette) +
scale_color_manual(values=cbbPalette) +
labs( shape = bquote(  TCID[50] ~ "="), color = bquote(  TCID[50] ~ "=") ) +
labs(x ="A0", y = "Day of peak viremia") +
# labs(color = bquote(  TCID[50]))+
# scale_fill_manual(values=cbbPalette)+
annotate("text", x = 7, y =14,
        label = paste("C =", round(coef, 2),"*"))+
#annotate("text", x = 10, y = 0.9,
#        legend.position = "top" ,
#      legend.direction = "horizontal",  # Display the legend horizontally
#               label= paste("p =", round(as.numeric(format(p_val, scientific = TRUE)),3), sep=" "),
#    color="black")+
theme(legend.position = "none")+
theme(axis.text = element_text(size = 12, face = "bold"),
     axis.title = element_text(size = 12, face = "bold")) +
theme(axis.text.x = element_text(angle=45, vjust=1, hjust=1))+
scale_y_continuous(limits = c(6,14),breaks=c(7,9,13 ))+
scale_x_continuous(limits = c(5,10),breaks=c(5,6,7,8,9,10 ))
```

```
mdlfrnCOR <- plot_grid(  T0V0, T0A0, V0A0,T0TTP,V0TTP, A0TTP,
                    labels = c("A", "B","C","D", "E","F"),
                    align="h", ncol=3,
                    label_size = 12)

mdlfrnCOR
######################

#####################
"savecombinefigdynamics"
save_plot(paste("f-mv-model_1994_hpcMS_corTTP.pdf", sep=""), mdlfrnCOR ,  ncol = 3, nrow = 2)



mdlfrnCOR <- plot_grid(  T0V0, T0A0 , A0TTP,
                   labels = c("a", "b","c"),
                   align="h", ncol=2,
                   label_size = 11)

mdlfrnCOR
######################

#####################
"savecombinefigdynamics"
save_plot(paste("f-mv-model_1994_Fig3.pdf", sep=""), mdlfrnCOR ,  ncol = 2, nrow = 2)



# Save the ggplot as an EPS file
ggsave("Fig3.eps", plot = mdlfrnCOR, device = "eps", width = 4.5, height = 5, units = "in")
save_plot(paste("Fig3.pdf", sep=""), mdlfrnCOR ,  ncol = 2, nrow = 2)
```

```
############# PLots
####################################################


A0_10000 <-   MSLL_resmatrix["A_10000"]
A0_1000 <-   MSLL_resmatrix["A_1000"];
A0_100 <-   MSLL_resmatrix["A_100"];
A0_10 <-   MSLL_resmatrix["A_10"];
A0_1 <-   MSLL_resmatrix["A_1"];



V_10000  <- MSLL_resmatrix["V0_10000"];
V_1000  <-   MSLL_resmatrix["V0_1000"];
V_100  <-   MSLL_resmatrix["V0_100"];
V_10  <-   MSLL_resmatrix["V0_10"];
V_1  <-   MSLL_resmatrix["V0_1"];

L0_10000 <-   3906# MSLL_resmatrix["L_10000"];

#Define parameter value

allparsode <-   c( 0.028, # MSLL_resmatrix["qs"] ,
                2.6 , #MSLL_resmatrix["td"],
                MSLL_resmatrix["b"] ,
                0.5 ,
                MSLL_resmatrix["k"],
                1.11, # MSLL_resmatrix["q"],
                MSLL_resmatrix["s"],
                0.025,
                0.016, #  MSLL_resmatrix["r"],
                MSLL_resmatrix["p"]  ,
```

```
3 )

names(allparsode)=defparnames; #assign names to parameters

cat("best para ")
allparsode

# RUN ODE
#print(sprintf(' start run ode ' ))
INC <- c(  L0_10000-A0_10000, 0, A0_10000,  V_10000 );
names(INC)= namevar; #assign names to parameters

cat("best INC 10000")
INC

odestack=NULL
IVmlin = NULL
#  odestack=try(ode( func = mvequations,  y = INC, times = t_al, parms=allparsode, method ="daspk")); #runs the ODE equations

odestack=try(lsoda( INC, t_al,mvequations_di,parms=allparsode, atol=atolv,rtol=rtolv)); #runs the ODE equations
if (length(odestack)==1) {cat('!!unresolvable integrator error - triggering early return from optimizer!!'); return(eturmerror) } #catching errors that might happen during fitting
IVmlin =odestack[match( t_ivl_10 ,odestack[,1]),Vpos+1]; #extract values for virus load at time points corresponding to experimental measurements

cat("sim V 10000 ")
IVmlin
```

#plot(t_ivl_10,   m_ivl_10000)

if (sum(is.na(lVmlin))>0) {cat('!!ODE 10000fail VL NA return!!'); return(eturmerror) } #catching errors that might happen during fitting

if (sum(is.infinite(10^lVmlin))>0) {cat('!!ODE 10000fail VL INF correct!!'); return(eturmerror) } # lVmlin[which(lVmlin>300)] <-300 } #catching errors that might happen during fitting

Smlin=odestack[match(t_al,odestack[,1]),Spos+1]; #extract values for virus load at time points corresponding to experimental measurements

Imlin_10000=odestack[match(t_al ,odestack[,1]),Ipos+1]; #extract values for virus load at time points corresponding to experimental measurements

Imlin=odestack[match(t_al ,odestack[,1]),Ipos+1]; #extract values for virus load at time points corresponding to experimental measurements

Amlin_10000=odestack[match(t_al ,odestack[,1]),Apos+1]; #extract values for virus load at time points corresponding to experimental measurements

Lmscal_10000 = Smlin + Imlin + Amlin_10000

#if (Lmscal_10000[length(Lmscal_10000)]< 2000) {cat('!!ODE 10000faIL LOW TLYM'); return(eturmerror) }

lVmlong_10000 =odestack[match(t_al ,odestack[,1]),Vpos+1]; #extract values for virus load at time points corresponding to experimental measurements

correct lod

if (lVmlin[1] <= 0.3) {lVmlin[1] <- 0.3 }

if (lVmlin[7] <= 0.3) {lVmlin[7] <- 0.3 }

if (lVmlin[8] <= 0.3) {lVmlin[8] <- 0.3 }

RSS_IV   <-   log(10^lVmlin) - log(10^m_ivl_10000)

loglik_IV_10000_bis <- -(n_days_ivl_10 /2)* log((2*pi/n_days_ivl_10) * (t(RSS_IV) %*% RSS_IV)) -(n_days_ivl_10 /2)

```
cat("loglik_IV_10000_bis ")

loglik_IV_10000_bis




IVmlin_10000  <- IVmlin




INC <- c(  L0_10000-A0_1000, 0, A0_1000,  V_1000 );

names(INC)= namevar; #assign names to parameters

odestack=NULL

IVmlin = NULL



#  odestack=try(ode( func = mvequations,  y = INC, times = t_al, parms=allparsode, method ="daspk")); #runs the ODE equations

odestack=try(lsoda( INC, t_al,mvequations_di,parms=allparsode, atol=atolv,rtol=rtolv)); #runs the ODE equations

if (length(odestack)==1) {cat('!!unresolvable integrator error - triggering early return from optimizer!!'); return(eturmerror) } #catching errors that might happen during fitting

IVmlin =odestack[match( t_ivl_10 ,odestack[,1]),Vpos+1]; #extract values for virus load at time points corresponding to experimental measurements

if (sum(is.na(IVmlin))>0) {cat('!!ODE 10000fail VL NA return!!'); return(eturmerror) } #catching errors that might happen during fitting

if (sum( is.infinite(10^IVmlin))>0) {cat('!!ODE 10000fail VL INF correct!!');  return(eturmerror) } # IVmlin[ which( IVmlin>300  ) ] <-300 } #catching errors that might happen during fitting


Smlin=odestack[match(t_al,odestack[,1]),Spos+1]; #extract values for virus load at time points corresponding to experimental measurements

Imlin=odestack[match( t_al ,odestack[,1]),Ipos+1]; #extract values for virus load at time points corresponding to experimental measurements

Imlin_1000=odestack[match( t_al ,odestack[,1]),Ipos+1]; #extract values for virus load at time points corresponding to experimental measurements
```

```
Amlin_1000 =odestack[match(t_al ,odestack[,1]),Apos+1]; #extract values for virus load at time points corresponding to experimental measurements

Lmscal_1000 = Smlin + Imlin + Amlin_1000

IVmlong_1000 =odestack[match( t_al ,odestack[,1]),Vpos+1]; #extract values for virus load at time points corresponding to experimental measurements


# correct lod

if (IVmlin[1] <= 0.3) {IVmlin[1]  <- 0.3 }

if (IVmlin[2] <= 0.3) {IVmlin[2]  <- 0.3 }

if (IVmlin[8] <= 0.3) {IVmlin[8]  <- 0.3 }


RSS_IV     <-    log(  10^IVmlin )  - log(  10^m_ivl_1000 )

loglik_IV_1000_bis  <-  -(n_days_ivl_10 /2)*  log(  (2*pi/n_days_ivl_10) * ( t( RSS_IV ) %*% RSS_IV )  ) -(n_days_ivl_10 /2)


cat("loglik_IV_1000_bis ")

loglik_IV_1000_bis


IVmlin_1000  <- IVmlin



INC <- c(  L0_10000-A0_100, 0, A0_100,  V_100 );


names(INC)= namevar; #assign names to parameters

odestack=NULL

IVmlin = NULL



#  odestack=try(ode( func = mvequations,  y = INC, times = t_al, parms=allparsode, method ="daspk")); #runs the ODE equations
```

```
odestack=try(lsoda( INC, t_al,mvequations_di,parms=allparsode, atol=atolv,rtol=rtolv)); #runs the ODE equations

if (length(odestack)==1) {cat('!!unresolvable integrator error - triggering early return from optimizer!!'); return(eturmerror) } #catching errors that might happen during fitting

IVmlin =odestack[match( t_ivl_10 ,odestack[,1]),Vpos+1]; #extract values for virus load at time points corresponding to experimental measurements

if (sum(is.na(IVmlin))>0) {cat('!!ODE 10000fail VL NA return!!'); return(eturmerror) } #catching errors that might happen during fitting

if (sum( is.infinite(10^IVmlin))>0) {cat('!!ODE 10000fail VL INF correct!!');  return(eturmerror) } # IVmlin[ which( IVmlin>300  ) ] <-300 } #catching errors that might happen during fitting


Smlin=odestack[match(t_al,odestack[,1]),Spos+1]; #extract values for virus load at time points corresponding to experimental measurements

Imlin=odestack[match( t_al ,odestack[,1]),Ipos+1]; #extract values for virus load at time points corresponding to experimental measurements

Imlin_100=odestack[match( t_al ,odestack[,1]),Ipos+1]; #extract values for virus load at time points corresponding to experimental measurements


Amlin_100=odestack[match(t_al ,odestack[,1]),Apos+1]; #extract values for virus load at time points corresponding to experimental measurements

Lmscal_100 = Smlin + Imlin + Amlin_100


IVmlong_100 =odestack[match( t_al ,odestack[,1]),Vpos+1]; #extract values for virus load at time points corresponding to experimental measurements


# correct lod

if (IVmlin[1] <= 0.3) {IVmlin[1]  <- 0.3 }

if (IVmlin[2] <= 0.3) {IVmlin[2]  <- 0.3 }

if (IVmlin[8] <= 0.3) {IVmlin[8]  <- 0.3 }


RSS_IV     <-    log(  10^IVmlin )  - log(  10^m_ivl_100 )

loglik_IV_100_bis  <-  -(n_days_ivl_10 /2)*  log(  (2*pi/n_days_ivl_10) * ( t( RSS_IV ) %*% RSS_IV )  ) - (n_days_ivl_10 /2)


cat("loglik_IV_100_bis ")
```

```
loglik_IV_100_bis

IVmlin_100  <- IVmlin

INC <- c(  L0_10000-A0_10, 0, A0_10,  V_10 );

names(INC)= namevar; #assign names to parameters

odestack=NULL

IVmlin = NULL

#  odestack=try(ode( func = mvequations,  y = INC, times = t_al, parms=allparsode, method ="daspk")); #runs the ODE equations

odestack=try(lsoda( INC, t_al,mvequations_di,parms=allparsode, atol=atolv,rtol=rtolv)); #runs the ODE equations

if (length(odestack)==1) {cat('!!unresolvable integrator error - triggering early return from optimizer!!'); return(eturmerror) } #catching errors that might happen during fitting

IVmlin =odestack[match( t_ivl_10 ,odestack[,1]),Vpos+1]; #extract values for virus load at time points corresponding to experimental measurements

if (sum(is.na(IVmlin))>0) {cat('!!ODE 10000fail VL NA return!!'); return(eturmerror) } #catching errors that might happen during fitting

if (sum( is.infinite(10^IVmlin))>0) {cat('!!ODE 10000fail VL INF correct!!');  return(eturmerror) } # IVmlin[ which( IVmlin>300  ) ] <-300 } #catching errors that might happen during fitting

Smlin=odestack[match(t_al,odestack[,1]),Spos+1]; #extract values for virus load at time points corresponding to experimental measurements

Imlin_10 =odestack[match( t_al ,odestack[,1]),Ipos+1]; #extract values for virus load at time points corresponding to experimental measurements

Imlin=odestack[match( t_al ,odestack[,1]),Ipos+1]; #extract values for virus load at time points corresponding to experimental measurements

Amlin_10 =odestack[match(t_al ,odestack[,1]),Apos+1]; #extract values for virus load at time points corresponding to experimental measurements
```

```
Lmscal_10 = Smlin + Imlin + Amlin_10


IVmlong_10 =odestack[match( t_al ,odestack[,1]),Vpos+1]; #extract values for virus load at time points corresponding to experimental measurements


# correct lod

if (IVmlin[1] <= 0.3) {IVmlin[1]  <- 0.3 }

if (IVmlin[2] <= 0.3) {IVmlin[2]  <- 0.3 }

if (IVmlin[8] <= 0.3) {IVmlin[8]  <- 0.3 }


RSS_IV     <-    log(  10^IVmlin )  - log(  10^m_ivl_10 )

loglik_IV_10_bis  <-  -(n_days_ivl_10 /2)*  log(  (2*pi/n_days_ivl_10) * ( t( RSS_IV ) %*% RSS_IV )  ) - (n_days_ivl_10 /2)


cat("n_days_ivl_10 ")

n_days_ivl_10


cat("loglik_IV_10_bis ")

loglik_IV_10_bis


IVmlin_10  <- IVmlin




INC <- c(  L0_10000-A0_1, 0, A0_1,  V_1 );


names(INC)= namevar; #assign names to parameters

odestack=NULL

IVmlin = NULL
```

```
#  odestack=try(ode( func = mvequations,  y = INC, times = t_al, parms=allparsode, method ="daspk")); #runs the ODE equations

odestack=try(lsoda( INC, t_al,mvequations_di,parms=allparsode, atol=atolv,rtol=rtolv)); #runs the ODE equations

if (length(odestack)==1) {cat('!!unresolvable integrator error - triggering early return from optimizer!!'); return(eturmerror) } #catching errors that might happen during fitting

lVmlin =odestack[match( t_ivl_1 ,odestack[,1]),Vpos+1]; #extract values for virus load at time points corresponding to experimental measurements


cat("sim V1 ")

lVmlin




if (sum(is.na(lVmlin))>0) {cat('!!ODE 10000fail VL NA return!!'); return(eturmerror) } #catching errors that might happen during fitting

if (sum( is.infinite(10^lVmlin))>0) {cat('!!ODE 10000fail VL INF correct!!');   lVmlin[ which( lVmlin>300 ) ] <-300 } #catching errors that might happen during fitting


Smlin=odestack[match(t_al,odestack[,1]),Spos+1]; #extract values for virus load at time points corresponding to experimental measurements

Imlin_1=odestack[match( t_al ,odestack[,1]),Ipos+1]; #extract values for virus load at time points corresponding to experimental measurements


Imlin=odestack[match( t_al ,odestack[,1]),Ipos+1]; #extract values for virus load at time points corresponding to experimental measurements

Amlin_1 =odestack[match(t_al ,odestack[,1]),Apos+1]; #extract values for virus load at time points corresponding to experimental measurements

Lmscal_1 = Smlin + Imlin + Amlin_1


lVmlong_1 =odestack[match( t_al ,odestack[,1]),Vpos+1]; #extract values for virus load at time points corresponding to experimental measurements


# correct lod

if (lVmlin[1] <= 0.3) {lVmlin[1]  <- 0.3 }

if (lVmlin[2] <= 0.3) {lVmlin[2]  <- 0.3 }
```

```
if (IVmlin[3] <= 0.3) {IVmlin[3]  <- 0.3 }

RSS_IV     <-    log(  10^IVmlin )  - log(  10^m_ivl_1 )
loglik_IV_1_bis  <-  -(n_days_ivl_1 /2)*  log(  (2*pi/n_days_ivl_1) * ( t( RSS_IV ) %*% RSS_IV )  ) -
(n_days_ivl_1 /2)

cat("n_days_ivl_1 ")
n_days_ivl_1

cat("loglik_IV_1_bis ")
loglik_IV_1_bis

IVmlin_1  <- IVmlin

odestack=NULL
IVmlin = NULL

loglik_bis <-  loglik_IV_10000_bis   +  loglik_IV_1000_bis   +loglik_IV_100_bis   + loglik_IV_10_bis   +
loglik_IV_1_bis

cat("loglik_bis  ")
loglik_bis

cat("loglik_ref  ")
MSLL_resmatrix["LOGLIK"]
#####################

##########################################################
```

```
#MLE <- data.table(param = parnames,
#               estimates = MLE_par,
#              sd = MLE_SE,
#             aicc =  MLE_par*0+ datasim$AICC[1],
#            loglik = MLE_par*0-MLE_estimates$value )

#             TABMLE  <-  kable(MLE)

#            kable(data.table(MSLL_resmatrix))



datafit  <- data.frame(  TCID = c( t_al*0+10^4,   t_al*0+10^3,t_al*0+10^2, t_al*0+10^1, t_al*0+10^0)
,
                    X50. =  c( IVmlong_10000,IVmlong_1000, IVmlong_100, IVmlong_10, IVmlong_1  ) ,
                    #mes =  c( m_ivl_10000, m_ivl_1000, m_ivl_100, m_ivl_10, m_ivl_1  ) ,
                    tinf = c(Imlin_10000, Imlin_1000,Imlin_100,Imlin_10,Imlin_1 ),
                    tlym = c(Lmscal_10000, Lmscal_1000,Lmscal_100,Lmscal_10,Lmscal_1 ),
                    tcell = c(Amlin_10000, Amlin_1000, Amlin_100, Amlin_10, Amlin_1    ),
                    t =   c(  t_al, t_al, t_al, t_al,   t_al   ) )


##########################################################
############################################

 ggplot() +
 #geom_ribbon(aes(ymin = X5., ymax = X95.), fill = "orange", alpha = 0.35) +
 geom_line(data = data94, aes(x = t, y = mes, color=as.factor(TCID) ),  size =0.25) +
 geom_line(data = datafit, aes(x = t, y = X50., color=as.factor(TCID)) , size =1.5, alpha=0.75) +
```

```
  geom_point(data = data94,  aes(x = t, y = mes, color=as.factor(TCID)),     size=1.5) +
  #geom_line(mapping = aes( y = si,m_ivl),linetype = "dashed",size=2, color = "blue") +
  labs(x = "Days post infection", y =bquote(  "Log" ~  TCID[50] ~  "/" ~  10^6 ~  "PBMC"))+
  ggtitle("Infectious virus ")  +
  #annotate(geom="text", x=25, y=4, label=mcqii, color="black")+
  labs(color = "Inoculum TCID=")+
  scale_fill_manual(values=cbbPalette)+
  scale_x_continuous(limits=c(0, 30), breaks=c(0,  3,  5,  7,  9, 11, 14, 18, 25, 70) )+
  annotate(geom="text", x=5, y=4,size=4,
          label= paste("MLE= ",round(as.numeric(MSLL_resmatrix["LOGLIK"] ),2), sep=""),
          color="black")+
  annotate(geom="text", x=20, y=4,size=4,
          label= paste("AICc= ",round(as.numeric(MSLL_resmatrix["AICC"] ),2), sep=""),
          color="black")+
  theme(legend.position = "none")


fig_ICDI


############################




##################################


library(forcats)
```

```
data94$categ <- fct_rev( as.factor( data94$TCID))

datafit$categ <- fct_rev( as.factor(  datafit$TCID))




FIG_ICDI <-  ggplot(  data = datafit, aes(x = t, y = X50.,  color=categ, fill=categ,   group=categ ) ) +
  geom_line(  size =1.25) +
  geom_point(data = data94,  aes(x = t, y = mes,   color=categ, fill=categ, shape=categ),     size=2) +
  scale_shape_manual( values=c( 18, 8,17,19,  15)) +
  scale_fill_manual(values=cbbPalette) +
  scale_color_manual(values=cbbPalette) +
  labs( shape = bquote(  TCID[50] ~ "="), fill = bquote(  TCID[50] ~ "="), color = bquote(  TCID[50] ~
"=") ) +
  #geom_ribbon(aes(ymin = X5., ymax = X95.), fill = "orange", alpha = 0.35) +
  #geom_point(data = data94,  aes(x = t, y = mes,   color=as.factor(TCID)),   size=2) +
  #geom_line(mapping = aes( y = si,m_ivl),linetype = "dashed",size=2, color = "blue") +
  labs(x = "Days post MV infection", y =bquote(  "Log" ~  TCID[50] ~  "/" ~  10^6 ~  "PBMC"))+
  ggtitle("Infectious viremia ")  +
  #annotate(geom="text", x=25, y=4, label=mcqii, color="black")+
  theme(legend.position="bottom")   +
  theme(legend.text = element_text(colour="black",  face="bold")) +
  geom_hline(yintercept = 0.3, linetype = "dotdash", color = "darkgray", size=1) +
  scale_x_continuous( limits=c(0, 30),  breaks=c(0,  3,  5,  7,  9,  13,  17, 20,25,30 ))+
  scale_y_continuous(limits = c(0,4),breaks=c(0.3,1,2,  3,3.4,  4 ))+
  annotate(geom="text", x=8, y=4,size=2.5,
         label= paste("B2: MLE=",round(as.numeric(MSLL_resmatrix["LOGLIK"] ),2), sep=""),
         color="black")+
  annotate(geom="text", x=23, y=4,size=2.5,
         label= paste("AICc=",round(as.numeric(MSLL_resmatrix["AICC"] ),2), sep=""),
         color="black")
#+
```

```
#+  FIG_ICDIGA <-

  ggplot(  data = datafit, aes(x = t, y = X50.,  color=categ, fill=categ,   group=categ ) ) +
  geom_line(  size =1.25) +
  geom_point(data = data94,  aes(x = t, y = mes,   color=categ, fill=categ, shape=categ),     size=2) +
  scale_shape_manual( values=c( 18, 8,17,19,  15)) +
  scale_fill_manual(values=cbbPalette) +
  scale_color_manual(values=cbbPalette) +
  labs( shape = bquote(  TCID[50] ~ "="), fill = bquote(  TCID[50] ~ "="), color = bquote(  TCID[50] ~
"=") ) +
  #geom_ribbon(aes(ymin = X5., ymax = X95.), fill = "orange", alpha = 0.35) +
  #geom_point(data = data94,  aes(x = t, y = mes,   color=as.factor(TCID)),   size=2) +
  #geom_line(mapping = aes( y = si,m_ivl),linetype = "dashed",size=2, color = "blue") +
  labs(x = "Days post MV infection", y =bquote(  "Log" ~  TCID[50] ~  "/" ~  10^6 ~  "PBMC"))+
  ggtitle("Infectious viremia ")  +
  #annotate(geom="text", x=25, y=4, label=mcqii, color="black")+
  theme(legend.position="bottom")   +
  theme(legend.text = element_text(colour="black",  face="bold")) +
  geom_hline(yintercept = 0.3, linetype = "dotdash", color = "darkgray", size=1) +
  scale_x_continuous( limits=c(0, 30),  breaks=c(0,  3,  5,  7,  9,  13,  17, 20,25,30 ))+
  scale_y_continuous(limits = c(0,4),breaks=c(0.3,1,2,  3,3.4,  4 ))

#+

#  theme(legend.position = "none")

FIG_ICDIlg <-   FIG_ICDI + theme(legend.position = "none")
```

```
FIG_ICDIp <-  ggplot(  data = datafit, aes(x = t, y = X50.,  color=categ, fill=categ,   group=categ ) ) +
  geom_line(  size =1.25) +
  geom_point(data = data94,  aes(x = t, y = mes,   color=categ, fill=categ, shape=categ),     size=2) +
  scale_shape_manual( values=c( 18, 8,17,19,  15)) +
  scale_fill_manual(values=cbbPalette) +
  scale_color_manual(values=cbbPalette) +
  labs( shape = bquote( "Initial" ~  TCID[50] ~ "="), fill = bquote(   "Initial" ~ TCID[50] ~ "="), color = 
bquote(  "Initial" ~  TCID[50] ~ "=") ) +
  #geom_ribbon(aes(ymin = X5., ymax = X95.), fill = "orange", alpha = 0.35) +
  #geom_point(data = data94,  aes(x = t, y = mes,   color=as.factor(TCID)),   size=2) +
  #geom_line(mapping = aes( y = si,m_ivl),linetype = "dashed",size=2, color = "blue") +
  labs(x = "Days post MV infection", y =bquote(  "Log" ~  TCID[50] ~  "/" ~  10^6 ~  "PBMC"))+
  ggtitle("Infectious viremia ")  +
  #annotate(geom="text", x=25, y=4, label=mcqii, color="black")+
  theme(legend.position="top")   +
  theme(legend.text = element_text(colour="black",  face="bold")) +
  geom_hline(yintercept = 0.3, linetype = "dotdash", color = "darkgray", size=1) +
  scale_x_continuous( limits=c(0, 30),  breaks=c(0,  3,  5,6,  7,  9, 11, 13, 14, 17, 18,20,25,30 ))+
  scale_y_continuous(limits = c(0,3.5),breaks=c(0.3,1,2,  3,3.4,  4 ))

datafit$FV <- datafit$tcell * datafit$X50. / ( MSLL_resmatrix["s"]  + datafit$X50. )

datafit$KIA <- datafit$tcell * datafit$tinf  *  MSLL_resmatrix["k"]

FIG_KIAp <-  ggplot(  data = datafit, aes(x = t, y = KIA,  color=categ, fill=categ,   group=categ ) ) +
  geom_line(  size =1) +
  #geom_point( aes(  shape=categ),     size=2) +
```

```
  scale_shape_manual( values=c( 18, 8,17,19,  15)) +
  scale_fill_manual(values=cbbPalette) +
  scale_color_manual(values=cbbPalette) +
  labs( shape = bquote(  TCID[50] ~ "="), fill = bquote(  TCID[50] ~ "="), color = bquote(  TCID[50] ~
"=") ) +
  #geom_ribbon(aes(ymin = X5., ymax = X95.), fill = "orange", alpha = 0.35) +
  #geom_point(data = data94,  aes(x = t, y = mes,   color=as.factor(TCID)),   size=2) +
  #geom_line(mapping = aes( y = si,m_ivl),linetype = "dashed",size=2, color = "blue") +
  labs(x = " ", y =bquote(  "Magnitude of kIA"))+
  ggtitle("MV control by T cell killing")  +
  #annotate(geom="text", x=25, y=4, label=mcqii, color="black")+
  theme(legend.position="none")   +
  theme(legend.text = element_text(colour="black",  face="bold")) +
  # geom_hline(yintercept = 0.3, linetype = "dotdash", color = "darkgray", size=1.25, alpha=0.5) +
  scale_x_continuous( limits=c(0, 30),  breaks=c(0,  3,  5,6,  7,  9, 11, 13, 14, 17, 18,20,25,30 )) +
#  scale_y_continuous(  breaks=c(   100,400,600, 1000, 1100) )
scale_y_log10( limits=c(0.005, 1200),  breaks=c(0.01,  1,10, 100,500, 1000) )


#scale_y_continuous(limits = c(0,4),breaks=c(0.3,1,2,  3,3.4,  4 ))+



fig_KIA <-  ggplot(  data = datafit, aes(x = t, y = KIA,  color=categ, fill=categ,   group=categ ) ) +
  geom_line(  size =1) +
  geom_point( aes(  shape=categ),     size=2) +
  scale_shape_manual( values=c( 18, 8,17,19,  15)) +
  scale_fill_manual(values=cbbPalette) +
  scale_color_manual(values=cbbPalette) +
  labs( shape = bquote(  TCID[50] ~ "="), fill = bquote(  TCID[50] ~ "="), color = bquote(  TCID[50] ~
"=") ) +
  #geom_ribbon(aes(ymin = X5., ymax = X95.), fill = "orange", alpha = 0.35) +
  #geom_point(data = data94,  aes(x = t, y = mes,   color=as.factor(TCID)),   size=2) +
  #geom_line(mapping = aes( y = si,m_ivl),linetype = "dashed",size=2, color = "blue") +
```

```
  labs(x = NULL, y =bquote(  "Magnitude of kIA"))+
  ggtitle("MV control by T cell killing")  +
  #annotate(geom="text", x=25, y=4, label=mcqii, color="black")+
  theme(legend.position="none")   +
  theme(legend.text = element_text(colour="black",  face="bold")) +
  # geom_hline(yintercept = 0.3, linetype = "dotdash", color = "darkgray", size=1.25, alpha=0.5) +
  scale_x_continuous( limits=c(0, 30),  breaks=c(0,  3,  5,6,  7,  9, 11, 13, 14, 17, 18,20,25,30 )) +
  #  scale_y_continuous(  breaks=c(   100,400,600, 1000, 1100) )
  scale_y_log10( limits=c(0.005, 1200),  breaks=c(0.01,  1,10, 100, 1000) )

#scale_y_continuous(limits = c(0,4),breaks=c(0.3,1,2,  3,3.4,  4 ))+



fig_KIA  <-   fig_KIA  + theme(
  legend.text = element_text(size = 11),  # Legend font size
  legend.title = element_text(size = 11), # Legend title font size
  plot.title = element_text(size = 11),   # Plot title font size
  axis.title.x = element_text(size = 11), # X-axis label font size
  axis.title.y = element_text(size = 11)  # Y-axis label font size
)



FIG_FV <-  ggplot(  data = datafit, aes(x = t, y = FV,  color=categ, fill=categ,   group=categ ) ) +
  geom_line(  size =1) +
  geom_point( aes(  shape=categ),     size=2) +
  scale_shape_manual( values=c( 18, 8,17,19,  15)) +
  scale_fill_manual(values=cbbPalette) +
  scale_color_manual(values=cbbPalette) +
  labs( shape = bquote(  TCID[50] ~ "="), fill = bquote(  TCID[50] ~ "="), color = bquote(  TCID[50] ~
"=") ) +
  #geom_ribbon(aes(ymin = X5., ymax = X95.), fill = "orange", alpha = 0.35) +
```

```
  #geom_point(data = data94,  aes(x = t, y = mes,   color=as.factor(TCID)),   size=2) +
  #geom_line(mapping = aes( y = si,m_ivl),linetype = "dashed",size=2, color = "blue") +
  labs(x = "Days post MV infection", y =bquote(  "Magnitude of Af(V)"))+
  ggtitle("T cell response activation ")  +
  #annotate(geom="text", x=25, y=4, label=mcqii, color="black")+
  theme(legend.position="none")   +
  theme(legend.text = element_text(colour="black",  face="bold")) +
 # geom_hline(yintercept = 0.3, linetype = "dotdash", color = "darkgray", size=1.25, alpha=0.5) +
  scale_x_continuous( limits=c(0, 30),  breaks=c(0,  3,  5,6,  7,  9, 11, 13, 14, 17, 18,20,25,30 )) +
scale_y_log10(limits=c(10^-5, 10^4) )

  #scale_y_continuous(limits = c(0,4),breaks=c(0.3,1,2,  3,3.4,  4 ))+

#+
#+

  fig_Lp <-   ggplot(  data = datafit, aes(x = t,  y = tlym,  color=categ, fill=categ,   group=categ ) ) +
  geom_line(  size =1) +
  #  geom_point( aes( color=categ, fill=categ, shape=categ),     size=2) +
    scale_shape_manual( values=c( 18, 8,17,19,  15)) +
    scale_fill_manual(values=cbbPalette) +
    scale_color_manual(values=cbbPalette) +
    labs( shape = bquote(  TCID[50] ~ "="), fill = bquote(  TCID[50] ~ "="), color = bquote(  TCID[50] ~
"=") ) +
  #geom_ribbon(aes(ymin = X5., ymax = X95.), fill = "orange", alpha = 0.35) +
  #geom_point(data = data94,  aes(x = t, y = mes,   color=as.factor(TCID)),   size=2) +
  #geom_line(mapping = aes( y = si,m_ivl),linetype = "dashed",size=2, color = "blue") +
  labs(x = "Days post MV infection", y =  expression( "Cells/" ~ mu ~ "l")  )+
    ggtitle(" Total lymphocyte count ") +
    #annotate(geom="text", x=25, y=4, label=mcqii, color="black")+
  theme(legend.position="bottom")   +
```

```
theme(legend.text = element_text(colour="black",  face="bold")) +
scale_x_continuous( limits=c(0, 30),  breaks=c(0,  3,  5,6,  7,  9, 11, 13, 14, 17, 18,20,25,30 ))+
 scale_y_continuous(limits=c(2000, 8000), breaks=c(2000,4000,6000,8000 ))+
   theme(legend.position = "none")



fig_L <-   ggplot(  data = datafit, aes(x = t,  y = tlym,  color=categ, fill=categ,   group=categ ) ) +
  geom_line(  size =1) +
   geom_point( aes( color=categ, fill=categ, shape=categ),     size=2) +
  scale_shape_manual( values=c( 18, 8,17,19,  15)) +
  scale_fill_manual(values=cbbPalette) +
  scale_color_manual(values=cbbPalette) +
  labs( shape = bquote(  TCID[50] ~ "="), fill = bquote(  TCID[50] ~ "="), color = bquote(  TCID[50] ~
"=") ) +
  #geom_ribbon(aes(ymin = X5., ymax = X95.), fill = "orange", alpha = 0.35) +
  #geom_point(data = data94,  aes(x = t, y = mes,   color=as.factor(TCID)),   size=2) +
  #geom_line(mapping = aes( y = si,m_ivl),linetype = "dashed",size=2, color = "blue") +
  labs(x = NULL, y =  expression( "Cells/" ~ mu ~ "l")  )+
  ggtitle(" Total lymphocyte count ") +
  #annotate(geom="text", x=25, y=4, label=mcqii, color="black")+
  theme(legend.position="bottom")   +
  theme(legend.text = element_text(colour="black",  face="bold")) +
  scale_x_continuous( limits=c(0, 30),  breaks=c(0,  3,  5,6,  7,  9, 11, 13, 14, 17, 18,20,25,30 ))+
  scale_y_continuous(limits=c(2000, 8000), breaks=c(2000,4000,6000,8000 ))+
  theme(legend.position = "none")


fig_L  <-   fig_L  + theme(
  legend.text = element_text(size = 11),  # Legend font size
```

```
    legend.title = element_text(size = 11), # Legend title font size
    plot.title = element_text(size = 11),   # Plot title font size
    axis.title.x = element_text(size = 11), # X-axis label font size
    axis.title.y = element_text(size = 11)  # Y-axis label font size
  )

"Generatefigure A"
max(  datafit$tcell )



fig_A <-    ggplot(  data = datafit, aes(x = t,  y = tcell,  color=categ, fill=categ,   group=categ ) ) +
  geom_line(  size =1) +
  geom_point( aes( color=categ, fill=categ, shape=categ),     size=2) +
  scale_shape_manual( values=c( 18, 8,17,19,  15)) +
  scale_fill_manual(values=cbbPalette) +
  scale_color_manual(values=cbbPalette) +
  labs( shape = bquote(  TCID[50] ~ "="), fill = bquote(  TCID[50] ~ "="), color = bquote(  TCID[50] ~
"=") ) +
  #geom_ribbon(aes(ymin = X5., ymax = X95.), fill = "orange", alpha = 0.35) +
  #geom_point(data = data94,  aes(x = t, y = mes,   color=as.factor(TCID)),   size=2) +
  #geom_line(mapping = aes( y = si,m_ivl),linetype = "dashed",size=2, color = "blue") +
  labs(x = "Days post MV infection", y =  expression("IFN-" ~ gamma ~ "spot-forming cells/" ~ mu ~ "l")
)+
  ggtitle("MV-specific T cells") +
  #annotate(geom="text", x=25, y=4, label=mcqii, color="black")+
  theme(legend.position="bottom")   +
  theme(legend.text = element_text(colour="black",  face="bold")) +
  scale_x_continuous( limits=c(0, 30),  breaks=c(0,  3,  5,6,  7,  9, 11, 13, 14, 17, 18,20,25,30 ))+
  #scale_y_continuous(breaks=c(2000,4000,6000,8000 ))+
  scale_y_log10(limits=c(5, 10000),  breaks=c(10,100,300,1000,10000) )+
  theme(legend.position = "none")
```

```
fig_A <-   fig_A  + theme(
  legend.text = element_text(size = 11),  # Legend font size
  legend.title = element_text(size = 11), # Legend title font size
  plot.title = element_text(size = 11),   # Plot title font size
  axis.title.x = element_text(size = 11), # X-axis label font size
  axis.title.y = element_text(size = 11)  # Y-axis label font size
)


fig_A <-   fig_A  + theme(axis.title.y = element_text(size = 6))  # Adjust the size (12) as needed



 #+
  # scale_y_continuous(limits = c(0,4),breaks=c(0.3,1,2,  3,3.4,  4 ))+
  #theme(legend.position = "none")




mdlFV <- plot_grid( fig_A  ,  FIG_FV  , fig_L,
                  labels = c("a", "b", "c"),
                  align="h", ncol=2,
                  label_size = 11)

mdlFV

save_plot(paste("f-mv-data_1994_f4_AFVAL.pdf", sep=""), mdlFV,  ncol = 2, nrow = 2 )
```

```
# Save the ggplot as an EPS file
ggsave("Fig44.eps", plot = mdlFV , device = "eps", width = 4.5, height = 5, units = "in")



mdlFV <- plot_grid( fig_A  ,  FIG_FV  , FIG_KIA  , fig_L,
          labels = c("a", "b", "c", "d"),
          align="h", ncol=2,
          label_size = 11)

mdlFV

save_plot(paste("f-mv-data_1994_f4_KIAAFVAL.pdf", sep=""), mdlFV,  ncol = 2, nrow = 2 )



mdlALKIA <- plot_grid( fig_A  ,  fig_KIA  , fig_L,
          labels = c("a", "b", "c"),
          align="h", ncol=1,
          label_size = 11)

mdlALKIA

save_plot(paste("f-mv-data_1994_f4_ALKIA.pdf", sep=""), mdlALKIA,  ncol = 1, nrow = 3 )


# Save the ggplot as an EPS file
ggsave("Fig4.eps", plot = mdlALKIA , device = "eps", width = 5, height = 5, units = "in")
save_plot(paste("Fig4.pdf", sep=""), mdlALKIA,  ncol = 1, nrow = 3 )
```

```
########################################

A0_10000 <-   MSLL_resmatrix["A_10000"]
A0_1000 <-   MSLL_resmatrix["A_1000"];
A0_100 <-   MSLL_resmatrix["A_100"];
A0_10 <-   MSLL_resmatrix["A_10"];
A0_1 <-   MSLL_resmatrix["A_1"];



V_10000  <- MSLL_resmatrix["V0_10000"];
V_1000  <-   MSLL_resmatrix["V0_1000"];
V_100  <-   MSLL_resmatrix["V0_100"];
V_10  <-   MSLL_resmatrix["V0_10"];
V_1  <-   MSLL_resmatrix["V0_1"];

L0_10000 <-   3906# MSLL_resmatrix["L_10000"];

#Define parameter value

allparsode <-   c( 0.028, # MSLL_resmatrix["qs"] ,
              2.6 , #MSLL_resmatrix["td"],
              MSLL_resmatrix["b"] ,
              0.5 ,
              MSLL_resmatrix["k"],
              1.11, # MSLL_resmatrix["q"],
              MSLL_resmatrix["s"],
              0.025,
              0.016, #  MSLL_resmatrix["r"],
              MSLL_resmatrix["p"]  ,
              3 )
```

```
names(allparsode)=defparnames; #assign names to parameters

cat("best para ")
allparsode

# RUN ODE
#print(sprintf(' start run ode ' ))
INC <- c(  L0_10000-A0_10000, 0, A0_10000,  V_10000 );
names(INC)= namevar; #assign names to parameters

cat("best INC 10000")
INC

odestack=NULL
IVmlin = NULL
#  odestack=try(ode( func = mvequations,  y = INC, times = t_al, parms=allparsode, method
="daspk")); #runs the ODE equations

odestack=try(lsoda( INC, t_al,mvequations_di,parms=allparsode, atol=atolv,rtol=rtolv)); #runs the
ODE equations
if (length(odestack)==1) {cat('!!unresolvable integrator error - triggering early return from
optimizer!!'); return(eturmerror) } #catching errors that might happen during fitting
IVmlin =odestack[match( t_ivl_10 ,odestack[,1]),Vpos+1]; #extract values for virus load at time points
corresponding to experimental measurements

cat("sim V 10000 ")
IVmlin

#plot( t_ivl_10,   m_ivl_10000)
```

```
if (sum(is.na(IVmlin))>0) {cat('!!ODE 10000fail VL NA return!!'); return(eturmerror) } #catching errors that might happen during fitting

if (sum( is.infinite(10^IVmlin))>0) {cat('!!ODE 10000fail VL INF correct!!');  return(eturmerror) } # IVmlin[ which( IVmlin>300  ) ] <-300 } #catching errors that might happen during fitting



Smlin=odestack[match(t_al,odestack[,1]),Spos+1]; #extract values for virus load at time points corresponding to experimental measurements

Imlin=odestack[match( t_al ,odestack[,1]),Ipos+1]; #extract values for virus load at time points corresponding to experimental measurements

Amlin_10000=odestack[match(t_al ,odestack[,1]),Apos+1]; #extract values for virus load at time points corresponding to experimental measurements

Lmscal_10000 = Smlin + Imlin + Amlin_10000

#if (Lmscal_10000[length(Lmscal_10000)]< 2000) {cat('!!ODE 10000faIL LOW TLYM'); return(eturmerror) }


IVmlong_10000 =odestack[match( t_al ,odestack[,1]),Vpos+1]; #extract values for virus load at time points corresponding to experimental measurements


# correct lod

if (IVmlin[1] <= 0.3) {IVmlin[1]  <- 0.3 }

if (IVmlin[7] <= 0.3) {IVmlin[7]  <- 0.3 }

if (IVmlin[8] <= 0.3) {IVmlin[8]  <- 0.3 }


RSS_IV     <-    log( 10^IVmlin )  - log( 10^m_ivl_10000 )


loglik_IV_10000_bis  <-  -(n_days_ivl_10 /2)*  log(  (2*pi/n_days_ivl_10) * ( t( RSS_IV ) %*% RSS_IV ) ) -(n_days_ivl_10 /2)


cat("loglik_IV_10000_bis ")

loglik_IV_10000_bis
```

```
IVmlin_10000  <- IVmlin

INC <- c(  L0_10000-A0_1000, 0, A0_1000,  V_1000 );

names(INC)= namevar; #assign names to parameters

odestack=NULL

IVmlin = NULL

#  odestack=try(ode( func = mvequations,  y = INC, times = t_al, parms=allparsode, method ="daspk")); #runs the ODE equations

odestack=try(lsoda( INC, t_al,mvequations_di,parms=allparsode, atol=atolv,rtol=rtolv)); #runs the ODE equations

if (length(odestack)==1) {cat('!!unresolvable integrator error - triggering early return from optimizer!!'); return(eturmerror) } #catching errors that might happen during fitting

IVmlin =odestack[match( t_ivl_10 ,odestack[,1]),Vpos+1]; #extract values for virus load at time points corresponding to experimental measurements

if (sum(is.na(IVmlin))>0) {cat('!!ODE 10000fail VL NA return!!'); return(eturmerror) } #catching errors that might happen during fitting

if (sum( is.infinite(10^IVmlin))>0) {cat('!!ODE 10000fail VL INF correct!!');  return(eturmerror) } # IVmlin[ which( IVmlin>300  ) ] <-300 } #catching errors that might happen during fitting

Smlin=odestack[match(t_al,odestack[,1]),Spos+1]; #extract values for virus load at time points corresponding to experimental measurements

Imlin=odestack[match( t_al ,odestack[,1]),Ipos+1]; #extract values for virus load at time points corresponding to experimental measurements

Amlin_1000 =odestack[match(t_al ,odestack[,1]),Apos+1]; #extract values for virus load at time points corresponding to experimental measurements

Lmscal_1000 = Smlin + Imlin + Amlin_1000

IVmlong_1000 =odestack[match( t_al ,odestack[,1]),Vpos+1]; #extract values for virus load at time points corresponding to experimental measurements
```

```
# correct lod

if (IVmlin[1] <= 0.3) {IVmlin[1]  <- 0.3 }

if (IVmlin[2] <= 0.3) {IVmlin[2]  <- 0.3 }

if (IVmlin[8] <= 0.3) {IVmlin[8]  <- 0.3 }


RSS_IV     <-    log(  10^IVmlin )  - log(  10^m_ivl_1000 )

loglik_IV_1000_bis  <-  -(n_days_ivl_10 /2)*  log(  (2*pi/n_days_ivl_10) * ( t( RSS_IV ) %*% RSS_IV )  )
-(n_days_ivl_10 /2)


cat("loglik_IV_1000_bis ")

loglik_IV_1000_bis


IVmlin_1000  <- IVmlin



INC <- c(  L0_10000-A0_100, 0, A0_100,  V_100 );


names(INC)= namevar; #assign names to parameters

odestack=NULL

IVmlin = NULL



#  odestack=try(ode( func = mvequations,  y = INC, times = t_al, parms=allparsode, method
="daspk")); #runs the ODE equations

odestack=try(lsoda( INC, t_al,mvequations_di,parms=allparsode, atol=atolv,rtol=rtolv)); #runs the
ODE equations

if (length(odestack)==1) {cat('!!unresolvable integrator error - triggering early return from
optimizer!!'); return(eturmerror) } #catching errors that might happen during fitting

IVmlin =odestack[match( t_ivl_10 ,odestack[,1]),Vpos+1]; #extract values for virus load at time points
corresponding to experimental measurements

if (sum(is.na(IVmlin))>0) {cat('!!ODE 10000fail VL NA return!!'); return(eturmerror) } #catching errors
that might happen during fitting
```

```
if (sum( is.infinite(10^IVmlin))>0) {cat('!!ODE 10000fail VL INF correct!!');  return(eturmerror) } # IVmlin[ which( IVmlin>300  ) ] <-300 } #catching errors that might happen during fitting

Smlin=odestack[match(t_al,odestack[,1]),Spos+1]; #extract values for virus load at time points corresponding to experimental measurements

Imlin=odestack[match( t_al ,odestack[,1]),Ipos+1]; #extract values for virus load at time points corresponding to experimental measurements

Amlin_100=odestack[match(t_al ,odestack[,1]),Apos+1]; #extract values for virus load at time points corresponding to experimental measurements

Lmscal_100 = Smlin + Imlin + Amlin_100

IVmlong_100 =odestack[match( t_al ,odestack[,1]),Vpos+1]; #extract values for virus load at time points corresponding to experimental measurements

# correct lod

if (IVmlin[1] <= 0.3) {IVmlin[1]  <- 0.3 }

if (IVmlin[2] <= 0.3) {IVmlin[2]  <- 0.3 }

if (IVmlin[8] <= 0.3) {IVmlin[8]  <- 0.3 }

RSS_IV     <-    log(  10^IVmlin )  - log(  10^m_ivl_100 )

loglik_IV_100_bis  <-  -(n_days_ivl_10 /2)*  log(  (2*pi/n_days_ivl_10) * ( t( RSS_IV ) %*% RSS_IV )  ) - (n_days_ivl_10 /2)

cat("loglik_IV_100_bis ")

loglik_IV_100_bis

IVmlin_100  <- IVmlin

INC <- c(  L0_10000-A0_10, 0, A0_10,  V_10 );

names(INC)= namevar; #assign names to parameters

odestack=NULL
```

IVmlin = NULL

odestack=try(ode(func = mvequations, y = INC, times = t_al, parms=allparsode, method ="daspk")); #runs the ODE equations

odestack=try(lsoda(INC, t_al,mvequations_di,parms=allparsode, atol=atolv,rtol=rtolv)); #runs the ODE equations

if (length(odestack)==1) {cat('!!unresolvable integrator error - triggering early return from optimizer!!'); return(eturmerror) } #catching errors that might happen during fitting

IVmlin =odestack[match(t_ivl_10 ,odestack[,1]),Vpos+1]; #extract values for virus load at time points corresponding to experimental measurements

if (sum(is.na(IVmlin))>0) {cat('!!ODE 10000fail VL NA return!!'); return(eturmerror) } #catching errors that might happen during fitting

if (sum(is.infinite(10^IVmlin))>0) {cat('!!ODE 10000fail VL INF correct!!'); return(eturmerror) } # IVmlin[which(IVmlin>300)] <-300 } #catching errors that might happen during fitting

Smlin=odestack[match(t_al,odestack[,1]),Spos+1]; #extract values for virus load at time points corresponding to experimental measurements

Imlin=odestack[match(t_al ,odestack[,1]),Ipos+1]; #extract values for virus load at time points corresponding to experimental measurements

Amlin_10 =odestack[match(t_al ,odestack[,1]),Apos+1]; #extract values for virus load at time points corresponding to experimental measurements

Lmscal_10 = Smlin + Imlin + Amlin_10

IVmlong_10 =odestack[match(t_al ,odestack[,1]),Vpos+1]; #extract values for virus load at time points corresponding to experimental measurements

correct lod

if (IVmlin[1] <= 0.3) {IVmlin[1] <- 0.3 }

if (IVmlin[2] <= 0.3) {IVmlin[2] <- 0.3 }

if (IVmlin[8] <= 0.3) {IVmlin[8] <- 0.3 }

RSS_IV   <-   log(10^IVmlin) - log(10^m_ivl_10)

loglik_IV_10_bis <- -(n_days_ivl_10 /2)* log((2*pi/n_days_ivl_10) * (t(RSS_IV) %*% RSS_IV)) - (n_days_ivl_10 /2)

```
cat("n_days_ivl_10 ")

n_days_ivl_10


cat("loglik_IV_10_bis ")

loglik_IV_10_bis


IVmlin_10  <- IVmlin




INC <- c(  L0_10000-A0_1, 0, A0_1,  V_1 );


names(INC)= namevar; #assign names to parameters

odestack=NULL

IVmlin = NULL



#  odestack=try(ode( func = mvequations,  y = INC, times = t_al, parms=allparsode, method ="daspk")); #runs the ODE equations

odestack=try(lsoda( INC, t_al,mvequations_di,parms=allparsode, atol=atolv,rtol=rtolv)); #runs the ODE equations

if (length(odestack)==1) {cat('!!unresolvable integrator error - triggering early return from optimizer!!'); return(eturmerror) } #catching errors that might happen during fitting

IVmlin =odestack[match( t_ivl_1 ,odestack[,1]),Vpos+1]; #extract values for virus load at time points corresponding to experimental measurements


cat("sim V1 ")

IVmlin



if (sum(is.na(IVmlin))>0) {cat('!!ODE 10000fail VL NA return!!'); return(eturmerror) } #catching errors that might happen during fitting
```

```
if (sum( is.infinite(10^IVmlin))>0) {cat('!!ODE 10000fail VL INF correct!!');   IVmlin[ which( IVmlin>300 ) ] <-300 } #catching errors that might happen during fitting


Smlin=odestack[match(t_al,odestack[,1]),Spos+1]; #extract values for virus load at time points corresponding to experimental measurements

Imlin=odestack[match( t_al ,odestack[,1]),Ipos+1]; #extract values for virus load at time points corresponding to experimental measurements

Amlin_1 =odestack[match(t_al ,odestack[,1]),Apos+1]; #extract values for virus load at time points corresponding to experimental measurements

Lmscal_1 = Smlin + Imlin + Amlin_1


IVmlong_1 =odestack[match( t_al ,odestack[,1]),Vpos+1]; #extract values for virus load at time points corresponding to experimental measurements


# correct lod

if (IVmlin[1] <= 0.3) {IVmlin[1]  <- 0.3 }

if (IVmlin[2] <= 0.3) {IVmlin[2]  <- 0.3 }

if (IVmlin[3] <= 0.3) {IVmlin[3]  <- 0.3 }


RSS_IV     <-    log(  10^IVmlin )  - log(  10^m_ivl_1 )

loglik_IV_1_bis  <-  -(n_days_ivl_1 /2)*  log(  (2*pi/n_days_ivl_1) * ( t( RSS_IV ) %*% RSS_IV )  ) - (n_days_ivl_1 /2)


cat("n_days_ivl_1 ")

n_days_ivl_1


cat("loglik_IV_1_bis ")

loglik_IV_1_bis


IVmlin_1  <- IVmlin


odestack=NULL

IVmlin = NULL
```

```
loglik_bis <-  loglik_IV_10000_bis   +  loglik_IV_1000_bis   +loglik_IV_100_bis   + loglik_IV_10_bis   +
loglik_IV_1_bis



cat("loglik_bis  ")

loglik_bis



cat("loglik_ref  ")

MSLL_resmatrix["LOGLIK"]

#####################



#####################################################





#MLE <- data.table(param = parnames,

#                estimates = MLE_par,

#               sd = MLE_SE,

#              aicc =  MLE_par*0+ datasim$AICC[1],

#             loglik = MLE_par*0-MLE_estimates$value )


#              TABMLE  <-  kable(MLE)


#             kable(data.table(MSLL_resmatrix))




datafit  <- data.frame(  TCID = c( t_al*0+10^4,   t_al*0+10^3,t_al*0+10^2, t_al*0+10^1, t_al*0+10^0)
,
```

```
            X50. =  c( IVmlong_10000,IVmlong_1000, IVmlong_100, IVmlong_10, IVmlong_1  ) ,
            #mes =  c( m_ivl_10000, m_ivl_1000, m_ivl_100, m_ivl_10, m_ivl_1  ) ,
            tlym = c(Lmscal_10000, Lmscal_1000,Lmscal_100,Lmscal_10,Lmscal_1 ),
            tcell = c(Amlin_10000, Amlin_1000, Amlin_100, Amlin_10, Amlin_1    ),
            t =   c(  t_al, t_al, t_al, t_al,   t_al   ) )


######################################################
##########################################


##################################
 ggplot() +
 #geom_ribbon(aes(ymin = X5., ymax = X95.), fill = "orange", alpha = 0.35) +
 geom_line(data = data94, aes(x = t, y = mes, color=as.factor(TCID) ),  size =0.25) +
 geom_line(data = datafit, aes(x = t, y = X50., color=as.factor(TCID)) , size =1.5, alpha=0.75) +
 geom_point(data = data94,  aes(x = t, y = mes, color=as.factor(TCID)),     size=1.5) +
 #geom_line(mapping = aes( y = si,m_ivl),linetype = "dashed",size=2, color = "blue") +
 labs(x = "Days post infection", y =bquote(  "Log" ~  TCID[50] ~  "/" ~  10^6 ~  "PBMC"))+
 ggtitle("Infectious virus ")  +
 #annotate(geom="text", x=25, y=4, label=mcqii, color="black")+
 labs(color = "Inoculum TCID=")+
 scale_fill_manual(values=cbbPalette)+
 scale_x_continuous(limits=c(0, 30), breaks=c(0,  3,  5,  7,  9, 11, 14, 18, 25, 70) )+
 annotate(geom="text", x=5, y=4,size=4,
      label= paste("MLE= ",round(as.numeric(MSLL_resmatrix["LOGLIK"] ),2), sep=""),
      color="black")+
 annotate(geom="text", x=20, y=4,size=4,
      label= paste("AICc= ",round(as.numeric(MSLL_resmatrix["AICC"] ),2), sep=""),
      color="black")+
 theme(legend.position = "none")
```

```
#################################################
load("~/Anet_MEV_NUS/mv_rcode/mle_MShpc_1994FX_di_res_46u_00_eeflo06688xxLL25.RData")

####################################

A0_10000 <-   MSLL_resmatrix["A_10000"];
A0_1000 <-   MSLL_resmatrix["A_10000"];
A0_100 <-   MSLL_resmatrix["A_10000"];
A0_10 <-   MSLL_resmatrix["A_10000"];
A0_1 <-   MSLL_resmatrix["A_10000"];



V_10000  <- MSLL_resmatrix["V0_10000"];
V_1000  <-   MSLL_resmatrix["V0_1000"];
V_100  <-   MSLL_resmatrix["V0_100"];
V_10  <-   MSLL_resmatrix["V0_10"];
V_1  <-   MSLL_resmatrix["V0_1"];

L0_10000 <-   3906# MSLL_resmatrix["L_10000"];

#Define parameter value

allparsode <-   c( 0.028, # MSLL_resmatrix["qs"] ,
                2.6 , #MSLL_resmatrix["td"],
                MSLL_resmatrix["b"] ,
                0.5 ,
                MSLL_resmatrix["k"],
                1.11, # MSLL_resmatrix["q"],
                MSLL_resmatrix["s"],
                0.025,
```

```
        0.016, #  MSLL_resmatrix["r"],
        MSLL_resmatrix["p"]  ,
        3 )

names(allparsode)=defparnames; #assign names to parameters

cat("best para ")
allparsode

# RUN ODE
#print(sprintf(' start run ode ' ))
INC <- c(  L0_10000-A0_10000, 0, A0_10000,  V_10000 );
names(INC)= namevar; #assign names to parameters

cat("best INC 10000")
INC

odestack=NULL
IVmlin = NULL
#  odestack=try(ode( func = mvequations,  y = INC, times = t_al, parms=allparsode, method ="daspk")); #runs the ODE equations

odestack=try(lsoda( INC, t_al,mvequations_di,parms=allparsode, atol=atolv,rtol=rtolv)); #runs the ODE equations
if (length(odestack)==1) {cat('!!unresolvable integrator error - triggering early return from optimizer!!'); return(eturmerror) } #catching errors that might happen during fitting
IVmlin =odestack[match( t_ivl_10 ,odestack[,1]),Vpos+1]; #extract values for virus load at time points corresponding to experimental measurements

cat("sim V 10000 ")
```

IVmlin

#plot(t_ivl_10,   m_ivl_10000)

if (sum(is.na(IVmlin))>0) {cat('!!ODE 10000fail VL NA return!!'); return(eturmerror) } #catching errors that might happen during fitting

if (sum(is.infinite(10^IVmlin))>0) {cat('!!ODE 10000fail VL INF correct!!'); return(eturmerror) } # IVmlin[which(IVmlin>300)] <-300 } #catching errors that might happen during fitting

Smlin=odestack[match(t_al,odestack[,1]),Spos+1]; #extract values for virus load at time points corresponding to experimental measurements

Imlin=odestack[match(t_al ,odestack[,1]),Ipos+1]; #extract values for virus load at time points corresponding to experimental measurements

Amlin_10000=odestack[match(t_al ,odestack[,1]),Apos+1]; #extract values for virus load at time points corresponding to experimental measurements

Lmscal_10000 = Smlin + Imlin + Amlin_10000

#if (Lmscal_10000[length(Lmscal_10000)]< 2000) {cat('!!ODE 10000faIL LOW TLYM'); return(eturmerror) }

IVmlong_10000 =odestack[match(t_al ,odestack[,1]),Vpos+1]; #extract values for virus load at time points corresponding to experimental measurements

correct lod

if (IVmlin[1] <= 0.3) {IVmlin[1] <- 0.3 }

if (IVmlin[7] <= 0.3) {IVmlin[7] <- 0.3 }

if (IVmlin[8] <= 0.3) {IVmlin[8] <- 0.3 }

RSS_IV   <-   log(10^IVmlin) - log(10^m_ivl_10000)

loglik_IV_10000_bis <- -(n_days_ivl_10 /2)* log((2*pi/n_days_ivl_10) * (t(RSS_IV) %*% RSS_IV)) -(n_days_ivl_10 /2)

cat("loglik_IV_10000_bis ")

loglik_IV_10000_bis

IVmlin_10000 <- IVmlin

INC <- c(L0_10000-A0_1000, 0, A0_1000, V_1000);

names(INC)= namevar; #assign names to parameters

odestack=NULL

IVmlin = NULL

odestack=try(ode(func = mvequations, y = INC, times = t_al, parms=allparsode, method ="daspk")); #runs the ODE equations

odestack=try(lsoda(INC, t_al,mvequations_di,parms=allparsode, atol=atolv,rtol=rtolv)); #runs the ODE equations

if (length(odestack)==1) {cat('!!unresolvable integrator error - triggering early return from optimizer!!'); return(eturmerror) } #catching errors that might happen during fitting

IVmlin =odestack[match(t_ivl_10 ,odestack[,1]),Vpos+1]; #extract values for virus load at time points corresponding to experimental measurements

if (sum(is.na(IVmlin))>0) {cat('!!ODE 10000fail VL NA return!!'); return(eturmerror) } #catching errors that might happen during fitting

if (sum(is.infinite(10^IVmlin))>0) {cat('!!ODE 10000fail VL INF correct!!'); return(eturmerror) } # IVmlin[which(IVmlin>300)] <-300 } #catching errors that might happen during fitting

Smlin=odestack[match(t_al,odestack[,1]),Spos+1]; #extract values for virus load at time points corresponding to experimental measurements

Imlin=odestack[match(t_al ,odestack[,1]),Ipos+1]; #extract values for virus load at time points corresponding to experimental measurements

Amlin_1000 =odestack[match(t_al ,odestack[,1]),Apos+1]; #extract values for virus load at time points corresponding to experimental measurements

Lmscal_1000 = Smlin + Imlin + Amlin_1000

```
IVmlong_1000 =odestack[match( t_al ,odestack[,1]),Vpos+1]; #extract values for virus load at time points corresponding to experimental measurements


# correct lod

if (IVmlin[1] <= 0.3) {IVmlin[1]  <- 0.3 }

if (IVmlin[2] <= 0.3) {IVmlin[2]  <- 0.3 }

if (IVmlin[8] <= 0.3) {IVmlin[8]  <- 0.3 }


RSS_IV     <-    log(  10^IVmlin )  - log(  10^m_ivl_1000 )

loglik_IV_1000_bis  <-  -(n_days_ivl_10 /2)*  log(  (2*pi/n_days_ivl_10) * ( t( RSS_IV ) %*% RSS_IV )  ) -(n_days_ivl_10 /2)


cat("loglik_IV_1000_bis ")

loglik_IV_1000_bis


IVmlin_1000  <- IVmlin



INC <- c(  L0_10000-A0_100, 0, A0_100,  V_100 );


names(INC)= namevar; #assign names to parameters

odestack=NULL

IVmlin = NULL



#  odestack=try(ode( func = mvequations,  y = INC, times = t_al, parms=allparsode, method ="daspk")); #runs the ODE equations

odestack=try(lsoda( INC, t_al,mvequations_di,parms=allparsode, atol=atolv,rtol=rtolv)); #runs the ODE equations

if (length(odestack)==1) {cat('!!unresolvable integrator error - triggering early return from optimizer!!'); return(eturmerror) } #catching errors that might happen during fitting

IVmlin =odestack[match( t_ivl_10 ,odestack[,1]),Vpos+1]; #extract values for virus load at time points corresponding to experimental measurements
```

```
if (sum(is.na(lVmlin))>0) {cat('!!ODE 10000fail VL NA return!!'); return(eturmerror) } #catching errors that might happen during fitting

if (sum( is.infinite(10^lVmlin))>0) {cat('!!ODE 10000fail VL INF correct!!');  return(eturmerror) } # lVmlin[ which( lVmlin>300  ) ] <-300 } #catching errors that might happen during fitting


Smlin=odestack[match(t_al,odestack[,1]),Spos+1]; #extract values for virus load at time points corresponding to experimental measurements

Imlin=odestack[match( t_al ,odestack[,1]),Ipos+1]; #extract values for virus load at time points corresponding to experimental measurements

Amlin_100=odestack[match(t_al ,odestack[,1]),Apos+1]; #extract values for virus load at time points corresponding to experimental measurements

Lmscal_100 = Smlin + Imlin + Amlin_100


lVmlong_100 =odestack[match( t_al ,odestack[,1]),Vpos+1]; #extract values for virus load at time points corresponding to experimental measurements


# correct lod

if (lVmlin[1] <= 0.3) {lVmlin[1]  <- 0.3 }

if (lVmlin[2] <= 0.3) {lVmlin[2]  <- 0.3 }

if (lVmlin[8] <= 0.3) {lVmlin[8]  <- 0.3 }


RSS_IV     <-    log(  10^lVmlin )  - log(  10^m_ivl_100 )

loglik_IV_100_bis  <-  -(n_days_ivl_10 /2)*  log(  (2*pi/n_days_ivl_10) * ( t( RSS_IV ) %*% RSS_IV )  ) - (n_days_ivl_10 /2)


cat("loglik_IV_100_bis ")

loglik_IV_100_bis


lVmlin_100  <- lVmlin


INC <- c(  L0_10000-A0_10, 0, A0_10,  V_10 );
```

names(INC)= namevar; #assign names to parameters

odestack=NULL

IVmlin = NULL

odestack=try(ode(func = mvequations, y = INC, times = t_al, parms=allparsode, method ="daspk")); #runs the ODE equations

odestack=try(lsoda(INC, t_al,mvequations_di,parms=allparsode, atol=atolv,rtol=rtolv)); #runs the ODE equations

if (length(odestack)==1) {cat('!!unresolvable integrator error - triggering early return from optimizer!!'); return(eturmerror) } #catching errors that might happen during fitting

IVmlin =odestack[match(t_ivl_10 ,odestack[,1]),Vpos+1]; #extract values for virus load at time points corresponding to experimental measurements

if (sum(is.na(IVmlin))>0) {cat('!!ODE 10000fail VL NA return!!'); return(eturmerror) } #catching errors that might happen during fitting

if (sum(is.infinite(10^IVmlin))>0) {cat('!!ODE 10000fail VL INF correct!!'); return(eturmerror) } # IVmlin[which(IVmlin>300)] <-300 } #catching errors that might happen during fitting

Smlin=odestack[match(t_al,odestack[,1]),Spos+1]; #extract values for virus load at time points corresponding to experimental measurements

Imlin=odestack[match(t_al ,odestack[,1]),Ipos+1]; #extract values for virus load at time points corresponding to experimental measurements

Amlin_10 =odestack[match(t_al ,odestack[,1]),Apos+1]; #extract values for virus load at time points corresponding to experimental measurements

Lmscal_10 = Smlin + Imlin + Amlin_10

IVmlong_10 =odestack[match(t_al ,odestack[,1]),Vpos+1]; #extract values for virus load at time points corresponding to experimental measurements

correct lod

if (IVmlin[1] <= 0.3) {IVmlin[1] <- 0.3 }

if (IVmlin[2] <= 0.3) {IVmlin[2] <- 0.3 }

if (IVmlin[8] <= 0.3) {IVmlin[8] <- 0.3 }

```
RSS_IV     <-    log(  10^IVmlin )  - log(  10^m_ivl_10 )

loglik_IV_10_bis  <-  -(n_days_ivl_10 /2)*  log(  (2*pi/n_days_ivl_10) * ( t( RSS_IV ) %*% RSS_IV )  ) - (n_days_ivl_10 /2)


cat("n_days_ivl_10 ")

n_days_ivl_10


cat("loglik_IV_10_bis ")

loglik_IV_10_bis


IVmlin_10  <- IVmlin




INC <- c(  L0_10000-A0_1, 0, A0_1,  V_1 );


names(INC)= namevar; #assign names to parameters

odestack=NULL

IVmlin = NULL



#  odestack=try(ode( func = mvequations,  y = INC, times = t_al, parms=allparsode, method ="daspk")); #runs the ODE equations

odestack=try(lsoda( INC, t_al,mvequations_di,parms=allparsode, atol=atolv,rtol=rtolv)); #runs the ODE equations

if (length(odestack)==1) {cat('!!unresolvable integrator error - triggering early return from optimizer!!'); return(eturmerror) } #catching errors that might happen during fitting

IVmlin =odestack[match( t_ivl_1 ,odestack[,1]),Vpos+1]; #extract values for virus load at time points corresponding to experimental measurements


cat("sim V1 ")

IVmlin
```

```
if (sum(is.na(IVmlin))>0) {cat('!!ODE 10000fail VL NA return!!'); return(eturmerror) } #catching errors that might happen during fitting

if (sum( is.infinite(10^IVmlin))>0) {cat('!!ODE 10000fail VL INF correct!!');   IVmlin[ which( IVmlin>300 ) ] <-300 } #catching errors that might happen during fitting


Smlin=odestack[match(t_al,odestack[,1]),Spos+1]; #extract values for virus load at time points corresponding to experimental measurements

Imlin=odestack[match( t_al ,odestack[,1]),Ipos+1]; #extract values for virus load at time points corresponding to experimental measurements

Amlin_1 =odestack[match(t_al ,odestack[,1]),Apos+1]; #extract values for virus load at time points corresponding to experimental measurements

Lmscal_1 = Smlin + Imlin + Amlin_1


IVmlong_1 =odestack[match( t_al ,odestack[,1]),Vpos+1]; #extract values for virus load at time points corresponding to experimental measurements


# correct lod

if (IVmlin[1] <= 0.3) {IVmlin[1]  <- 0.3 }

if (IVmlin[2] <= 0.3) {IVmlin[2]  <- 0.3 }

if (IVmlin[3] <= 0.3) {IVmlin[3]  <- 0.3 }


RSS_IV     <-    log(  10^IVmlin )  - log(  10^m_ivl_1 )

loglik_IV_1_bis  <-  -(n_days_ivl_1 /2)*  log(  (2*pi/n_days_ivl_1) * ( t( RSS_IV ) %*% RSS_IV )  ) - (n_days_ivl_1 /2)


cat("n_days_ivl_1 ")

n_days_ivl_1


cat("loglik_IV_1_bis ")

loglik_IV_1_bis


IVmlin_1  <- IVmlin
```

```
odestack=NULL

IVmlin = NULL

loglik_bis <-  loglik_IV_10000_bis   +  loglik_IV_1000_bis   +loglik_IV_100_bis   + loglik_IV_10_bis   +
loglik_IV_1_bis

cat("loglik_bis  ")

loglik_bis

cat("loglik_ref  ")

MSLL_resmatrix["LOGLIK"]

#####################

#####################################################

#MLE <- data.table(param = parnames,

#               estimates = MLE_par,

#              sd = MLE_SE,

#             aicc =  MLE_par*0+ datasim$AICC[1],

#            loglik = MLE_par*0-MLE_estimates$value )

#             TABMLE  <-  kable(MLE)

#            kable(data.table(MSLL_resmatrix))
```

```
datafit  <- data.frame(  TCID = c( t_al*0+10^4,   t_al*0+10^3,t_al*0+10^2, t_al*0+10^1, t_al*0+10^0)
,
                    X50. =  c( IVmlong_10000,IVmlong_1000, IVmlong_100, IVmlong_10, IVmlong_1  ) ,
                    #mes =  c( m_ivl_10000, m_ivl_1000, m_ivl_100, m_ivl_10, m_ivl_1  ) ,
                    tlym = c(Lmscal_10000, Lmscal_1000,Lmscal_100,Lmscal_10,Lmscal_1 ),
                    tcell = c(Amlin_10000, Amlin_1000, Amlin_100, Amlin_10, Amlin_1    ),
                    t =   c(  t_al, t_al, t_al, t_al,   t_al   ) )


max(Amlin_10000)

max(  datafit$tcell )
####################################

library(forcats)

data94$categ <- fct_rev( as.factor( data94$TCID))

datafit$categ <- fct_rev( as.factor( datafit$TCID))


FIG_DI <-      ggplot(  data = datafit, aes(x = t, y = X50.,  color=categ, fill=categ,   group=categ ) ) +
  geom_line(  size =1.25) +
  geom_point(data = data94,  aes(x = t, y = mes,   color=categ, fill=categ, shape=categ),     size=2) +
  scale_shape_manual( values=c( 18, 8,17,19,  15)) +
  scale_fill_manual(values=cbbPalette) +
  scale_color_manual(values=cbbPalette) +
  labs( shape = bquote(  TCID[50] ~ "="), fill = bquote(  TCID[50] ~ "="), color = bquote(  TCID[50] ~
"=") ) +
  #geom_ribbon(aes(ymin = X5., ymax = X95.), fill = "orange", alpha = 0.35) +
```

```
 #geom_point(data = data94,  aes(x = t, y = mes,   color=as.factor(TCID)),   size=2) +
 #geom_line(mapping = aes( y = si,m_ivl),linetype = "dashed",size=2, color = "blue") +
 labs(x = "Days post MV infection", y =bquote(  "Log" ~  TCID[50] ~  "/" ~  10^6 ~  "PBMC"))+
 ggtitle("Infectious viremia ")  +
 #annotate(geom="text", x=25, y=4, label=mcqii, color="black")+
 theme(legend.position="bottom")   +
 theme(legend.text = element_text(colour="black",  face="bold")) +
 geom_hline(yintercept = 0.3, linetype = "dotdash", color = "darkgray", size=1) +
 scale_x_continuous( limits=c(0, 30),  breaks=c(0,  3,  5,  7,  9,  13,  17, 20,25,30 ))+
 scale_y_continuous(limits = c(0,4),breaks=c(0.3,1,2,  3,3.4,  4 ))+
 annotate(geom="text", x=8, y=4,size=2.5,
        label= paste("B1: MLE=",round(as.numeric(MSLL_resmatrix["LOGLIK"] ),2), sep=""),
        color="black")+
 annotate(geom="text", x=23, y=4,size=2.5,
        label= paste("AICc=",round(as.numeric(MSLL_resmatrix["AICC"] ),2), sep=""),
        color="black")  +
 theme(legend.position = "none")



fig_DI
MSLL_resmatrix



################################################



load("~/Anet_MEV_NUS/mv_rcode/mle_MShpc_1994FX_IC_res_46u_050_eeflo06688xxLLv2.RData"
)
cat("best fit ")
MSLL_resmatrix
```

```
####################################

A0_10000 <-   MSLL_resmatrix["A_10000"]/1
A0_1000 <-   MSLL_resmatrix["A_10000"]/1
A0_100 <-   MSLL_resmatrix["A_10000"]/1
A0_10 <-   MSLL_resmatrix["A_10000"]/1
A0_1 <-   MSLL_resmatrix["A_10000"]/1


V_10000  <- MSLL_resmatrix["V0_10000"];
V_1000  <-   MSLL_resmatrix["V0_1000"];
V_100  <-   MSLL_resmatrix["V0_100"];
V_10  <-   MSLL_resmatrix["V0_10"];
V_1  <-   MSLL_resmatrix["V0_1"];

L0_10000 <-   3906# MSLL_resmatrix["L_10000"];

#Define parameter value

allparsode <-   c( 0.028, # MSLL_resmatrix["qs"] ,
            2.6 , #MSLL_resmatrix["td"],
            MSLL_resmatrix["b"] ,
            0.5 ,
            MSLL_resmatrix["k"],
            1.11, # MSLL_resmatrix["q"],
            MSLL_resmatrix["s"],
            0.025,
            0.016, #  MSLL_resmatrix["r"],
            MSLL_resmatrix["p"]  ,
            3 )
```

```
names(allparsode)=defparnames; #assign names to parameters

cat("best para ")
allparsode



# RUN ODE
#print(sprintf(' start run ode ' ))
INC <- c(  L0_10000-A0_10000, 0, A0_10000,  V_10000 );
names(INC)= namevar; #assign names to parameters

cat("best INC 10000")
INC



odestack=NULL
IVmlin = NULL
#  odestack=try(ode( func = mvequations,  y = INC, times = t_al, parms=allparsode, method
="daspk")); #runs the ODE equations

odestack=try(lsoda( INC, t_al,mvequations,parms=allparsode, atol=atolv,rtol=rtolv)); #runs the ODE
equations
if (length(odestack)==1) {cat('!!unresolvable integrator error - triggering early return from
optimizer!!'); return(eturmerror) } #catching errors that might happen during fitting
IVmlin =odestack[match( t_ivl_10 ,odestack[,1]),Vpos+1]; #extract values for virus load at time points
corresponding to experimental measurements

cat("sim V 10000 ")
IVmlin

#plot( t_ivl_10,   m_ivl_10000)
```

```
if (sum(is.na(IVmlin))>0) {cat('!!ODE 10000fail VL NA return!!'); return(eturmerror) } #catching errors that might happen during fitting

if (sum( is.infinite(10^IVmlin))>0) {cat('!!ODE 10000fail VL INF correct!!');  return(eturmerror) } # IVmlin[ which( IVmlin>300  ) ] <-300 } #catching errors that might happen during fitting



Smlin=odestack[match(t_al,odestack[,1]),Spos+1]; #extract values for virus load at time points corresponding to experimental measurements

Imlin=odestack[match( t_al ,odestack[,1]),Ipos+1]; #extract values for virus load at time points corresponding to experimental measurements

Amlin_10000=odestack[match(t_al ,odestack[,1]),Apos+1]; #extract values for virus load at time points corresponding to experimental measurements

Lmscal_10000 = Smlin + Imlin + Amlin_10000

#if (Lmscal_10000[length(Lmscal_10000)]< 2000) {cat('!!ODE 10000faIL LOW TLYM'); return(eturmerror) }


IVmlong_10000 =odestack[match( t_al ,odestack[,1]),Vpos+1]; #extract values for virus load at time points corresponding to experimental measurements


# correct lod

if (IVmlin[1] <= 0.3) {IVmlin[1]  <- 0.3 }

if (IVmlin[7] <= 0.3) {IVmlin[7]  <- 0.3 }

if (IVmlin[8] <= 0.3) {IVmlin[8]  <- 0.3 }


RSS_IV     <-    log( 10^IVmlin )  - log( 10^m_ivl_10000 )


loglik_IV_10000_bis  <-  -(n_days_ivl_10 /2)*  log(  (2*pi/n_days_ivl_10) * ( t( RSS_IV ) %*% RSS_IV ) ) -(n_days_ivl_10 /2)



IVmlin_10000  <- IVmlin
```

```
INC <- c(  L0_10000-A0_1000, 0, A0_1000,  V_1000 );

names(INC)= namevar; #assign names to parameters

odestack=NULL

IVmlin = NULL



#  odestack=try(ode( func = mvequations,  y = INC, times = t_al, parms=allparsode, method ="daspk")); #runs the ODE equations

odestack=try(lsoda( INC, t_al,mvequations,parms=allparsode, atol=atolv,rtol=rtolv)); #runs the ODE equations

if (length(odestack)==1) {cat('!!unresolvable integrator error - triggering early return from optimizer!!'); return(eturmerror) } #catching errors that might happen during fitting

IVmlin =odestack[match( t_ivl_10 ,odestack[,1]),Vpos+1]; #extract values for virus load at time points corresponding to experimental measurements

if (sum(is.na(IVmlin))>0) {cat('!!ODE 10000fail VL NA return!!'); return(eturmerror) } #catching errors that might happen during fitting

if (sum( is.infinite(10^IVmlin))>0) {cat('!!ODE 10000fail VL INF correct!!');  return(eturmerror) } # IVmlin[ which( IVmlin>300  ) ] <-300 } #catching errors that might happen during fitting


Smlin=odestack[match(t_al,odestack[,1]),Spos+1]; #extract values for virus load at time points corresponding to experimental measurements

Imlin=odestack[match( t_al ,odestack[,1]),Ipos+1]; #extract values for virus load at time points corresponding to experimental measurements

Amlin_1000 =odestack[match(t_al ,odestack[,1]),Apos+1]; #extract values for virus load at time points corresponding to experimental measurements

Lmscal_1000 = Smlin + Imlin + Amlin_1000

IVmlong_1000 =odestack[match( t_al ,odestack[,1]),Vpos+1]; #extract values for virus load at time points corresponding to experimental measurements


# correct lod

if (IVmlin[1] <= 0.3) {IVmlin[1]  <- 0.3 }

if (IVmlin[2] <= 0.3) {IVmlin[2]  <- 0.3 }
```

```
if (IVmlin[8] <= 0.3) {IVmlin[8]  <- 0.3 }


RSS_IV     <-    log(  10^IVmlin )  - log(  10^m_ivl_1000 )

loglik_IV_1000_bis  <-  -(n_days_ivl_10 /2)*  log(  (2*pi/n_days_ivl_10) * ( t( RSS_IV ) %*% RSS_IV )  ) -(n_days_ivl_10 /2)


IVmlin_1000  <- IVmlin


INC <- c(  L0_10000-A0_100, 0, A0_100,  V_100 );


names(INC)= namevar; #assign names to parameters

odestack=NULL

IVmlin = NULL


#  odestack=try(ode( func = mvequations,  y = INC, times = t_al, parms=allparsode, method ="daspk")); #runs the ODE equations

odestack=try(lsoda( INC, t_al,mvequations,parms=allparsode, atol=atolv,rtol=rtolv)); #runs the ODE equations

if (length(odestack)==1) {cat('!!unresolvable integrator error - triggering early return from optimizer!!'); return(eturmerror) } #catching errors that might happen during fitting

IVmlin =odestack[match( t_ivl_10 ,odestack[,1]),Vpos+1]; #extract values for virus load at time points corresponding to experimental measurements

if (sum(is.na(IVmlin))>0) {cat('!!ODE 10000fail VL NA return!!'); return(eturmerror) } #catching errors that might happen during fitting

if (sum( is.infinite(10^IVmlin))>0) {cat('!!ODE 10000fail VL INF correct!!');  return(eturmerror) } # IVmlin[ which( IVmlin>300  ) ] <-300 } #catching errors that might happen during fitting


Smlin=odestack[match(t_al,odestack[,1]),Spos+1]; #extract values for virus load at time points corresponding to experimental measurements
```

Imlin=odestack[match(t_al ,odestack[,1]),Ipos+1]; #extract values for virus load at time points corresponding to experimental measurements

Amlin_100=odestack[match(t_al ,odestack[,1]),Apos+1]; #extract values for virus load at time points corresponding to experimental measurements

Lmscal_100 = Smlin + Imlin + Amlin_100

IVmlong_100 =odestack[match(t_al ,odestack[,1]),Vpos+1]; #extract values for virus load at time points corresponding to experimental measurements

correct lod

if (IVmlin[1] <= 0.3) {IVmlin[1] <- 0.3 }

if (IVmlin[2] <= 0.3) {IVmlin[2] <- 0.3 }

if (IVmlin[8] <= 0.3) {IVmlin[8] <- 0.3 }

RSS_IV   <-   log(10^IVmlin) - log(10^m_ivl_100)

loglik_IV_100_bis <- -(n_days_ivl_10 /2)* log((2*pi/n_days_ivl_10) * (t(RSS_IV) %*% RSS_IV)) - (n_days_ivl_10 /2)

IVmlin_100 <- IVmlin

INC <- c(L0_10000-A0_10, 0, A0_10, V_10);

names(INC)= namevar; #assign names to parameters

odestack=NULL

IVmlin = NULL

odestack=try(ode(func = mvequations, y = INC, times = t_al, parms=allparsode, method ="daspk")); #runs the ODE equations

odestack=try(lsoda(INC, t_al,mvequations,parms=allparsode, atol=atolv,rtol=rtolv)); #runs the ODE equations

```
if (length(odestack)==1) {cat('!!unresolvable integrator error - triggering early return from optimizer!!'); return(eturmerror) } #catching errors that might happen during fitting

IVmlin =odestack[match( t_ivl_10 ,odestack[,1]),Vpos+1]; #extract values for virus load at time points corresponding to experimental measurements

if (sum(is.na(IVmlin))>0) {cat('!!ODE 10000fail VL NA return!!'); return(eturmerror) } #catching errors that might happen during fitting

if (sum( is.infinite(10^IVmlin))>0) {cat('!!ODE 10000fail VL INF correct!!');  return(eturmerror) } # IVmlin[ which( IVmlin>300  ) ] <-300 } #catching errors that might happen during fitting


Smlin=odestack[match(t_al,odestack[,1]),Spos+1]; #extract values for virus load at time points corresponding to experimental measurements

Imlin=odestack[match( t_al ,odestack[,1]),Ipos+1]; #extract values for virus load at time points corresponding to experimental measurements

Amlin_10 =odestack[match(t_al ,odestack[,1]),Apos+1]; #extract values for virus load at time points corresponding to experimental measurements

Lmscal_10 = Smlin + Imlin + Amlin_10


IVmlong_10 =odestack[match( t_al ,odestack[,1]),Vpos+1]; #extract values for virus load at time points corresponding to experimental measurements


# correct lod

if (IVmlin[1] <= 0.3) {IVmlin[1]  <- 0.3 }

if (IVmlin[2] <= 0.3) {IVmlin[2]  <- 0.3 }

if (IVmlin[8] <= 0.3) {IVmlin[8]  <- 0.3 }


RSS_IV     <-    log(  10^IVmlin )  - log(  10^m_ivl_10 )

loglik_IV_10_bis  <-  -(n_days_ivl_10 /2)*  log(  (2*pi/n_days_ivl_10) * ( t( RSS_IV ) %*% RSS_IV )  ) - (n_days_ivl_10 /2)


IVmlin_10  <- IVmlin
```

```
INC <- c(  L0_10000-A0_1, 0, A0_1,  V_1 );

names(INC)= namevar; #assign names to parameters

odestack=NULL

IVmlin = NULL

#  odestack=try(ode( func = mvequations,  y = INC, times = t_al, parms=allparsode, method ="daspk")); #runs the ODE equations

odestack=try(lsoda( INC, t_al,mvequations,parms=allparsode, atol=atolv,rtol=rtolv)); #runs the ODE equations

if (length(odestack)==1) {cat('!!unresolvable integrator error - triggering early return from optimizer!!'); return(eturmerror) } #catching errors that might happen during fitting

IVmlin =odestack[match( t_ivl_1 ,odestack[,1]),Vpos+1]; #extract values for virus load at time points corresponding to experimental measurements

cat("sim V1 ")

IVmlin

if (sum(is.na(IVmlin))>0) {cat('!!ODE 10000fail VL NA return!!'); return(eturmerror) } #catching errors that might happen during fitting

if (sum( is.infinite(10^IVmlin))>0) {cat('!!ODE 10000fail VL INF correct!!');   IVmlin[ which( IVmlin>300 ) ] <-300 } #catching errors that might happen during fitting

Smlin=odestack[match(t_al,odestack[,1]),Spos+1]; #extract values for virus load at time points corresponding to experimental measurements

Imlin=odestack[match( t_al ,odestack[,1]),Ipos+1]; #extract values for virus load at time points corresponding to experimental measurements

Amlin_1 =odestack[match(t_al ,odestack[,1]),Apos+1]; #extract values for virus load at time points corresponding to experimental measurements
```

```
Lmscal_1 = Smlin + Imlin + Amlin_1

IVmlong_1 =odestack[match( t_al ,odestack[,1]),Vpos+1]; #extract values for virus load at time points corresponding to experimental measurements

# correct lod
if (IVmlin[1] <= 0.3) {IVmlin[1]  <- 0.3 }
if (IVmlin[2] <= 0.3) {IVmlin[2]  <- 0.3 }
if (IVmlin[3] <= 0.3) {IVmlin[3]  <- 0.3 }

RSS_IV     <-    log(  10^IVmlin )  - log(  10^m_ivl_1 )
loglik_IV_1_bis  <-  -(n_days_ivl_1 /2)*  log(  (2*pi/n_days_ivl_1) * ( t( RSS_IV ) %*% RSS_IV )  ) - (n_days_ivl_1 /2)

cat("n_days_ivl_1 ")
n_days_ivl_1

IVmlin_1  <- IVmlin

odestack=NULL
IVmlin = NULL

loglik_bis <-  loglik_IV_10000_bis   +  loglik_IV_1000_bis   +loglik_IV_100_bis   + loglik_IV_10_bis   + loglik_IV_1_bis

cat("loglik_bis")
loglik_bis
```

```
cat("loglik_ref  ")

MSLL_resmatrix["LOGLIK"]

#####################



#####################################################




#MLE <- data.table(param = parnames,

#               estimates = MLE_par,

#              sd = MLE_SE,

#             aicc =  MLE_par*0+ datasim$AICC[1],

#            loglik = MLE_par*0-MLE_estimates$value )


#             TABMLE  <-  kable(MLE)


#            kable(data.table(MSLL_resmatrix))




datafit  <- data.frame(  TCID = c( t_al*0+10^4,   t_al*0+10^3,t_al*0+10^2, t_al*0+10^1, t_al*0+10^0)
,
                     X50. =  c( IVmlong_10000,IVmlong_1000, IVmlong_100, IVmlong_10, IVmlong_1  ) ,

                     #mes =  c( m_ivl_10000, m_ivl_1000, m_ivl_100, m_ivl_10, m_ivl_1  ) ,

                     tlym = c(Lmscal_10000, Lmscal_1000,Lmscal_100,Lmscal_10,Lmscal_1 ),

                     tcell = c(Amlin_10000, Amlin_1000, Amlin_100, Amlin_10, Amlin_1    ),

                     t =   c(  t_al, t_al, t_al, t_al,   t_al   ) )



####################
```

```
library(forcats)

data94$categ <- fct_rev( as.factor( data94$TCID))

datafit$categ <- fct_rev( as.factor( datafit$TCID))

FIG_IC <-     ggplot(  data = datafit, aes(x = t, y = X50.,  color=categ, fill=categ,   group=categ ) ) +
  geom_line(  size =1.25) +
  geom_point(data = data94,  aes(x = t, y = mes,   color=categ, fill=categ, shape=categ),     size=2) +
  scale_shape_manual( values=c( 18, 8,17,19,  15)) +
  scale_fill_manual(values=cbbPalette) +
  scale_color_manual(values=cbbPalette) +
  labs( shape = bquote(  TCID[50] ~ "="), fill = bquote(  TCID[50] ~ "="), color = bquote(  TCID[50] ~
"=") ) +
  #geom_ribbon(aes(ymin = X5., ymax = X95.), fill = "orange", alpha = 0.35) +
  #geom_point(data = data94,  aes(x = t, y = mes,   color=as.factor(TCID)),   size=2) +
  #geom_line(mapping = aes( y = si,m_ivl),linetype = "dashed",size=2, color = "blue") +
  labs(x = "Days post MV infection", y =bquote(  "Log" ~  TCID[50] ~  "/" ~  10^6 ~  "PBMC"))+
  ggtitle("Infectious viremia ")  +
  #annotate(geom="text", x=25, y=4, label=mcqii, color="black")+
  theme(legend.position="none")   +
  theme(legend.text = element_text(colour="black",  face="bold")) +
  geom_hline(yintercept = 0.3, linetype = "dotdash", color = "darkgray", size=1 ) +
  scale_x_continuous( limits=c(0, 30),  breaks=c(0,  3,  5,  7,  9,  13,  17, 20,25,30 ))+
  scale_y_continuous(limits = c(0,4),breaks=c(0.3,1,2,  3,3.4,  4 ))+
  annotate(geom="text", x=8, y=4,size=2.5,
        label= paste("A1: MLE=",round(as.numeric(MSLL_resmatrix["LOGLIK"] ),2), sep=""),
        color="black")+
```

```
  annotate(geom="text", x=23, y=4,size=2.5,
           label= paste("AICc=",round(as.numeric(MSLL_resmatrix["AICC"] ),2), sep=""),
           color="black")


FIG_IC

######################################################
load("~/Anet_MEV_NUS/mv_rcode/mle_MShpc_1994FX_ICic_res_46u_10_eeflo8xxLL.RData")

######################################


A0_10000 <-   MSLL_resmatrix["A_10000"];
A0_1000 <-   MSLL_resmatrix["A_1000"];
A0_100 <-   MSLL_resmatrix["A_100"];
A0_10 <-   MSLL_resmatrix["A_10"];
A0_1 <-   MSLL_resmatrix["A_1"];



V_10000  <- MSLL_resmatrix["V0_10000"];
V_1000  <-   MSLL_resmatrix["V0_1000"];
V_100  <-   MSLL_resmatrix["V0_100"];
V_10  <-   MSLL_resmatrix["V0_10"];
V_1  <-   MSLL_resmatrix["V0_1"];

L0_10000 <-   3906# MSLL_resmatrix["L_10000"];

#Define parameter value

allparsode <-   c( 0.028, # MSLL_resmatrix["qs"] ,
                2.6 , #MSLL_resmatrix["td"],
```

```
                MSLL_resmatrix["b"] ,
                0.5 ,
                MSLL_resmatrix["k"],
                1.11, # MSLL_resmatrix["q"],
                MSLL_resmatrix["s"],
                0.025,
                0.016, #  MSLL_resmatrix["r"],
                MSLL_resmatrix["p"]  ,
                3 )

names(allparsode)=defparnames; #assign names to parameters


cat("best para ")
allparsode



# RUN ODE
#print(sprintf(' start run ode ' ))
INC <- c(  L0_10000-A0_10000, 0, A0_10000,  V_10000 );
names(INC)= namevar; #assign names to parameters

cat("best INC 10000")
INC



odestack=NULL
IVmlin = NULL
#  odestack=try(ode( func = mvequations,  y = INC, times = t_al, parms=allparsode, method ="daspk")); #runs the ODE equations


odestack=try(lsoda( INC, t_al,mvequations,parms=allparsode, atol=atolv,rtol=rtolv)); #runs the ODE equations
```

```
if (length(odestack)==1) {cat('!!unresolvable integrator error - triggering early return from optimizer!!'); return(eturmerror) } #catching errors that might happen during fitting

IVmlin =odestack[match( t_ivl_10 ,odestack[,1]),Vpos+1]; #extract values for virus load at time points corresponding to experimental measurements


cat("sim V 10000 ")

IVmlin


#plot( t_ivl_10,   m_ivl_10000)


if (sum(is.na(IVmlin))>0) {cat('!!ODE 10000fail VL NA return!!'); return(eturmerror) } #catching errors that might happen during fitting

if (sum( is.infinite(10^IVmlin))>0) {cat('!!ODE 10000fail VL INF correct!!');  return(eturmerror) } # IVmlin[ which( IVmlin>300  ) ] <-300 } #catching errors that might happen during fitting



Smlin=odestack[match(t_al,odestack[,1]),Spos+1]; #extract values for virus load at time points corresponding to experimental measurements

Imlin=odestack[match( t_al ,odestack[,1]),Ipos+1]; #extract values for virus load at time points corresponding to experimental measurements

Amlin_10000=odestack[match(t_al ,odestack[,1]),Apos+1]; #extract values for virus load at time points corresponding to experimental measurements

Lmscal_10000 = Smlin + Imlin + Amlin_10000

#if (Lmscal_10000[length(Lmscal_10000)]< 2000) {cat('!!ODE 10000faIL LOW TLYM'); return(eturmerror) }


IVmlong_10000 =odestack[match( t_al ,odestack[,1]),Vpos+1]; #extract values for virus load at time points corresponding to experimental measurements


# correct lod

if (IVmlin[1] <= 0.3) {IVmlin[1]  <- 0.3 }

if (IVmlin[7] <= 0.3) {IVmlin[7]  <- 0.3 }

if (IVmlin[8] <= 0.3) {IVmlin[8]  <- 0.3 }
```

```
RSS_IV     <-    log( 10^IVmlin )  - log( 10^m_ivl_10000 )

loglik_IV_10000_bis  <-  -(n_days_ivl_10 /2)*  log(  (2*pi/n_days_ivl_10) * ( t( RSS_IV ) %*% RSS_IV )
) -(n_days_ivl_10 /2)

cat("loglik_IV_10000_bis ")

loglik_IV_10000_bis

IVmlin_10000  <- IVmlin

INC <- c(  L0_10000-A0_1000, 0, A0_1000,  V_1000 );

names(INC)= namevar; #assign names to parameters

odestack=NULL

IVmlin = NULL

#  odestack=try(ode( func = mvequations,  y = INC, times = t_al, parms=allparsode, method
="daspk")); #runs the ODE equations

odestack=try(lsoda( INC, t_al,mvequations,parms=allparsode, atol=atolv,rtol=rtolv)); #runs the ODE
equations

if (length(odestack)==1) {cat('!!unresolvable integrator error - triggering early return from
optimizer!!'); return(eturmerror) } #catching errors that might happen during fitting

IVmlin =odestack[match( t_ivl_10 ,odestack[,1]),Vpos+1]; #extract values for virus load at time points
corresponding to experimental measurements

if (sum(is.na(IVmlin))>0) {cat('!!ODE 10000fail VL NA return!!'); return(eturmerror) } #catching errors
that might happen during fitting

if (sum( is.infinite(10^IVmlin))>0) {cat('!!ODE 10000fail VL INF correct!!');  return(eturmerror) } #
IVmlin[ which( IVmlin>300  ) ] <-300 } #catching errors that might happen during fitting
```

Smlin=odestack[match(t_al,odestack[,1]),Spos+1]; #extract values for virus load at time points corresponding to experimental measurements

Imlin=odestack[match(t_al ,odestack[,1]),Ipos+1]; #extract values for virus load at time points corresponding to experimental measurements

Amlin_1000 =odestack[match(t_al ,odestack[,1]),Apos+1]; #extract values for virus load at time points corresponding to experimental measurements

Lmscal_1000 = Smlin + Imlin + Amlin_1000

IVmlong_1000 =odestack[match(t_al ,odestack[,1]),Vpos+1]; #extract values for virus load at time points corresponding to experimental measurements

```
# correct lod

if (IVmlin[1] <= 0.3) {IVmlin[1]  <- 0.3 }

if (IVmlin[2] <= 0.3) {IVmlin[2]  <- 0.3 }

if (IVmlin[8] <= 0.3) {IVmlin[8]  <- 0.3 }


RSS_IV     <-    log(  10^IVmlin )  - log(  10^m_ivl_1000 )

loglik_IV_1000_bis  <-  -(n_days_ivl_10 /2)*  log(  (2*pi/n_days_ivl_10) * ( t( RSS_IV ) %*% RSS_IV )  ) -(n_days_ivl_10 /2)


cat("loglik_IV_1000_bis ")

loglik_IV_1000_bis


IVmlin_1000  <- IVmlin


INC <- c(  L0_10000-A0_100, 0, A0_100,  V_100 );


names(INC)= namevar; #assign names to parameters

odestack=NULL

IVmlin = NULL
```

```
#  odestack=try(ode( func = mvequations,  y = INC, times = t_al, parms=allparsode, method ="daspk")); #runs the ODE equations

odestack=try(lsoda( INC, t_al,mvequations,parms=allparsode, atol=atolv,rtol=rtolv)); #runs the ODE equations

if (length(odestack)==1) {cat('!!unresolvable integrator error - triggering early return from optimizer!!'); return(eturmerror) } #catching errors that might happen during fitting

IVmlin =odestack[match( t_ivl_10 ,odestack[,1]),Vpos+1]; #extract values for virus load at time points corresponding to experimental measurements

if (sum(is.na(IVmlin))>0) {cat('!!ODE 10000fail VL NA return!!'); return(eturmerror) } #catching errors that might happen during fitting

if (sum( is.infinite(10^IVmlin))>0) {cat('!!ODE 10000fail VL INF correct!!');  return(eturmerror) } # IVmlin[ which( IVmlin>300  ) ] <-300 } #catching errors that might happen during fitting


Smlin=odestack[match(t_al,odestack[,1]),Spos+1]; #extract values for virus load at time points corresponding to experimental measurements

Imlin=odestack[match( t_al ,odestack[,1]),Ipos+1]; #extract values for virus load at time points corresponding to experimental measurements

Amlin_100=odestack[match(t_al ,odestack[,1]),Apos+1]; #extract values for virus load at time points corresponding to experimental measurements

Lmscal_100 = Smlin + Imlin + Amlin_100


IVmlong_100 =odestack[match( t_al ,odestack[,1]),Vpos+1]; #extract values for virus load at time points corresponding to experimental measurements


# correct lod

if (IVmlin[1] <= 0.3) {IVmlin[1]  <- 0.3 }

if (IVmlin[2] <= 0.3) {IVmlin[2]  <- 0.3 }

if (IVmlin[8] <= 0.3) {IVmlin[8]  <- 0.3 }


RSS_IV     <-    log(  10^IVmlin )  - log(  10^m_ivl_100 )

loglik_IV_100_bis  <-  -(n_days_ivl_10 /2)*  log(  (2*pi/n_days_ivl_10) * ( t( RSS_IV ) %*% RSS_IV )  ) - (n_days_ivl_10 /2)


cat("loglik_IV_100_bis ")

loglik_IV_100_bis
```

```
IVmlin_100  <- IVmlin



INC <- c(  L0_10000-A0_10, 0, A0_10,  V_10 );


names(INC)= namevar; #assign names to parameters

odestack=NULL

IVmlin = NULL



#  odestack=try(ode( func = mvequations,  y = INC, times = t_al, parms=allparsode, method ="daspk")); #runs the ODE equations

odestack=try(lsoda( INC, t_al,mvequations,parms=allparsode, atol=atolv,rtol=rtolv)); #runs the ODE equations

if (length(odestack)==1) {cat('!!unresolvable integrator error - triggering early return from optimizer!!'); return(eturmerror) } #catching errors that might happen during fitting

IVmlin =odestack[match( t_ivl_10 ,odestack[,1]),Vpos+1]; #extract values for virus load at time points corresponding to experimental measurements

if (sum(is.na(IVmlin))>0) {cat('!!ODE 10000fail VL NA return!!'); return(eturmerror) } #catching errors that might happen during fitting

if (sum( is.infinite(10^IVmlin))>0) {cat('!!ODE 10000fail VL INF correct!!');  return(eturmerror) } # IVmlin[ which( IVmlin>300  ) ] <-300 } #catching errors that might happen during fitting


Smlin=odestack[match(t_al,odestack[,1]),Spos+1]; #extract values for virus load at time points corresponding to experimental measurements

Imlin=odestack[match( t_al ,odestack[,1]),Ipos+1]; #extract values for virus load at time points corresponding to experimental measurements

Amlin_10 =odestack[match(t_al ,odestack[,1]),Apos+1]; #extract values for virus load at time points corresponding to experimental measurements

Lmscal_10 = Smlin + Imlin + Amlin_10


IVmlong_10 =odestack[match( t_al ,odestack[,1]),Vpos+1]; #extract values for virus load at time points corresponding to experimental measurements
```

```
# correct lod

if (IVmlin[1] <= 0.3) {IVmlin[1]  <- 0.3 }

if (IVmlin[2] <= 0.3) {IVmlin[2]  <- 0.3 }

if (IVmlin[8] <= 0.3) {IVmlin[8]  <- 0.3 }


RSS_IV     <-    log(  10^IVmlin )  - log(  10^m_ivl_10 )

loglik_IV_10_bis  <-  -(n_days_ivl_10 /2)*  log(  (2*pi/n_days_ivl_10) * ( t( RSS_IV ) %*% RSS_IV )  ) -
(n_days_ivl_10 /2)


cat("n_days_ivl_10 ")

n_days_ivl_10


cat("loglik_IV_10_bis ")

loglik_IV_10_bis


IVmlin_10  <- IVmlin




INC <- c(  L0_10000-A0_1, 0, A0_1,  V_1 );


names(INC)= namevar; #assign names to parameters

odestack=NULL

IVmlin = NULL



#  odestack=try(ode( func = mvequations,  y = INC, times = t_al, parms=allparsode, method
="daspk")); #runs the ODE equations

odestack=try(lsoda( INC, t_al,mvequations,parms=allparsode, atol=atolv,rtol=rtolv)); #runs the ODE
equations
```

```
if (length(odestack)==1) {cat('!!unresolvable integrator error - triggering early return from optimizer!!'); return(eturmerror) } #catching errors that might happen during fitting

IVmlin =odestack[match( t_ivl_1 ,odestack[,1]),Vpos+1]; #extract values for virus load at time points corresponding to experimental measurements


cat("sim V1 ")

IVmlin



if (sum(is.na(IVmlin))>0) {cat('!!ODE 10000fail VL NA return!!'); return(eturmerror) } #catching errors that might happen during fitting

if (sum( is.infinite(10^IVmlin))>0) {cat('!!ODE 10000fail VL INF correct!!');   IVmlin[ which( IVmlin>300 ) ] <-300 } #catching errors that might happen during fitting


Smlin=odestack[match(t_al,odestack[,1]),Spos+1]; #extract values for virus load at time points corresponding to experimental measurements

Imlin=odestack[match( t_al ,odestack[,1]),Ipos+1]; #extract values for virus load at time points corresponding to experimental measurements

Amlin_1 =odestack[match(t_al ,odestack[,1]),Apos+1]; #extract values for virus load at time points corresponding to experimental measurements

Lmscal_1 = Smlin + Imlin + Amlin_1


IVmlong_1 =odestack[match( t_al ,odestack[,1]),Vpos+1]; #extract values for virus load at time points corresponding to experimental measurements


# correct lod

if (IVmlin[1] <= 0.3) {IVmlin[1]  <- 0.3 }

if (IVmlin[2] <= 0.3) {IVmlin[2]  <- 0.3 }

if (IVmlin[3] <= 0.3) {IVmlin[3]  <- 0.3 }


RSS_IV     <-    log(  10^IVmlin )  - log(  10^m_ivl_1 )

loglik_IV_1_bis  <-  -(n_days_ivl_1 /2)*  log(  (2*pi/n_days_ivl_1) * ( t( RSS_IV ) %*% RSS_IV )  ) - (n_days_ivl_1 /2)
```

```
cat("n_days_ivl_1 ")
n_days_ivl_1


cat("loglik_IV_1_bis ")
loglik_IV_1_bis


IVmlin_1  <- IVmlin


odestack=NULL
IVmlin = NULL


loglik_bis <-  loglik_IV_10000_bis   +  loglik_IV_1000_bis   +loglik_IV_100_bis   + loglik_IV_10_bis   +
loglik_IV_1_bis



cat("loglik_bis  ")
loglik_bis



cat("loglik_ref  ")
MSLL_resmatrix["LOGLIK"]
#####################

#####################################################




#MLE <- data.table(param = parnames,
#               estimates = MLE_par,
#              sd = MLE_SE,
#             aicc =  MLE_par*0+ datasim$AICC[1],
```

```
#           loglik = MLE_par*0-MLE_estimates$value )

#            TABMLE  <-  kable(MLE)

#           kable(data.table(MSLL_resmatrix))




datafit  <- data.frame(  TCID = c( t_al*0+10^4,   t_al*0+10^3,t_al*0+10^2, t_al*0+10^1, t_al*0+10^0)
,
                    X50. =  c( IVmlong_10000,IVmlong_1000, IVmlong_100, IVmlong_10, IVmlong_1  ) ,
                    #mes =  c( m_ivl_10000, m_ivl_1000, m_ivl_100, m_ivl_10, m_ivl_1  ) ,
                    tlym = c(Lmscal_10000, Lmscal_1000,Lmscal_100,Lmscal_10,Lmscal_1 ),
                    tcell = c(Amlin_10000, Amlin_1000, Amlin_100, Amlin_10, Amlin_1    ),
                    t =   c(  t_al, t_al, t_al, t_al,   t_al   ) )



####################
library(forcats)

data94$categ <- fct_rev( as.factor( data94$TCID))

datafit$categ <- fct_rev( as.factor( datafit$TCID))





FIG_ICIC <-     ggplot(  data = datafit, aes(x = t, y = X50.,  color=categ, fill=categ,   group=categ ) ) +
  geom_line(  size =1.25) +
  geom_point(data = data94,  aes(x = t, y = mes,   color=categ, fill=categ, shape=categ),     size=2) +
  scale_shape_manual( values=c( 18, 8,17,19,  15)) +
```

```
  scale_fill_manual(values=cbbPalette) +
  scale_color_manual(values=cbbPalette) +
  labs( shape = bquote(  TCID[50] ~ "="), fill = bquote(  TCID[50] ~ "="), color = bquote(  TCID[50] ~
"=") ) +
  #geom_ribbon(aes(ymin = X5., ymax = X95.), fill = "orange", alpha = 0.35) +
  #geom_point(data = data94,  aes(x = t, y = mes,   color=as.factor(TCID)),   size=2) +
  #geom_line(mapping = aes( y = si,m_ivl),linetype = "dashed",size=2, color = "blue") +
  labs(x = "Days post MV infection", y =bquote(  "Log" ~  TCID[50] ~  "/" ~  10^6 ~  "PBMC"))+
  ggtitle("Infectious viremia ")  +
  #annotate(geom="text", x=25, y=4, label=mcqii, color="black")+
  theme(legend.position="bottom")   +
  theme(legend.text = element_text(colour="black",  face="bold")) +
  geom_hline(yintercept = 0.3, linetype = "dotdash", color = "darkgray", size=1) +
  scale_x_continuous( limits=c(0, 30),  breaks=c(0,  3,  5,  7,  9,  13,  17, 20,25,30 ))+
  scale_y_continuous(limits = c(0,4),breaks=c(0.3,1,2,  3,3.4,  4 ))+
  annotate(geom="text", x=8, y=4,size=2.5,
         label= paste("A2: MLE=",round(as.numeric(MSLL_resmatrix["LOGLIK"] ),2), sep=""),
         color="black")+
  annotate(geom="text", x=23, y=4,size=2.5,
         label= paste("AICc=",round(as.numeric(MSLL_resmatrix["AICC"] ),2), sep=""),
         color="black")  +
  theme(legend.position = "none")


cat("best fit ")
MSLL_resmatrix

FIG_ICIC


#########################################
```

```
mdlfrnABIC <- plot_grid(  FIG_IC,  FIG_ICIC,
                          FIG_DI,  FIG_ICDIlg,
                         labels = c("a", "b","c", "d"),
                         align="h", ncol=2,
                         label_size = 11)

mdlfrnABIC
#######################

######################
"savecombinefigdynamics"
save_plot(paste("f-mv-model_1994_hpcMS_Fig2.pdf", sep=""), mdlfrnABIC,  ncol = 2, nrow = 2 )

#Save the ggplot as an EPS file
ggsave("Fig2.eps", plot = mdlfrnABIC, device = "eps", width = 4.5, height = 5, units = "in")

save_plot(paste("Fig2.pdf", sep=""), mdlfrnABIC,  ncol = 2, nrow = 2 )



 # Function to compute area under the curve using trapezoidal rule
 compute_auc <- function(x, y) {
   n <- length(x)
   width <- diff(x)
   height <- (y[-1] + y[-n]) / 2
   auc <- sum(width * height)
   return(auc)
 }
 # Load the dplyr package
```

```
library(dplyr)


data94$mesln <-   log2( 10^(data94$mes) )

# Compute area under the curve for each TCID
auc_results <- data94 %>%
  group_by(TCID) %>%
  summarise(auc = log2( compute_auc(t, mes)))

# Print the results
print(auc_results)


# Your data
data <- data.frame(
  TCID = c(1, 10, 100, 1000, 10000),
  auc = c(4.63, 4.66, 4.66, 4.43, 4.61)
)

# Perform a trend test
trend_test <- cor.test(data$TCID, data$auc, alternative = "two.sided", method = "spearman")

# Print the test results
print(trend_test)


## AUC detectable

data94DEC <- subset ( data94,  data94$mes > 0.3)
data94DEC
```

```
# natural log in r - example



# Compute area under the curve for each TCID
auc_results <- data94DEC %>%
  group_by(TCID) %>%
  summarise(auc = log2(compute_auc(t, mesln )))

# Print the results
print(auc_results)


###########
tryd <- data.frame( mes = ( c(3,8, 200,  1000,  3,3 )  ),
                    t = c(0,3, 5,  9,  13,17   ) )


# Compute area under the curve for each TCID
auc_results <-   tryd %>%
  summarise(auc = (compute_auc(t, mes) ))

# Print the results
print(auc_results)

########################################


data94$t
```

```
data94$ts <- c(0+6 , 3+6  ,5+6,  7+6,  9+6, 11+6, 14+6, 18+6, 0+4,  3+4,  5+4,  7+4,  9+4, 11+4, 14+4, 
18+4,0+4,  3+4,  5+4,  7+4,  9+4, 11+4, 14+4, 18+4, 0+4,  3+4,  5+4,  7+4,  9+4, 11+4, 14+4, 18+4, 0,  3,  
6,  9, 13, 17)

data94$tss <- c(  0+2,  3+2,  5+2,  7+2,  9+2, 11+2, 14+2, 18+2,
0,  3,  5,  7,  9, 11, 14, 18,
0,  3,  5,  7,  9, 11, 14, 18,
0,  3,  5,  7,  9, 11, 14, 18,
0-4,  3-4,  6-4,  9-4, 13-4, 17-4 )

fig_raw <-  ggplot( data = data94,  aes(x = t, y = mes,  color=categ, fill=categ,   group=categ ) ) +
  geom_point( aes(  shape=categ ),  size=2 )+
  scale_shape_manual( values=c( 18, 8,17,19,  15)) +
  scale_fill_manual(values=cbbPalette) +
  scale_color_manual(values=cbbPalette) +
    labs( shape = bquote(  TCID[50] ~ "="), fill = bquote(  TCID[50] ~ "="), color = bquote(  TCID[50] ~ 
"=") ) +
      #geom_ribbon(aes(ymin = X5., ymax = X95.), fill = "orange", alpha = 0.35) +
  geom_line(  size =0.75) +
  #geom_point(data = data94,  aes(x = t, y = mes,   color=as.factor(TCID)),   size=2) +
  #geom_line(mapping = aes( y = si,m_ivl),linetype = "dashed",size=2, color = "blue") +
  labs(x = "Days post MV infection", y =bquote(  "Log" ~  TCID[50] ~  "/" ~  10^6 ~  "PBMC"))+
  ggtitle("Infectious viremia ")  +
  #annotate(geom="text", x=25, y=4, label=mcqii, color="black")+
  theme(legend.position="bottom")   +
  theme(legend.text = element_text(colour="black",  face="bold")) +
  geom_hline(yintercept = 0.3, linetype = "dotdash", color = "darkgray", size=1) +
  scale_x_continuous(  breaks=c(0,  3,  5,6,  7,  9, 11, 13, 14, 17, 18 ))+
scale_y_continuous(limits = c(0,4),breaks=c(0.3,1,2,  3,3.4,  4 ))+
  theme(legend.position = "none")
```

```
fig_SH <-     ggplot( data = data94,  aes(x = tss, y = mes,  color=categ, fill=categ,   group=categ ) ) +
  geom_point( aes(  shape=categ ),  size=2 )+
  scale_shape_manual( values=c( 18, 8,17,19,  15)) +
  scale_fill_manual(values=cbbPalette) +
  scale_color_manual(values=cbbPalette) +
  labs( shape = bquote(  TCID[50] ~ "="), fill = bquote(  TCID[50] ~ "="), color = bquote(  TCID[50] ~
"=") ) +
  #geom_ribbon(aes(ymin = X5., ymax = X95.), fill = "orange", alpha = 0.35) +
  geom_line(  size =0.75) +
  #geom_point(data = data94,  aes(x = t, y = mes,   color=as.factor(TCID)),   size=2) +
  #geom_line(mapping = aes( y = si,m_ivl),linetype = "dashed",size=2, color = "blue") +
  labs(x = " ", y =bquote(  "Log" ~  TCID[50] ~  "/" ~  10^6 ~  "PBMC"))+
  ggtitle("Aligning the peaks of infectious viremia ")  +
  #annotate(geom="text", x=25, y=4, label=mcqii, color="black")+
  theme(legend.position="bottom")   +
  theme(legend.text = element_text(colour="black",  face="bold")) +
  geom_hline(yintercept = 0.3, linetype = "dotdash", color = "darkgray", size=1) +
  scale_x_continuous(breaks=c(-4: 18 ))+
  scale_y_continuous(limits = c(0,4), breaks=c(0.3,1,2,  3,3.4,  4 ))+
  theme(    axis.text.x=element_blank() )



################################
######lin trial linear regressions
```

```
## extracting data  for expansion

dexp <- data94$mes[ which(data94$tss <=  9  & data94$mes > 0.3)]

tmdexp <- data94$tss[ which(data94$tss <=  9  & data94$mes > 0.3)]

unique(tmdexp)

max(dexp )
min(dexp )



plot(tmdexp  ,  dexp  )



ddecl <- data94$mes[ which(data94$tss >=  9  & data94$mes > 0.3)]



tmddecl <- data94$tss[ which(data94$tss >=  9  & data94$mes > 0.3)]

unique(tmddecl)

plot(tmddecl ,  ddecl  )



max(ddecl )
min(ddecl)

############ perform regressions
unique(tmdexp)
```

```
lm_growth  <-lm(dexp  ~ tmdexp  )
summary(lm_growth )

lrnexp_value


#   0.56725

coef <- coef(lm_growth )[2]
p_val <- summary(lm_growth )$coefficients[2, 4]

# -0.48618


# Get the summary of the model
summary_lm <- summary(  lm_growth )

# Extract the standard errors of coefficients

coefficients <- summary_lm$coefficients[, "Estimate"]
coeff_standard_errors <- summary_lm$coefficients[, "Std. Error"]

# Extract the intercept and slope (coefficient of x)
intercept <- coefficients[1]
slope <- coefficients[2]

lr_exp <- slope*  c(5,9,10) +intercept

lr_exp

df_lr_exp = data.frame(categ = as.factor(c(1,1,1)),tt = c(5,9,10), yy= lr_exp)
```

```
# Extract the standard deviations for intercept and slope
intercept_std_dev <- coeff_standard_errors[1]
slope_std_dev <- coeff_standard_errors[2]

# Print the values and standard deviations
cat("Intercept:", intercept, " (Standard Deviation:", intercept_std_dev, ")\n")
cat("Slope (Coefficient of x):", slope, " (Standard Deviation:", slope_std_dev, ")\n")


# Intercept: -3.967952  (Standard Deviation: 0.3642367 )
#   Slope (Coefficient of x): 0.5672499  (Standard Deviation: 0.03272828 )




lm_dec  <-lm(ddecl  ~ tmddecl )
summary(lm_dec )



# Get the summary of the model
summary_lm <- summary(  lm_dec  )

# Extract the standard errors of coefficients


coefficients <- summary_lm$coefficients[, "Estimate"]
coeff_standard_errors <- summary_lm$coefficients[, "Std. Error"]

# Extract the intercept and slope (coefficient of x)
intercept <- coefficients[1]
slope <- coefficients[2]
```

```
lr_de <- slope*  c(8,9,14) +intercept

lr_de

df_lr_de = data.frame(categ = as.factor(c(1,1,1)), tt = c(8,9,14), yy= lr_de)



# Extract the standard deviations for intercept and slope
intercept_std_dev <- coeff_standard_errors[1]
slope_std_dev <- coeff_standard_errors[2]

# Print the values and standard deviations
cat("Intercept:", intercept, " (Standard Deviation:", intercept_std_dev, ")\n")
cat("Slope (Coefficient of x):", slope, " (Standard Deviation:", slope_std_dev, ")\n")
summary(lm_dec )

# Intercept: 9.718297  (Standard Deviation: 1.070327 )
#Slope (Coefficient of x): -0.4861767  (Standard Deviation: 0.06976746 )



fig_SH <-   fig_SH  +
  geom_line( data = df_lr_de,  aes(x = tt, y = yy ), color ="black", linetype = "dashed" ) +
  geom_line( data = df_lr_exp,  aes(x = tt, y = yy ), color ="black" )


################################################################
```

```
data94_charac <- data.frame(  TCID = c( 10^4,  10^3, 10^2, 10^1, 10^0)  ,
                        tpeak = c(  7,  9, 9, 9, 13)  ,
                   #         auc = c(  24.4,  21.6, 25.3, 25.2, 24.7)  )
                auc = c(19.7,  17.5, 20.2, 20.7, 20.9)  )




fig_auc <-  ggplot() +
  #geom_ribbon(aes(ymin = X5., ymax = X95.), fill = "orange", alpha = 0.35) +
  geom_point(data = data94_charac,  aes(x = TCID , y = auc, color=as.factor(TCID)),    size=2.5) +
  #geom_line(mapping = aes( y = si,m_ivl),linetype = "dashed",size=2, color = "blue") +
  labs(x = bquote(TCID[50]), y = "AUC of viremia")+
    #annotate(geom="text", x=25, y=4, label=mcqii, color="black")+
  labs(color =  bquote(TCID[50] ~  "=" )) +
  scale_fill_manual(values=cbbPalette)+
  theme(legend.position = "none") +
 # geom_hline(yintercept = 0.3, linetype = "dashed", color = "gray", alpha=0.5) +
scale_x_log10(breaks=c(10^4,  10^3, 10^2, 10, 1 ))+
scale_y_continuous(limit =c( 17, 21 ))

# geom_smooth(method = "lm", se = FALSE)  # Linear regression line (no confidence interval)



fig_tp <-  ggplot() +
  #geom_ribbon(aes(ymin = X5., ymax = X95.), fill = "orange", alpha = 0.35) +
  geom_line(data = data94_charac, aes(x =  TCID, y =   tpeak, color="black" ),  size =1) +
      geom_point(data = data94_charac,  aes(x = TCID , y =   tpeak , color=as.factor(TCID)),     size=2) +
  #geom_line(mapping = aes( y = si,m_ivl),linetype = "dashed",size=2, color = "blue") +
```

```
  labs(x = bquote(TCID[50]), y = "Day of the peak viremia")+
  #annotate(geom="text", x=25, y=4, label=mcqii, color="black")+
  labs(color =  bquote(TCID[50] ~  "=" )) +
  scale_fill_manual(values=cbbPalette)+
  theme(legend.position = "none") +
  geom_hline(yintercept = 0.3, linetype = "dashed", color = "darkgray") +
 # scale_x_log10(breaks=c(10^4,  10^3, 10^2, 10, 1 ))+
scale_y_continuous(breaks=c(5,7,9,13,15 ),  limit =c( 5, 15 ))+
scale_x_log10(breaks=c(10^4,  10^3, 10^2, 10, 1 ))






"savecombinefigdynamics"
save_plot(paste("f-mv-model_1994_hpcMS_shifts.pdf", sep=""), fig_SH ,  ncol = 1, nrow = 1 )



mdlfrnD<- plot_grid(  fig_raw,    fig_tp, fig_SH ,   fig_auc ,
                   labels = c("a", "b", "c", "d"),
                      align="h", ncol=2,
                      label_size = 11)


mdlfrnD
save_plot(paste("f-mv-data_1994_features_tss.pdf", sep=""), mdlfrnD ,  ncol = 2, nrow = 2 )
```

```
mdlfrnD2 <- plot_grid(  fig_raw,   fig_SH,
                    labels = c("a", "b"),
                    align="h", ncol=1,
                    label_size = 11)

mdlfrnD2

save_plot(paste("f-mv-data_1994_Fig1.pdf", sep=""), mdlfrnD2 ,  ncol = 1, nrow = 2 )

mdlfrnD2h <- plot_grid(  fig_raw,   fig_SH,
                          align="h", ncol=2,
                     label_size = 11)

mdlfrnD2h

mdlfrnD2 <- plot_grid(  fig_raw,   fig_SH,
                     labels = c("a", "b"),
                     align="h", ncol=1,
                     label_size = 11)

mdlfrnD2

save_plot(paste("f-mv-data_1994_pres1.pdf", sep=""), mdlfrnD2 ,  ncol = 1, nrow = 2 )

# Save the ggplot as an EPS file
ggsave("Fig1.eps", plot = mdlfrnD2, device = "eps", width = 4.5, height = 5, units = "in")
save_plot(paste("Fig1.pdf", sep=""), mdlfrnD2 ,  ncol = 1, nrow = 2 )
```

```
mdlfrnDAL <- plot_grid(    fig_A  , fig_L,
                    labels = c("a", "b"),
                    align="h", ncol=1,
                    label_size = 11)

mdlfrnDAL

save_plot(paste("f-mv-data_1994_Fig4.pdf", sep=""), mdlfrnDAL ,  ncol = 1, nrow = 2 )




COR3 <- plot_grid(  T0V0, T0A0 , A0TTP,
                     labels = c("B1", "B2","B3"),
                     align="h", ncol=3,
                     label_size = 12)

mdlfrnCOR
######################



  FIG_ICDIp  <-  FIG_ICDIp  + theme(
    legend.text = element_text(size = 12),  # Legend font size
    legend.title = element_text(size = 12), # Legend title font size
    plot.title = element_text(size = 12),   # Plot title font size
    axis.title.x = element_text(size = 12), # X-axis label font size
    axis.title.y = element_text(size = 12)  # Y-axis label font size
  )
```

```
FIG_KIAp  <-  FIG_KIAp  + theme(
  legend.text = element_text(size = 12),  # Legend font size
  legend.title = element_text(size = 12), # Legend title font size
  plot.title = element_text(size = 12),   # Plot title font size
  axis.title.x = element_text(size = 12), # X-axis label font size
  axis.title.y = element_text(size = 12)  # Y-axis label font size
)



fig_Lp  <-  fig_Lp  + theme(
  legend.text = element_text(size = 12),  # Legend font size
  legend.title = element_text(size = 12), # Legend title font size
  plot.title = element_text(size = 12),   # Plot title font size
  axis.title.x = element_text(size = 12), # X-axis label font size
  axis.title.y = element_text(size = 12)  # Y-axis label font size
)



figpos <- plot_grid(   FIG_ICDIp ,COR3,   FIG_KIAp  , fig_Lp,
                  labels = c("A", " ", "C", "D"),
                  align="h", ncol=1,
                  label_size = 12)


save_plot(paste("f-mv-data_1994_poster.pdf", sep=""), figpos,  ncol = 1, nrow = 4 )
```

figpos

############
